# Applying a Theory of Change for Human Resources Development in Public Health Supply Chains in Rwanda

**DOI:** 10.9745/GHSP-D-23-00062

**Published:** 2025-05-09

**Authors:** Erin Meier, Andrew N. Brown, Bridget McHenry, Joseph Kabatende, Inès K. Gege Buki, Joyce Icyimpaye

**Affiliations:** a IntraHealth International, Chapel Hill, NC, USA.; b Management Sciences for Health, Canberra, Australia.; c Global Health Training, Advisory, and Support Contract, a Public Health Institute contract for U.S. Agency for International Development, Washington, DC, USA.; d Rwanda Food and Drugs Authority, Kigali, Rwanda.; e Chemonics International, Kigali, Rwanda.

## Abstract

**Background:**

The health supply chain (SC) system in Rwanda experienced a number of workforce-related challenges, including insufficient skilled supply chain management (SCM) professionals with the necessary competencies. The Human Resources for Supply Chain Management (HR4SCM) Theory of Change (TOC) provides a methodology to assess human resources (HR) management systems by explaining the preconditions required to achieve optimized workforce performance. We applied this model to design interventions to strengthen the Rwanda health SC workforce.

**Methods:**

We compared conditions in the health SC HR system in Rwanda with the 60 outcomes described as necessary for optimized workforce performance in the HR4SCM TOC model. We used a survey and participatory workshop at the central level, followed by structured interviews (N=35) with SC professionals in health centers, hospitals, and regional warehouses (N=20) in Southern Province and Kigali City to identify which outcomes already existed in the Rwandan HR system and which outcomes required strengthening through targeted interventions. We used focus groups (N=2) to refine interventions.

**Findings:**

We identified that 31 of the 60 outcomes were not sufficiently in place in the Rwandan health SC HR system. SCM workers had gaps in the technical and managerial competencies and did not have access to adequate training and professional development opportunities for certain required competencies. An SCM career path did not exist, and education was not available for all required SCM qualifications. Fourteen of these outcomes were prioritized for strengthening. We designed 20 workforce interventions with the Ministry of Health to address these deficiencies and selected indicators to monitor the interventions.

**Conclusion:**

Applying this HR TOC model enabled a systematic process to identify gaps, develop and prioritize interventions, and select indicators. Practitioners designing and evaluating SC workforce interventions should consider applying this methodology to design more effective, theory-driven interventions to improve SC workforce performance.

## Abstract (precis)

The Human Resources for Supply Chain Management Theory of Change model enables users to assess how a country’s existing supply chain human resources system compares to the conditions necessary for optimized supply chain management workforce performance.

## Teaser

**Key Findings:**

- To strengthen the human resources (HR) in the Rwanda health supply chain (SC) system, the Human Resources for Supply Chain Management (HR4SCM) Theory of Change (TOC) model was used to assess gaps in 4 pathways: staffing, skills, working condition, and motivation.
- Of the total 60 outcomes described for an optimized SC workforce, 26 outcomes existed in the Rwanda system, but 31 outcomes were deficient, including insufficient budget to fund SC positions, lack of technical and managerial competencies, lack of necessary tools and equipment, and insufficient mechanisms and skills to communicate supervisor feedback.
- Using the HR4SCM TOC model enabled the Rwanda Ministry of Health to acknowledge gaps in the system, enhance understanding among decision-makers on the complex factors that affect workforce performance, and select interventions to address the gaps.

**Key Implications:**

- Practitioners should consider using human resources TOC models, such as the HR4SCM TOC, to assess HR management systems for health SCs and design workforce interventions.
- Governments and technical partners can use the TOC model to articulate the complexities that govern HR in health SCs and to identify how interventions can improve the staffing, skills, motivation, and working conditions of this workforce.

## INTRODUCTION

Strong public health supply chains (SCs) rely on a motivated, supported, and skilled workforce to effectively operate the SC.^1^^,^^2^ Insufficient numbers of competent staff can cause breakdowns in SC systems and lead to poor system performance.^1^^,^^3^^–^^7^ Many governments face a shortage of SC human resources (HR), partly due to insufficient staff positions, budget cuts, and the migration of skilled individuals.^3^^–^^6^ In many situations, the cadres responsible for the supply chain management (SCM) of health products have not received sufficient education or practical training to fulfill the competencies required.^7^^–^^11^ The most common barriers countries face in HR within public health SCs include an absence of dedicated SCM leadership, a lack of training capacity in SCM, a lack of performance support and motivation for logistics tasks, high staff turnover and mobility, and high workload among health personnel.^4^^,^^5^^,^^12^^–^^14^

**FIGURE 1:**
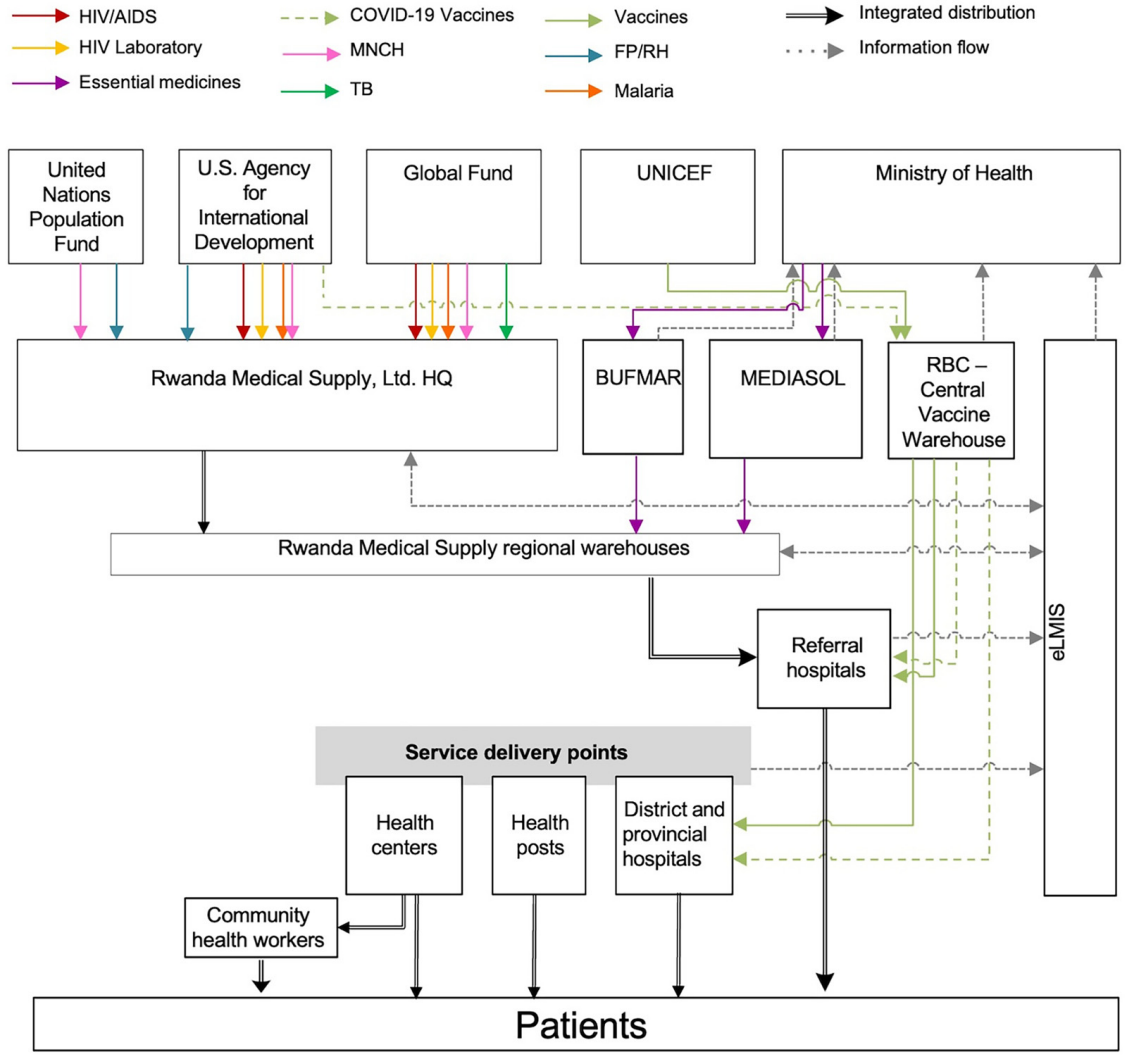
Overview of the Public Health Supply Chain in Rwanda Abbreviations: BUFMAR, Bureau des Formations Médicales Agréées du Rwanda; FP/RH, family planning/reproductive health; HQ, headquarters; MEDIASOL, Medical & Allied Service solutions; MNCH, maternal, newborn, and child health; RBC, Rwanda Biomedical Centre; RMS, Rwanda Medical Supply.

The Ministry of Health (MOH) of Rwanda coordinates the supply of health commodities through the Rwanda Medical Supply Ltd (RMS Ltd), a private, government-owned central medical store company that reports to and is supervised by the MOH. At the time of data collection for this research, the Medical Procurement and Production Division (MPPD), which reported to the MOH, was the public central medical store entity responsible for supplying and distributing health commodities and was overseen by the Rwanda Biomedical Centre, which was under the umbrella of national disease programs. In the time since data collection occurred, MPPD has transformed into the RMS Ltd, which collaborates with the Bureau des Formations Médicales Agréées du Rwanda and the Medical & Allied Service solutions mainly to avail health commodities to the public health SC in Rwanda.^15^ At the district level, RMS Ltd distributes products to 30 RMS regional warehouses (which were referred to as district pharmacies at the time of this research), 8 referral or teaching hospitals, district hospitals, health centers, and health posts.^15^ The Bureau des Formations Médicales Agréées du Rwanda, a faith-based organization, operates as a central medical store to distribute health products to faith-based health facilities (Figure 1).

In Rwanda, health SCs are a key enabler for health programs; however, the availability of medicines is poor in the public sector.^16^^,^^17^ Ensuring enough skilled SCM professionals are available to the MOH is an ongoing challenge, and many barriers exist.^18^^,^^19^ For example, before this research, the MOH staff organogram included only 1 SCM position.^20^ Although all 30 RMS regional warehouses in Rwanda employed qualified pharmacists responsible for SCM, the tasks and functions of these positions were not properly articulated. In addition, SCM competency frameworks and SCM-specific career paths had not been developed for all SCM cadres. To mitigate these challenges, strategies have been implemented to strengthen the recruitment, capacity development, and motivation of personnel.^18^^,^^21^ Approaches for building capacity have been deployed, including a supportive supervision program, mentorship, and continuous learning through a people-centered approach.^18^^,^^21^^,^^22^

### Human Resources for Supply Chain Management Theory of Change Model

**FIGURE 2:**
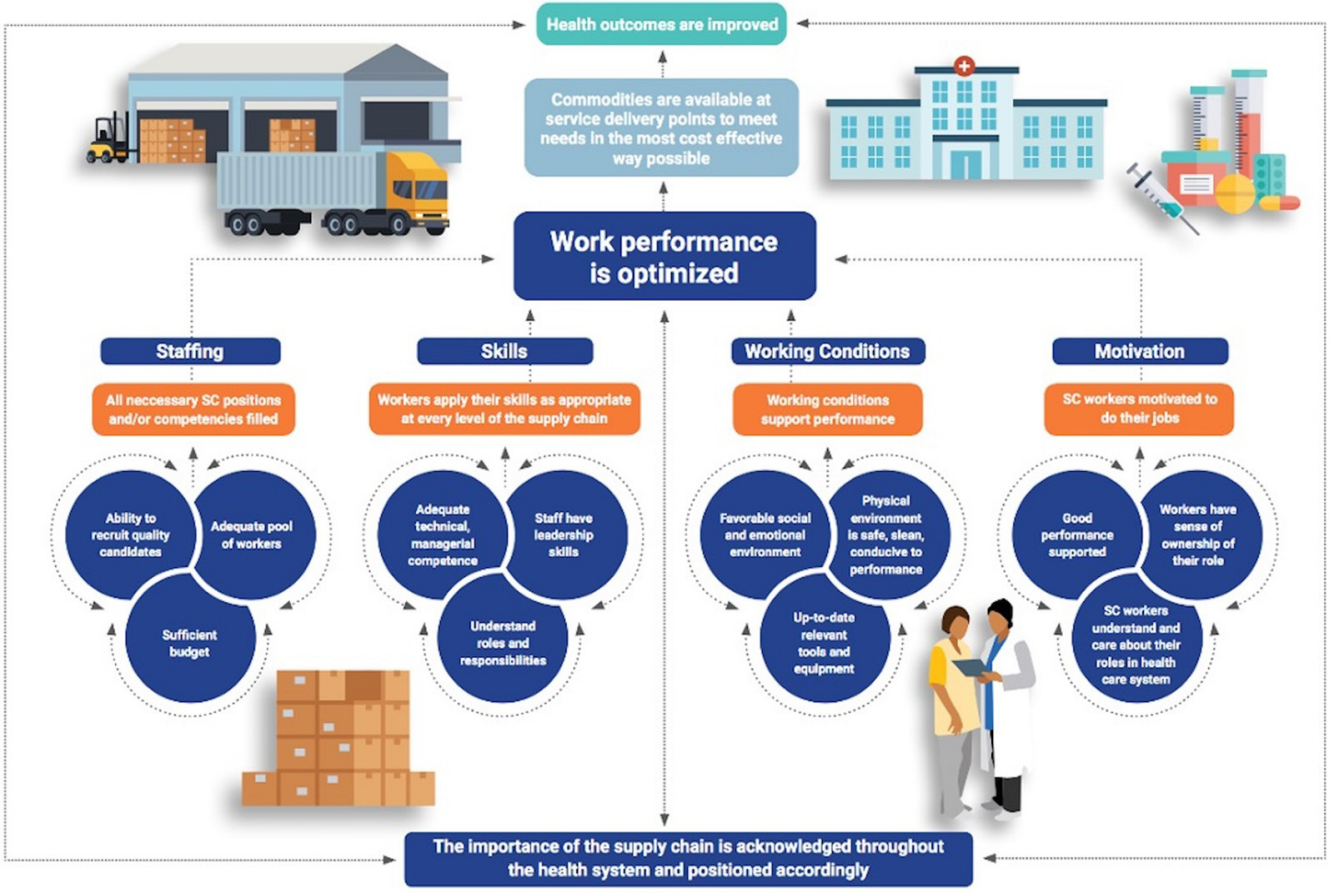
Simplified Version of the Human Resources for Supply Chain Management Theory of Change Model

Strengthening the health SC workforce requires both simple and complex interventions implemented in a logical progression (some interventions, for example, should be carried out in a particular order) to ensure an adequate number of staff with the appropriate skills, motivation, and working conditions are employed with the ultimate aim of optimizing workforce performance so that health commodities are available to meet the needs of the population, including at the last mile.^23^ However, the complex nature of HR systems makes effective interventions difficult to design, and multiple factors can make their impact difficult to attribute and measure.^24^ The People that Deliver (PtD) Human Resources for Supply Chain Management (HR4SCM) Theory of Change (TOC) model provides a systematic approach to developing HR interventions (Figure 2). This TOC model focuses on the HR component of public health SCs and explains how various HR factors work together in a causal pathway to optimize the performance of a country’s public health SC workforce.^23^

Strengthening the health SC workforce requires both simple and complex interventions implemented in a logical progression.

A TOC visually depicts all outcomes needed to achieve a long-term program goal; the outcomes are arranged in a causal framework, showing how outcomes contribute to the goal. The TOC approach is widespread in the public health literature. Breuer et al. retrieved 62 papers in which a TOC was used in the development or evaluation of public health interventions.^25^ Many researchers have captured the process to develop a TOC^25^; however, few articles have used the mapped outcomes in an existing TOC to design interventions. The use of TOC approaches to explain health SCM systems is limited, and, where used, the HR component is oversimplified, considering the body of HR4SCM literature.

The HR4SCM TOC model was developed through an extensive, collaborative process involving multiple iterations of the causal framework’s components. The model’s components were created through rounds of input from experts in health SCM, experts in TOC methodology, and public health SC stakeholders.^23^ To generate the model, developers first defined the framework’s long-term goal—that SCM workforce performance is optimized. Next, developers defined the prerequisite outcomes that would be both necessary and sufficient to reach this goal. Ultimately, 4 component parts were defined: (1) all critical SC positions are filled (staffing), (2) workers are able to apply their skills at every level of the SC (skills), (3) working conditions help employees to reach their potential (working conditions), and (4) SC workers are motivated to do their jobs (motivation).^23^ Next, developers defined and mapped all outcomes hypothesized to be necessary to create these 4 preconditions. Developers traced out the causal connections between all identified outcomes across 4 pathways, ensuring that outcomes were both necessary and sufficient to meet the next set of outcomes. Finally, developers reviewed the overall outcomes map to check that the logic was coherent and identify any gaps in preconditions. They outlined the rationales for why a certain outcome is a precondition for another and clarified the model’s assumptions.^23^ The TOC diagram was finalized after rounds of feedback from experts.

This TOC model defines 4 pathways (staffing, skills, working conditions, motivation) that must be strengthened simultaneously to reach the long-term goal. Each pathway contains a progression of outcomes that must be developed sequentially to obtain the top-level outcome of each pathway. The full TOC diagram depicting 60 outcomes suggested as minimal conditions for optimized SCM workforce performance is shown in Supplement 1. The HR4SCM TOC narrative^23^ also outlines interventions to develop outcomes where they are absent.

Realizing the Rwandan MOH’s vision for a well-trained, motivated workforce required interventions to improve HR for SCM. This research aimed to test the HR4SCM TOC model as a method for identifying workforce interventions in Rwanda and selecting indicators. It was hypothesized that Rwanda MOH would be able to prioritize HR4SCM investments to improve the availability of the skilled cadres required for effective health SCM. In addition, this research provided indicators to monitor the performance of HR systems within the government health SC.

## METHODS

The HR4SCM TOC model was selected as a standardized benchmark to assess the HR management system that supports the integrated health SC in Rwanda. As this TOC model theorizes the minimal conditions required for optimized health SCM workforce performance,^23^ we hypothesized we could apply the logic of this model to assess the HR system and to design interventions based on solid rationale. We assessed which of the TOC model’s 60 prescribed outcomes already existed in the Rwandan HR system and which TOC outcomes required strengthening in the Rwanda context through targeted interventions.

This research was conducted from October to November 2018. A qualitative approach, which engaged HR and SCM personnel across SC levels, was determined to be the best method to gather information on whether TOC model outcomes existed in the Rwanda context and to understand the status of HR and SCM conditions.

The approach included 3 steps. First, we used a survey and participatory workshop to determine which of the 60 TOC outcomes existed in the HR system. Second, we conducted 20 structured interviews with staff members at central, district, and health facility levels to confirm the status of outcomes suspected not to exist. Third, we conducted 2 focus group discussions to select and prioritize interventions.

### Data Collection

#### Step 1: Identifying Well-Established Outcomes

The HR4SCM TOC model contains 60 outcomes representing the suggested minimal conditions required for optimized SCM workforce performance (Supplement 1). To identify well-established outcomes, 2 MOH staff completed a close-ended, written survey to indicate whether each outcome existed in the HR system. Next, 6 participants from the MOH, Rwanda Biomedical Centre-Medical Production and Procurement Division (which has since transformed into RMS Ltd), and the Global Health Supply Chain Program-Procurement and Supply Management project participated in a 1-day workshop to confirm survey results, select priority outcomes to develop, and choose indicators to collect baseline data for prioritized outcomes. Workshop participants reviewed the HR4SCM TOC’s Indicators and Interventions Catalog (an annex to the HR4SCM TOC document^23^) to select indicators that could be used to collect baseline data during site visits.

The HR4SCM TOC model contains 60 outcomes representing the suggested minimal conditions required for optimized SCM workforce performance.

#### Step 2: Conducting Interviews to Confirm Deficient Outcomes

The researchers conducted site visits to capture data for selected outcomes through in-person, structured group interviews. Data were collected from 2 areas: City of Kigali and Southern Province. These areas were selected through stratified opportunistic sampling based on accessibility and priority given by the Rwanda MOH. We asked MOH workers to assist in identifying sites. We selected 20 health SCM sites where we were most likely to obtain the targeted indicator information. As HR policies and processes are determined at the national level in Rwanda, we expected findings would be relatively similar across facilities. We stratified by SC level (national, district, and health facility levels) to ensure all levels of the SC system in Rwanda were represented. We purposively chose workers responsible for or overseeing HR and SCM activities at the selected sites (i.e., the SCM worker, HR staff member, and head of facility). At each site, 1–3 staff were interviewed in a group interview lasting approximately 1 hour.

We developed a close-ended questionnaire that included at least 1 question for each TOC outcome selected during Step 1. The questionnaire response options were typically yes/no and asked about whether policies, systems, or practices existed for various topics, such as HR management processes, work environment, and supervision. We also reviewed documents, including recruitment reports, policies and procedures, performance evaluation reports, and job descriptions, to complement information obtained from the interviews. One data collector took notes on all interviews and captured policies and documentation that supported participants’ responses. We used these data to classify which model outcomes were lacking and which outcomes were already in place.

#### Step 3: Selecting Appropriate Interventions

One focus group was convened in each research area (Kigali and Southern Province) to discuss interventions for gaps identified in site visits and to validate site visit data. All interview participants were invited to participate in a focus group. The Kigali focus group included 7 participants; 2 of these participants had not participated in the interviews. The Southern Province focus group convened 21 staff members from the 15 sites visited. To manage the large group in Southern Province, we divided participants into 2 discussion groups and assigned 2–3 of the 5 HR managers attending to each small group.

The researchers shared the key gaps for each of the 4 pathways (staffing, skills, working conditions, and motivation) identified from the site visits with participants, along with key questions related to the presented pathway. The researchers also shared the related interventions from the HR4SCM TOC Interventions and Indicators Catalog^23^ (the Catalog) as a starting point. The participants discussed barriers to meeting the identified gaps, the impact of the gaps, and actions that could improve the gaps. Participants proposed additional interventions and provided feedback on interventions from the Catalog through focus group discussions. Interventions from the Catalog were refined through focus group discussion and by consideration of the key gaps identified in Step 2 of this research.

#### Step 4: Prioritization of Interventions

To prioritize outcomes for intervention, we first generated a ranking score for each outcome to help make sense of the 31 deficient outcomes. The score had 3 components: (1) whether the outcome existed (no/yes); (2) priority level determined by focus group discussion (high/medium/low); (3) whether a lower-level outcome first needed to be developed before this outcome could be targeted (no/yes). Ranked outcomes were reviewed by the MOH to select priority intervention areas. We also compared the prioritized final outcomes against the TOC framework to ensure that no critical, lowest-level outcomes were missing from the final selection.

Indicators that could be used to monitor each prioritized intervention were selected from the Catalog or defined by considering the intervention goal and the data available in the HR system. (The full list of potential indicators can be viewed in Annex 2 of the HR4SCM TOC Narrative.^23^)

### Data Processing and Analysis

We used manual thematic analysis to group inputs from interviews and focus groups to confirm the final results. Data from the close-ended interview questionnaire were analyzed in Excel to determine gaps across sites. Binary yes/no responses were entered into Excel for each site. Descriptive statistics were used to describe the binary interview data. TOC outcomes were classified as existing if interview responses or documentation collected indicated the outcome was present at the majority of sites. The results from site visits were validated through the focus group discussions. A manual thematic analysis of focus group data was conducted by EM as the primary researcher with review by co-researchers to determine priority, feasibility, and optimal implementation in the Rwanda context. A ranking score was generated for each outcome in Excel by assigning a numeric score to the categorical data.

### Ethical Approval

The researchers used a protocol approved by the Rwanda National Ethics Committee (IRB 00001497 of IORG0001100; FWA Assurance No 00001973). All study participants gave their written informed consent.

## RESULTS

### Participant Characteristics

Interviews were conducted with 35 staff, including HR managers, heads of facility and pharmacy staff, working at 20 sites. The sites visited included the central-level MOH and RMS Ltd (previously Rwanda Biomedical Centre-MPPD), 7 RMS regional warehouses (previously called district pharmacies), 5 district hospitals, 2 referral hospitals, and 4 health centers. The Kigali focus group included 7 participants from the central-level MOH, RMS Ltd, and each of the 3 sites visited. The Southern Province focus group convened 21 staff members from all 15 of the sites visited. Participants in the Southern Province focus group represented 6 RMS regional warehouses (district-level), 7 hospitals, and 2 health centers. Five participants worked in HR departments; the remaining 16 worked in pharmacy-related roles (Table 1).

**TABLE 1.** Characteristics of Participants, Rwanda

	**Kigali City, No. (%)**	**Southern Province, No. (%)**	**Total, No. (%)**
**Interviews**	(N=9)	(N=26)	(N=35)
Position type			
Human resources	3 (33)	6 (23)	9 (26)
Pharmacy	4 (44)	17 (54)	21 (60)
Other	2 (22)	3 (12)	5 (14)
Facility type			
Central level	6 (67)	0 (0)	6 (17)
District level	1 (11)	8 (31)	9 (26)
Hospital	1 (11)	12 (46)	13 (37)
Health center	1 (11)	6 (23)	7 (20)
**Focus group**	(N=7)	(N=21)	(N=28)
Position type			
Human resources	0 (0)	5 (71)	5 (54)
Pharmacy	5 (83)	15 (24)	20 (71)
Other	2 (33)	1 (5)	3 (11)
Facility type			
Central level	4 (57)	0 (0)	4 (14)
District level	1 (14)	6 (29)	7 (25)
Hospital	0 (0)	11 (52)	11 (39)
Health center	2 (29)	4 (19)	6 (22)

### Outcomes

The TOC model has 60 outcomes that contribute to optimal work performance.

As a result of the survey and workshop, participants determined that 12 outcomes were in place and 12 outcomes were not. Supplement 2 displays whether each outcome was determined to exist (Y/N) in the survey and workshop. Many of the 12 outcomes that were not in place were found higher in the causal pathway; thus, a related precondition lower in the pathway was first required. The workshop identified 32 lower-level outcomes that required data collection at site visits to determine their status, and these were included in the interview tool for the site visits.

During interviews conducted during the site visits, the statuses of the 32 lower-level outcomes were determined (Supplement 2). Based on site visit data, 14 more outcomes were deemed to be in place, 15 outcomes were determined to be absent, and 3 outcomes were partially in place. The top-level outcomes of each of the 4 pathways can be achieved only after the 56 lower-level outcomes of the pathway are developed. These 4 top-level outcomes were determined to be not in place, based on the logic of the TOC model, as 1 or more lower-level outcomes in each pathway were determined to be absent. Overall, our research identified that 26 outcomes were in place in Rwanda, 3 were partially in place, and 31 were deficient and required interventions to strengthen them (Table 2).

**TABLE 2.** Result for Each HR4SCM TOC Model Outcome in Rwanda Supply Chain

**#**	**Outcome**	**Result**
A4	Importance of SCM being acknowledged throughout health system & positioned accordingly.	Intervention suggested^a^
	**Staffing pathway**	
B1	All critical SCM positions and/or competencies filled.	Future phase^b^
B2.1	Ability to recruit quality candidates.	Future phase
B2.2	Adequate pool of workers to fill SCM roles/positions.	Future phase
B2.3	Sufficient budget to fund required positions.	Intervention suggested
B3.1	Ability to develop the right job descriptions.	Intervention suggested
B3.2	An effective recruitment system is in place for SCM positions.	In place^c^
B3.3	SCM workers have job security.	In place
B3.4	Competitive salaries are offered.	In place
B3.5	SCM job opportunities are known.	In place
B3.6	Education is available to obtain all required qualifications within the SCM system.	Intervention suggested
B3.7	SCM career path exists.	Intervention suggested
B3.8	Supply chain management is a valued career.	Intervention suggested
B4.1	Precise qualifications for SCM positions are accurately described.	In place
B4.2	General recruitment and hiring policy exists.	In place
B4.3	Equal employment opportunity policies cover recruitment practice.	Partially in place^d^
B5.1	Public sector recruitment and hiring policies permit the hiring of staff with adequate SCM experience.	In place
	**Skills pathway**	
C1	Workers apply their skills as appropriate at every level of the SCM.	Future phase
C2.1	SCM workers demonstrate adequate technical and managerial competencies.	Intervention suggested
C2.2	SCM workers have leadership skills within their sphere of operations.	Intervention suggested
C2.3	SCM workers understand their roles & responsibilities in the SCM system.	Future phase
C3.1	Workers have acquired adequate SCM competencies.	Future phase
C3.2	SCM workers develop competencies through coaching and mentoring.	In place
C3.3	SCM workers develop competence through learning and experience.	Future phase
C3.4	High-level SCM positions are recognized at a sufficient level of authority.	In place
C3.5	Formally defined roles match expected local practice.	In place
C3.6	Each position within SCM has defined roles and responsibilities.	Future phase
C4.1	SCM workers have access to training, education and professional development linked to core competencies.	Intervention suggested
C4.2	Opportunities exist to gain on-the-job experience.	In place
C4.3	The steps and competencies required to undertake SCM tasks are known.	Intervention suggested
	**Working conditions pathway**	
D1	Working conditions support performance.	Future phase
D2.1	The social and emotional environment is favorable.	Future phase
D2.2	The physical environment is safe, clean and conducive to performance.	Future phase
D2.3	SCM workers have up to date and relevant tools and equipment to perform.	Future phase
D3.1	A problem-solving, solution-focused culture exists.	In place
D3.2	The organization culture supports positive social and emotional environment.	In place
D3.3	Supervisors are competent to implement equal employment opportunity and anti-harassment policies.	Future phase
D3.4	Supervisors have the skills to establish a safe and clean physical work environment.	Future phase
D3.5	The resources necessary for safe, clean physical environment are available.	In place
D3.6	The necessary tools and equipment are identified and made available.	Intervention suggested
D4.1	Workplace harassment policies, especially those safeguarding women, are in place.	Partially in place
D4.2	Equal employment opportunity policies are in place.	Partially in place
D4.3	Environmental and occupational safety policies are in place.	Future phase
D5.1	The characteristics of a safe and conducive environment are known.	Intervention suggested
	**Motivation pathway**	
E1	SCM workers are motivated to do their jobs.	Future phase
E2.1	Good performance is supported within the system.	Future phase
E2.2	SCM workers understand and care about their role in the health care system.	Intervention suggested
E2.3	SCM workers have a sense of ownership over their role.	In place
E3.1	Poor performance is corrected.	In place
E3.2	Good performance is recognized and rewarded.	In place
E3.3	Good performance leads to career advancement.	In place
E3.4	There is an understanding of how SCM affects health outcomes.	In place
E3.5	Workers have the authority to make and implement decisions.	In place
E4.1	Financial incentives are in place.	In place
E4.2	Non-financial incentives are in place.	In place
E5.1	Supervisors provide supportive supervision and performance management to their staff.	In place
E6.1	Performance management policies are in place.	In place
E6.2	Supervisors understand the reasons for poor performance.	In place
E6.3	Supervisors feel able to provide constructive feedback.	In place
E6.4	Supervisors have the skills to communicate feedback on poor performance to staff.	Intervention suggested

Abbreviations: HR4SCM TOC; Human Resources for Supply Chain Management Theory of Change; SCM, supply chain management.

a Outcome was absent; intervention was suggested.

b Outcome was absent; intervention needs to be designed in a future phase, after the intervention suggested in this research develops a related precondition.

c Outcome was in place in the Rwandan system.

d Outcome partially in place; intervention was not suggested.

Our research identified that 26 outcomes were in place in Rwanda, 3 were partially in place, and 31 were deficient and required interventions to strengthen them.

Of the 31 deficient outcomes identified in this analysis, 14 were prioritized to be strengthened. As a result of focus groups and discussions with the MOH, 20 target interventions were selected to build these outcomes. The remaining 17 missing outcomes will require interventions in a future phase after related, lower-level preconditions are developed through the 20 interventions suggested by this analysis.

For the staffing pathway, results from the survey, workshop, and interviews indicated that 5 gaps were identified: insufficient budget existed to fund the required SCM positions, job descriptions at various levels did not specify SCM tasks, SCM was not seen as a valued career, education was not available for all skills SCM roles required, and a SCM career path did not exist. Table 3 shows 6 interventions suggested to develop 5 prioritized outcomes and recommended indicators.

**TABLE 3.** Suggested Interventions to Develop Deficient Outcomes in the HR4SCM TOC Staffing Pathway and Indicators

**Outcome**	**Suggested Intervention**	**Indicator**
Sufficient budget to fund required positions.	1.1 Forecast SCM positions. Include SCM positions in MOH staffing structure. 1.2 Allocate budget accordingly and support advocacy for SCM HR budget needs.	Evidence exists that vacant SCM positions are unfilled due to lack of funding (Y/N).
Ability to develop the right job descriptions.	1.3 Develop job descriptions for SCM functions at the different levels of the health system that meet a MOH-defined standard.	Percentage of SCM job descriptions that meet MOH’s standard for job descriptions.
SCM is a valued career.	1.4 Develop and establish a recognized supply chain cadre.	Percentage of SCM workers surveyed who consider SCM as a valued career.
Education is available for all required qualifications within the SCM system.	1.5a. Map the education available and the programs needed. 1.5b. Identify which programs are required based on competency frameworks; Identify which roles require pre-service training and at which level(s). 1.5c. Engage in advocacy with Ministry of Education to develop qualifications identified to be missing. 1.5d. Institute developed program in line with MOH needs.	Educational training is available at all levels for all qualifications staff require (Y/N). Training strategy that identifies educational requirements exists (Y/N). Number of employees who received SCM training at pre-service courses.
Supply chain management career path exists.	1.6a. Define a career path that maps low-level to upper-level experience. 1.6b. Align continuing professional development/education opportunities with career progression.	Percentage of managerial SCM roles that have existing career paths. Percentage of technical SCM roles that have existing career paths.

Abbreviations: HR4SCM TOC; Human Resources for Supply Chain Management Theory of Change; MOH, Ministry of Health; SCM, supply chain management.

Concerning the skills pathway, the data indicated that 4 gaps were identified: the competencies required for SCM tasks were not known, SCM workers did not demonstrate adequate technical and managerial competencies, SCM workers did not have access to training and professional development opportunities linked to core competencies, and SCM workers lacked leadership skills. Table 4 displays 7 interventions to develop these 4 prioritized outcomes and recommended indicators.

**TABLE 4.** Suggested Interventions to Develop Deficient Outcomes in the HR4SCM TOC Skills Pathway and Indicators

**Outcome**	**Suggested Intervention**	**Indicator**
The steps and competencies required to undertake SCM tasks are known.	2.1. Establish a competency framework and roles and responsibilities for SCM at all levels with corresponding SCM qualifications. 2.2. Conduct SCM competency mapping exercise.	Lists of critical SCM competencies have been documented for all SCM services (Y/N). Competency frameworks, which define the knowledge, skills, and attributes needed, are available for all SCM cadres (Y/N). All needed SCM competencies are assigned to SCM roles (Y/N).
SCM workers demonstrate adequate technical and managerial competencies.	2.3. Implement education and training interventions that address identified competency gaps. 2.4. Review job descriptions to ensure that appropriate qualifications are requested for the position.	Percentage of staff appraisals that assess SCM competencies.
SCM workers have access to training, education and professional development linked to core competencies.	2.5. Put in place staff development plans to support desired development.	Percentage of SCM staff with individual staff development plans.
SCM workers have leadership skills within their sphere of operations.	2.6. Incorporate leadership development into/as part of staff development plans. 2.7. Include a competency assessment in the performance evaluation process. Compare results of assessment with competencies listed in job description.	Percentage of staff who are competent in leadership competencies listed in their job description.

Abbreviations: HR4SCM TOC; Human Resources for Supply Chain Management Theory of Change; SCM, supply chain management.

In the working conditions pathway, the data indicated that 2 gaps were identified: a description of the characteristics of a safe and conducive environment did not exist, nor did a list of necessary tools and equipment for SCM workers. Table 5 displays 3 interventions suggested to develop these 2 outcomes.

**TABLE 5.** Suggested Interventions to Develop Deficient Outcomes in the HR4SCM TOC Working Conditions Pathway and Indicators

**Outcome**	**Suggested Intervention**	**Indicator**
The characteristics of a safe and conducive environment are known.	1.1. Consider policy development and list of required characteristics for safe and conducive environment with all personnel, implementing a checklist for confirmation. 1.2. Develop training materials on establishing safe and clean work environment.	List of required characteristics for a safe and conducive environment is accessible by all personnel (Y/N). Training materials on establishing a safe and clean work environment exist (Y/N).
The necessary tools and equipment are identified and made available.	1.3. Develop list of required tools and equipment for each level and share list/circular with all staff.	List of necessary tools and equipment for each level is accessible by all (Y/N).

Abbreviation: HR4SCM TOC; Human Resources for Supply Chain Management Theory of Change.

In the motivation pathway, results indicated that 2 gaps were identified: SCM workers did not understand their role in the health care system, and supervisors did not have the skills to communicate feedback on poor performance to staff. Table 6 details 2 interventions suggested to develop these 2 outcomes.

**TABLE 6.** Suggested Interventions to Develop Deficient Outcomes in the HR4SCM TOC Motivation Pathway and Indicators

**Outcome**	**Suggested Intervention**	**Indicator**
SCM workers understand and care about their role in the health care system.	4.1. Improve the staff onboarding and orientation processes, e.g., through a standard operating procedure for orientation.	Percentage of staff who feel they understand their role.
Supervisors have the skills to communicate feedback on poor performance to staff.	4.2. Develop and implement guidelines or procedures for supervisors on how to communicate feedback on poor performance.	Guidelines for supervisors that cover how to give constructive feedback are operational (Y/N).

Abbreviations: HR4SCM TOC; Human Resources for Supply Chain Management Theory of Change; SCM, supply chain management.

Focus group participants indicated that the foundational assumption of the TOC model was a key gap and that the importance of the SCM was not acknowledged throughout the health system and positioned accordingly in Rwanda. Table 7 shows 2 interventions to build the foundational assumption.

**TABLE 7.** Foundational Assumption of the HR4SCM TOC and Suggested Interventions and Indicators

**Outcome**	**Suggested Intervention**	**Indicator**
The importance of the SCM is acknowledged throughout the health system and positioned accordingly.	5.1. Develop orientation and on-boarding materials for clinical health staff deployed by MOH that include supply chain system and roles of staff in the SCM system. 5.2. Identify opportunities and communication channels for the organization to communicate to staff how their jobs contribute to the health care system.	Orientation materials for new health system staff include mention of supply chain or SCM roles (Y/N).

Abbreviations: HR4SCM TOC; Human Resources for Supply Chain Management Theory of Change; MOH, Ministry of Health; SCM, supply chain management.

## DISCUSSION

The factors that underlie workforce effectiveness in SCs, such as competencies and motivation, are usually complex, which makes these factors difficult to assess, communicate as needs to decision-makers, and improve. The lack of an appropriate analytical approach to assess the HR system can result in less effective, stand-alone interventions with impacts that are difficult to measure.^24^ Based on our experience in Rwanda, applying the HR4SCM TOC model is a comprehensive and time-efficient method for assessing gaps in the HR system and identifying interventions to address these gaps. Using this TOC model enabled a systematic approach to assess the HR system in Rwanda and select workforce interventions to improve HR4SCM broadly and within health SCs in Rwanda. Collecting data via a survey, a workshop, site visits, and focus groups enabled the researchers to check the presence of TOC outcomes from a variety of data sources, including staff interviews and document reviews. Each step of the data collection process was critical to identifying the weak outcome areas where interventions were most critically needed.

Applying the HR4SCM TOC model is a comprehensive and time-efficient method for assessing gaps in the HR system and identifying interventions to address these gaps.

Concerning the skills pathway, our analysis revealed that the steps and competencies required to undertake SCM tasks were not known and explicitly stated in the Rwandan system, and SCM workers lacked leadership skills. Further, our analysis found that in the staffing pathway, SCM was not seen as a valued career, a SCM career path did not exist, and education was not available for all required SCM qualifications.

To build complex, cross-cutting components missing from the skills and staffing pathways, Rwanda MOH is working with stakeholders to adapt the PtD SCM Professionalisation Framework (Supplement 3) in a coordinated, extensive project to address multiple gaps identified in this research.^26^ Through the process of adapting the PtD SCM Professionalisation Framework, Rwanda MOH will complete several interventions identified by this research, including establishing a competency framework and roles for SCM, completing an SCM competency mapping exercise, and identifying education and training needs to address identified competency gaps.^26^ Each of these interventions was identified through our approach (Table 4). The process will also ensure that job descriptions contain appropriate qualifications and that a competency assessment for performance evaluation is developed^26^—which were other interventions our analysis identified to improve the skills pathway (Table 4). In addition, adapting the PtD SCM Professionalisation Framework will map the needed educational programs, define career paths for SCM staff, and align continuing professional development/education opportunities with career progression^26^—interventions suggested to improve the staffing pathway (Table 3).

Our research revealed 2 key elements missing in the Rwanda HR system staffing pathway: the ability to recruit quality candidates and the existence of an adequate pool of workers to fill SCM roles. Although specific interventions were not prioritized as part of this research, the Rwanda MOH completed a labor market analysis to understand factors influencing the supply of skilled SCM workers and the demand for SCM labor across public and private sectors.^27^ The labor market analysis revealed skills gaps between the technical and managerial competencies that SCM workers demonstrate and the competencies that these positions require and concluded that actions were needed to increase both the supply of and demand for health SCM workers.^27^ Our research also highlighted the need for a process, such as the PtD SCM Professionalisation Framework, to stimulate the labor market for SCM workers. The Rwanda MOH considered the findings from this research together with the results from the labor market analysis in deciding to develop a local SCM professionalization framework for the SCM workforce in Rwanda.^26^ This work will help to ensure that an adequate pool of workers exists and that quality candidates can be recruited.

Our analysis revealed other gaps in the staffing pathway, including insufficient budget to fund the required SCM positions. In some cases, vacant SCM positions were unfilled due to lack of funding. In other cases, respondents felt that an adequate number of SCM positions were not forecasted and included in the MOH staffing structure. In addition, a review of job descriptions showed that SCM functions were often lacking and SCM tasks performed were not explicitly stated.

In the skills pathway, interviewees indicated that competency gaps existed in the technical and managerial competencies of SCM workers and that SCM workers did not have access to adequate training and professional development opportunities for certain required competencies.

Concerning the motivation pathway, interviewees indicated that SCM workers did not understand or appreciate their role within the broader health care system and that supervisors lacked sufficient skills to communicate feedback on poor performance to staff. Finally, respondents felt that the importance of the SCM was not acknowledged throughout the health system and positioned accordingly.

In the 3 years following the original data collection, the Rwanda MOH launched multiple initiatives to develop these missing HR4SCM TOC model outcomes. The findings from this research supported the MOH to prioritize and advocate for a number of changes. Under the staffing pathway, 2 SC-specific positions were created in the central MOH staffing structure, owing to interventions to forecast and include SCM positions in the MOH staffing structure. These additional positions still exist and are sufficiently funded (personal communication, A Kalema, March 2, 2022). In addition, SCM functions were specified and added to certain job descriptions to ensure the right job descriptions exist. The MOH launched 2 health SC e-learning modules for the in-service training of the existing health SCM workforce and onboarding of new staff to ensure SCM workers have access to training linked to core competencies.^15^^,^^28^ Under the motivation pathway, the e-learning modules and other training helped to improve staff onboarding and orientation processes for new SCM staff.^15^^,^^28^ The e-learning module and a leadership and change management course^29^ for key staff helped advance the intervention to develop guidelines for supervisors on communicating feedback on poor performance. As part of recognizing the importance of SCM professionals more broadly, the MOH’s Human Resources for Health Secretariat, which provides opportunities to strengthen the health workforce, now includes SCM professionals in their staff development efforts, in addition to clinical staff (personal communication, A Kalema, March 2, 2022). This analysis supported advocacy efforts to implement these changes.

The HR4SCM TOC model allowed the Rwanda MOH and stakeholders to acknowledge the distinct gaps in Rwanda’s system and showed stakeholders the detailed chain of preconditions required to improve those gaps. The model enhanced understanding among decision-makers about the complex factors that affect the performance of the health SCM workforce, such as competencies and factors influencing motivation. This shared understanding enhanced stakeholder engagement in the workshop and focus groups, and throughout the process to select interventions. Displaying the HR4SCM TOC model led stakeholders to appreciate the need for simple and more complex elements that were missing from the system and ultimately led to evidence-based decisions regarding workforce interventions. Assessing strengths and weaknesses across the 4 distinct pathways of the TOC model allowed stakeholders to identify how the recommended interventions could work synergistically within existing processes and with other new interventions to improve performance and increase the availability of health products and services.

Despite progress in completing some of the interventions suggested by our research, the recommended indicators have not been used systematically to monitor progress in implementing the selected interventions. This is largely due to competing priorities. In addition, many of the suggested indicators are binary (Y/N) indicators that track whether large, time-intensive interventions have been completed. As these indicators are not sufficiently sensitive to monitor small changes in performance and many interventions are still in progress, the benefit of actively monitoring this subset of indicators might not have been recognized. Further, the remaining indicators track percentages of staff and rely on data sources (e.g., staff surveys) that require new data collection processes. The MOH currently does not have a process to monitor SCM HR system performance.

### Limitations

During this analysis, researchers worked primarily with 1 MOH department (Department of Clinical and Public Health Services) for the survey, workshop, and site visit data collection. However, the HR interventions are cross-cutting, and their implementation requires cooperation across various MOH departments and educational institutions. In Rwanda MOH, this includes the directorates of (1) Human Resources; (2) Planning, Health Financing, and Information Systems; and (3) Clinical and Public Health Services. This research would have benefited from a broader inclusion of MOH departments in data collection. Difficulty in engaging all 3 departments at the MOH simultaneously throughout the research process limited greater input. Although members of each department were interviewed, the Department of Clinical and Public Health Services led the intervention development and baseline data collection. Engaging a cross-cutting steering committee with leadership across all relevant departments is key to developing appropriate, achievable interventions and would benefit future applications of this tool.

The survey and workshop were limited to a small number of staff within 1 MOH department, and data collection was limited to a small number of staff and sites. The status of the TOC outcomes might have been different at other sites; however, as data collection focused on HR policies, approaches, and processes that typically were governed by a whole MOH approach, we expected general similarities to exist across other sites. Critical elements might be missing from the HR4SCM TOC model that could lead to gaps in the areas covered by the interventions suggested by this model. Finally, our analysis considered HR elements of SC performance, and for health products to be available, the SC system will also require other elements outside of HR to be strengthened, such as policies and procedures, infrastructure, data flows, and finance.

## CONCLUSIONS

SCM workforce performance is a key enabler of SC performance and increased access to health commodities. The HR4SCM TOC model enables users to assess how a country’s existing HR system compares to the conditions necessary for optimized SCM workforce performance. Evaluating the presence of this TOC model’s outcomes across 4 distinct pathways enabled a comprehensive, systematic assessment of weaknesses in the SCM HR management system in Rwanda. Identifying gaps between the optimal TOC model conditions and the Rwandan SC system provided an evidenced-based path to select interventions. Applying the model as a standardized tool enabled the effective assessment of the SCM HR management system with limited preparation and in-country time, as well as minimal training of the local team. The tool was suitable to be applied to all levels of the SC system. This research assessed HR gaps, identified required interventions, and defined measures for performance and progress. This approach enabled the Rwandan MOH to prioritize its HR SCM investments with an aim to improve the availability of the skilled cadres required for the ongoing effective management of health SCs in Rwanda.

The HR4SCM TOC model is a valuable tool that allows governments, donors, and technical partners to articulate and appreciate the complexities that govern HR in health SCs and to understand how interventions can navigate this complex environment to create change. Applying this TOC model will help practitioners follow a structured approach to developing an optimal SC workforce by assessing gaps in hard-to-measure areas (e.g., capacity-strengthening and institutional development) and designing appropriate interventions. By discussing the desired outcomes, planners can decide how to apply skills, knowledge, and resources to fulfill each outcome. National stakeholders and program managers should consider assessing their SCM HR system against the HR4SCM TOC model, which provides a systematic approach to identify gaps and an evidenced-based approach to design and prioritize interventions.

## Supplementary Material

GHSP-D-23-00062-Meier-Supplement2.pdf

GHSP-D-23-00062-Meier-Supplement3.pdf

GHSP-D-23-00062-Meier-Supplement1.pdf

## Notes

**Peer Reviewed**

**First Published Online:** October 23, 2024.
