# Supplementary material for: Applying a Theory of Change for Human Resources Development in Public Health Supply Chains in Rwanda
Source: Glob Health Sci Pract. 2025 May 9;13(Suppl 1):e2300062. doi: 10.9745/GHSP-D-23-00062 (PMC12063746; doi:10.9745/GHSP-D-23-00062)
Supplement: GHSP-D-23-00062-Meier-Supplement2.pdf [file GHSP-D-23-00062-Meier-Supplement2.pdf]

Supplement to: Meier E, Brown AN, McHenry B, Kabatende J, Gege Buki IK, Icyimpaye J. Applying a theory of change for human resources development in public health supply chains in Rwanda. *Glob Health Sci Pract*. 2024;12(Suppl 3):e2300062. https://doi.org/10.9745/GHSP-D-23-00062

Supplement 2. Summary of Results for each Methodological Step

#	HR4SCM ToC Outcome	1. Survey	2. Workshop	3. Interviews	Result	Phase Determined
A4	Importance of SCM being acknowledged throughout health system & positioned accordingly	N/A*	N/A*	N	Intervention Suggested	Focus Groups
	**Staffing pathway**					
B1	All critical SCM positions and/or competencies filled	N/A*	N/A*	N/A*	Future Phase	*Staffing Pathway top-level outcome*
B2.1	Ability to recruit quality candidates	N	N	N/A	Future Phase	Workshop
B2.2	Adequate pool of workers to fill SCM roles/positions	N	N	N	Future Phase	Interviews
B2.3	Sufficient budget to fund required positions	N	N	N	Intervention Suggested	Interviews
B3.1	Ability to develop the right job descriptions	N	N	N	Intervention Suggested	Interviews
B3.2	An effective recruitment system is in place for SCM positions	N	N	Y	In Place	Interviews
B3.3	SCM workers have job security	Y	Y	N/A	In Place	Workshop
B3.4	Competitive salaries are offered	Y	Y	N/A	In Place	Workshop
B3.5	SCM job opportunities are known	N	N	Y	In Place	Interviews
B3.6	Education is available to obtain all required qualifications within the SCM system	N	N	N	Intervention Suggested	Interviews
B3.7	SCM career path exists	N	N	N	Intervention Suggested	Interviews
B3.8	Supply chain management is a valued career	Y**	N/A	N	Intervention Suggested	Interviews
B4.1	Precise qualifications for SCM positions are accurately described	N	N	Y	In Place	Interviews
B4.2	General recruitment and hiring policy exists	Y	Y	Y	In Place	Workshop
B4.3	Equal employment opportunity policies cover recruitment practice	N	N	N	Partially in Place	Interviews
B5.1	Public sector recruitment and hiring policies permit the hiring of staff with adequate SCM experience	Y	Y	N/A	In Place	Workshop
	**Skills pathway**					
C1	Workers apply their skills as appropriate at every level of the SCM	N/A*	N/A*	N/A*	Future Phase	*Skills Pathway Top-level outcome*
C2.1	SCM workers demonstrate adequate technical and managerial competencies	N	N	N	Intervention Suggested	Interviews
C2.2	SCM workers have leadership skills within their sphere of operations	N	N	N/A	Intervention Suggested	Workshop

Supplement to: Meier E, Brown AN, McHenry B, Kabatende J, Gege Buki IK, Icyimpaye J. Applying a theory of change for human resources development in public health supply chains in Rwanda. *Glob Health Sci Pract*. 2024;12(Suppl 3):e2300062. https://doi.org/10.9745/GHSP-D-23-00062

#	HR4SCM ToC Outcome	1. Survey	2. Workshop	3. Interviews	Result	Phase Determined
C2.3	SCM workers understand their roles & responsibilities in the SCM system	N	N	N/A	Future Phase	Workshop
C3.1	Workers have acquired adequate SCM competencies	N	N	N/A	Future Phase	Workshop
C3.2	SCM workers develop competencies through coaching and mentoring	Y	N/A	N/A	In Place	Survey
C3.3	SCM workers develop competence through learning and experience	N	N	N/A	Future Phase	Workshop
C3.4	High-level SCM positions are recognized at a sufficient level of authority	Y	N/A	N/A	In Place	Survey
C3.5	Formally defined roles match expected local practice	N	N	Y	In Place	Interviews
C3.6	Each position within SCM has defined roles and responsibilities	N	N	N	Future Phase	Interviews
C4.1	SCM workers have access to training, education and professional development linked to core competencies	N	N	N	Intervention Suggested	Interviews
C4.2	Opportunities exist to gain on-the-job experience	N	Y	Y	In Place	Interviews
C4.3	The steps and competencies required to undertake SCM tasks are known	N	N	N	Intervention Suggested	Interviews
	**Working conditions pathway**					
D1	Working conditions support performance	N/A*	N/A*	N/A*	Future Phase	*Working Conditions Top-level outcome*
D2.1	The social and emotional environment is favorable	N	N	N/A	Future Phase	Workshop
D2.2	The physical environment is safe, clean and conducive to performance	N	N	N/A	Future Phase	Workshop
D2.3	SCM workers have up to date and relevant tools and equipment to perform	N	N	N/A	Future Phase	Workshop
D3.1	A problem-solving, solution-focused culture exists	Y	N/A	N/A	In Place	Survey
D3.2	The organization culture supports positive social and emotional environment	Y	N/A	N/A	In Place	Survey
D3.3	Supervisors are competent to implement EEO and anti- harassment policies	N	N	N/A	Future Phase	Workshop
D3.4	Supervisors have the skills to establish a safe and clean physical work environment	N	N	N/A	Future Phase	Workshop

Supplement to: Meier E, Brown AN, McHenry B, Kabatende J, Gege Buki IK, Icyimpaye J. Applying a theory of change for human resources development in public health supply chains in Rwanda. *Glob Health Sci Pract*. 2024;12(Suppl 3):e2300062. https://doi.org/10.9745/GHSP-D-23-00062

#	HR4SCM ToC Outcome	1. Survey	2. Workshop	3. Interviews	Result	Phase Determined
D3.5	The resources necessary for safe, clean physical environment are available	N	N	Y	In Place	Interviews
D3.6	The necessary tools and equipment are identified and made available	N	N	N	Intervention Suggested	Interviews
D4.1	Workplace harassment policies, especially those safeguarding women, are in place.	N	N	N	Partially in Place	Interviews
D4.2	Equal employment opportunity (EEO) policies are in place	Y**	N	N	Partially in Place	Interviews
D4.3	Environmental and occupational safety policies are in place	N	N	N	Future Phase	Interviews
D5.1	The characteristics of a safe and conducive environment are known	N	N	N	Intervention Suggested	Interviews
	**Motivation pathway**					
E1	SCM workers are motivated to do their jobs	N/A*	N/A*	N/A*	Future Phase	*Motivation Pathway Top-level outcome*
E2.1	Good performance is supported within the system	N	N	N/A	Future Phase	Workshop
E2.2	SCM workers understand and care about their role in the healthcare system	N	N	N	Intervention Suggested	Interviews
E2.3	SCM workers have a sense of ownership over their role	N	N	Y	In Place	Interviews
E3.1	Poor performance is corrected	N/A	N	Y	In Place	Interviews
E3.2	Good performance is recognized and rewarded	N/A	N	Y	In Place	Interviews
E3.3	Good performance leads to career advancement	N/A	Y	N/A	In Place	Workshop
E3.4	There is an understanding of how SCM affects health outcomes	N/A	Y	N/A	In Place	Workshop
E3.5	Workers have the authority to make and implement decisions	N/A	N	Y	In Place	Interviews
E4.1	Financial incentives are in place	N/A	N	Y	In Place	Interviews
E4.2	Non-financial incentives are in place	N/A	N	Y	In Place	Interviews
E5.1	Supervisors provide supportive supervision and performance management to their staff	N/A	N	Y	In Place	Interviews
E6.1	Performance management policies are in place	N/A	Y	Y	In Place	Interviews
E6.2	Supervisors understand the reasons for poor performance	N/A	Y	Y	In Place	Workshop
E6.3	Supervisors feel able to provide constructive feedback	N/A	Y	Y	In Place	Workshop

Supplement to: Meier E, Brown AN, McHenry B, Kabatende J, Gege Buki IK, Icyimpaye J. Applying a theory of change for human resources development in public health supply chains in Rwanda. *Glob Health Sci Pract*. 2024;12(Suppl 3):e2300062. https://doi.org/10.9745/GHSP-D-23-00062

#	HR4SCM ToC Outcome	1. Survey	2. Workshop	3. Interviews	Result	Phase Determined
E6.4	Supervisors have the skills to communicate feedback on poor performance to staff	N/A	N	N	Intervention Suggested	Workshop

Table Legend:	
	HR4SCM ToC outcome was in place in the Rwandan system
	HR4SCM ToC outcome was partially in place; intervention was not suggested
	HR4SCM ToC outcome was absent; intervention was suggested
	HR4SCM ToC outcome was absent; intervention needs to be designed in a future phase, after the intervention suggested in this research develops a related precondition

*Item was not included in the specified step because this item depends on a related, lower-level outcome under assessment in that step.

**Indicates survey results that changed during subsequent data collection steps.

Note: 12 outcomes in the motivation pathway (E3.1-E6.4) were not included in the survey due to an error during data collection and are marked N/A for this reason.
