# Supplementary material for: Applying a Theory of Change for Human Resources Development in Public Health Supply Chains in Rwanda
Source: Glob Health Sci Pract. 2025 May 9;13(Suppl 1):e2300062. doi: 10.9745/GHSP-D-23-00062 (PMC12063746; doi:10.9745/GHSP-D-23-00062)
Supplement: GHSP-D-23-00062-Meier-Supplement3.pdf [file GHSP-D-23-00062-Meier-Supplement3.pdf]

SCM Professionalisation Framework

— LIBRARY OF COMPETENCIES & DESIGNATIONS FOR HEALTH SUPPLY CHAINS;
— COLLECTION OF ROLES AND JOB DESCRIPTIONS FOR HEALTH SUPPLY CHAINS;
— MAPPING OF EDUCATION FOR HEALTH SUPPLY CHAINS;
— IMPLEMENTATION APPROACH FOR HEALTH SUPPLY CHAINS.

NOVEMBER 2020

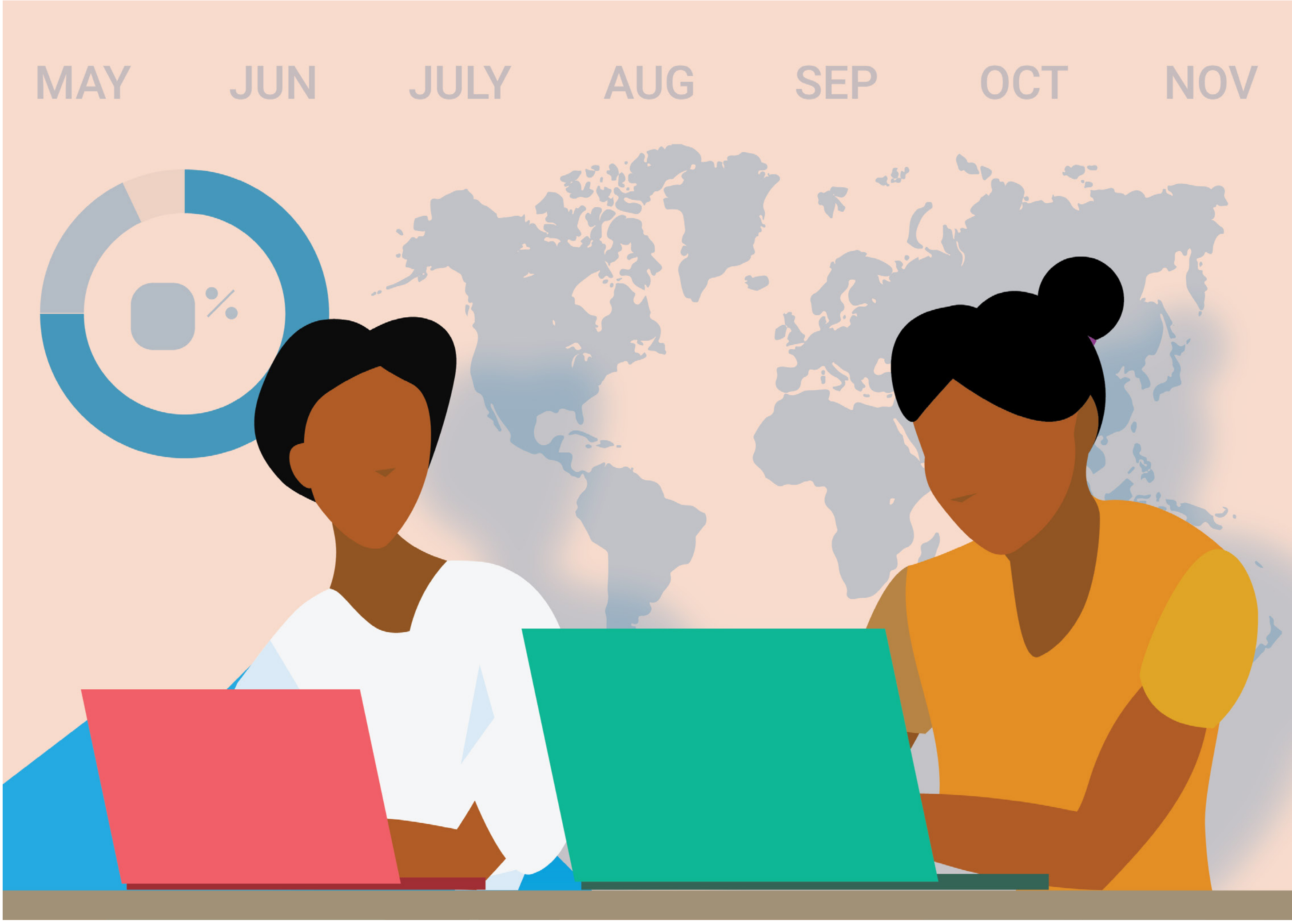

PEOPLE THAT DELIVER

SCM Professionalisation Framework

— LIBRARY OF COMPETENCIES & DESIGNATIONS FOR HEALTH SUPPLY CHAINS
— COLLECTION OF ROLES AND JOB DESCRIPTIONS FOR HEALTH SUPPLY CHAINS
— MAPPING OF EDUCATION FOR HEALTH SUPPLY CHAINS
— IMPLEMENTATION APPROACH FOR HEALTH SUPPLY CHAINS

NOVEMBER 2020

PEOPLE THAT DELIVER

Authors

Andrew dos Santos
MPhil, BSc (Hons)., SCOR-P, CPIMDirector, CLX

Pretoria, South Africa.
andrew@clx.co.za

Richard dos Santos
BBA, CPIM, CSCP, Director, SAPICS

Copenhagen, Denmark,
richard@sapics.org.za

Bridget McHenry
MPH, CSCP, Senior Organisational Development Advisor

Commodities Security & Logistics Division, Bureau for Global Health | Office of Population & Reproductive Health USAID

Arlington, Virginia, USA.
bmchenry@usaid.gov

Michael Egharevba
BPharm MBA, MPH, PGDSCM, PGDDA, SPSM, SPSM2

Workforce Development Specialist

USAID Global Health Supply Chain-Procurement Supply Management (GHSC-PSM) Project

Arlington, Virginia, USA.
megharevba@ghsc-psm.org

Dr Andrew Brown
PhD, BPharm, GCHE

Senior Director Health Workforce Development (PtD Coalition Member)

IntraHealth, Chapel Hill, North Carolina, USA.
abrown@intrahealth.org

Dominique Zwinkels
Executive Manager, People that Deliver (PtD)

Copenhagen, Denmark
dzwinkels@unicef.org

Barry Chovitz
USAID Global Health Supply Chain-Procurement Supply Management (GHSC-PSM) Project

bchovitz@ghsc-psm.org

James Johnson
USAID Global Health Supply Chain-Procurement Supply Management (GHSC-PSM) Project

jajohnson@ghsc-psm.org

Acknowledgements

This work was funded by the USAID Global Health bureau's Family Planning and Reproductive Health (FP/RH) programme. This work was a joint activity of the USAID Global Health Supply Chain Programme-Procurement and Supply Management (GHSC-PSM) project, People that Deliver (PtD), SAPICS, and USAID.

The authors would like to thank the members of these organisations for their contribution and cooperation as together we seek to increase the availability of medicines to beneficiaries. Many colleagues gave their time freely for interviews and to engage in focus group discussions; we thank you for your contribution.

The SCM Professionalisation Framework is dedicated to Andrew dos Santos. His hard work and knowledge were central to the development of this resource.

DISCLAIMER
The authors' views expressed in this publication do not necessarily reflect the views of the U.S. Agency for International Development or the U.S. government.

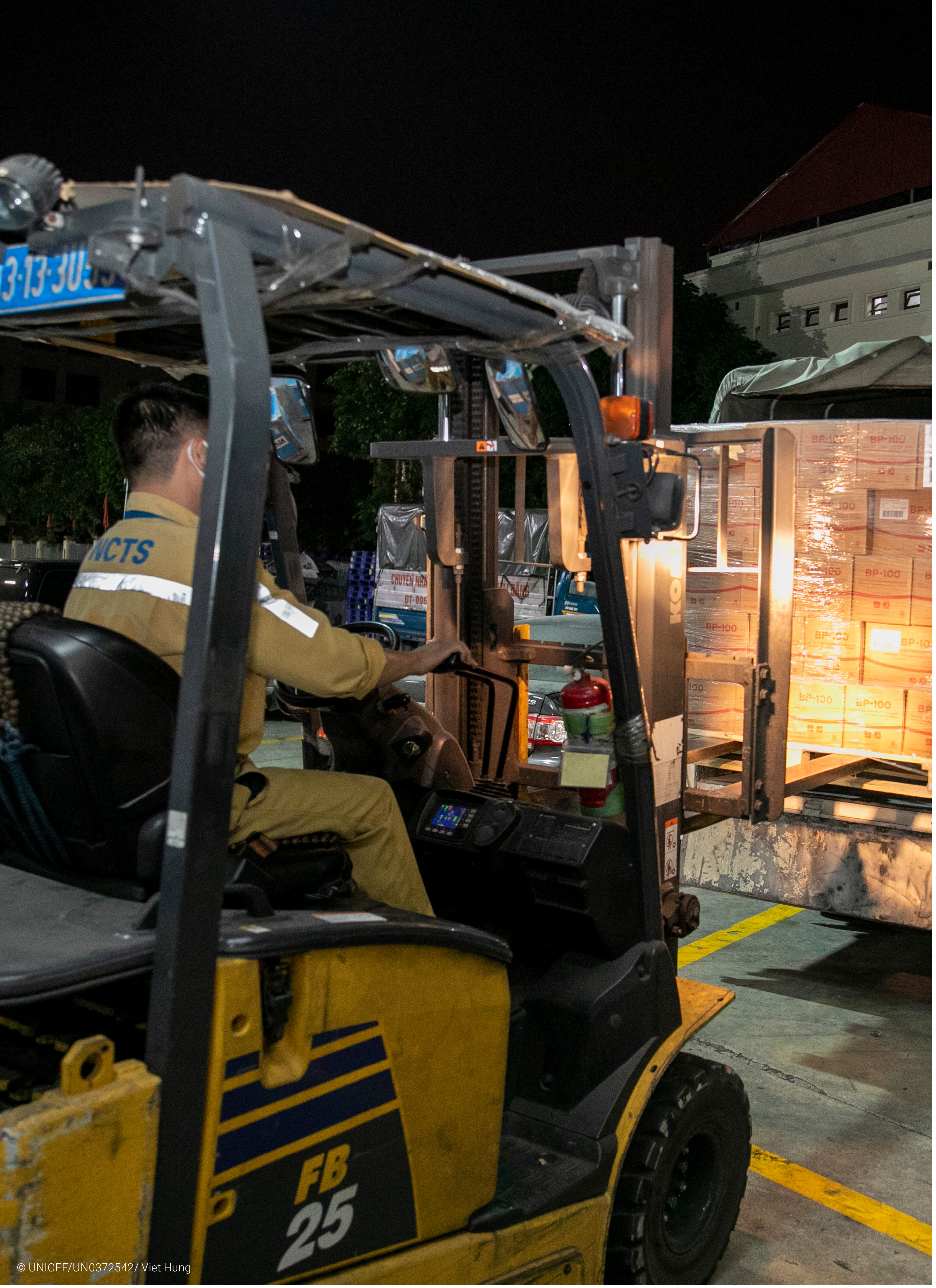

© UNICEF/UN0372542/ Viet Hung

Contents

Authors 4

Acknowledgements 5

Overview 8

Library of Competencies & Designations for Health Supply Chains 11

- Competency terminology and definitions 11
- Methodology 14

Collection of Roles and Job Descriptions for Health Supply Chains (Demand) 17

- Methodology 18

Mapping of Education for Health Supply Chains 22

- Methodology 23

Appendices 26

Appendix A: Organisation participation or past study organisations 27

Appendix B: Library of Competencies & Designations for Health Supply Chains 28

Appendix C: SAQA structure 95

List of Abbreviations

ABBREVIATION	MEANING
GHSC-PSM	USAID Global Health Supply Chain Programme-Procurement and Supply Management
HSCM	Health Supply Chain Management
JD	Job Descriptions
NLP	Natural Language Processing
PtD	People that Deliver
SAPICS	Industry Association for Supply Chain Management in South Africa
SAQA	South African Qualifications Authority
SC	Supply chain
SCM	Supply chain management

Overview

To better understand issues in the availability and use of supply chain management (SCM) human resources in a country context, the USAID Global Health Supply Chain Programme-Procurement and Supply Management (GHSC-PSM) project, in conjunction with USAID and People that Deliver (PtD), considers that a "whole of SCM labour market" approach provides a deeper and more holistic understanding of the SCM employment environment.

The whole of the SCM labour market refers to the supply and demand for SCM labour in which employees are the supply and employers the demand in a specific country context. The country context includes urban, regional, and more remote environments and encompasses all the sectors where SCM technical personnel are employed in that country.

Key stakeholders include government (ministries of labour, education, planning, and health, etc.), professional associations, academic institutions, private sector (resources industries, fast-moving goods, health, third-party logistics providers (3PL) and fourth-party logistics providers (4PL), etc.), and the humanitarian and development sectors

In 2019 GHSC-PSM, in collaboration with PtD, SAPICS and USAID, published a SCM Professionalisation Framework white paper outlining how a SCM professionalisation framework could be used by:

- Governments to define the professional standards of the profession
- Employers to articulate SCM competency requirements and career pathways in their organisations
- Learning institutions of to define clear learning and teaching courses
- SCM employees to map out a professional career in SCM

In 2020, the same consortium worked together to complete the necessary elements of the 'SCM Professionalisation Framework'. This framework has a ***'Library of Competencies & Designations for Health Supply Chains'*** as its core meeting public and private sector needs. The competency framework then acts as the 'standard' to ensure an aligned ***'Mapping of Education for Health Supply Chains'*** (supply) and ***'Collection of Roles and Job Descriptions for Health Supply Chains'*** (demand), for a particular country context. Further, the ***'Implementation Approach for Health Supply Chains'*** provides clear guidance on how to begin this journey of change. Figure 1 shows the interrelationship of these elements.

Although each of the SCM Professionalisation Framework components can be used by itself it is believed that most benefit is obtained from using the ***'Implementation Approach for Health Supply Chains'*** to create lasting systems change.

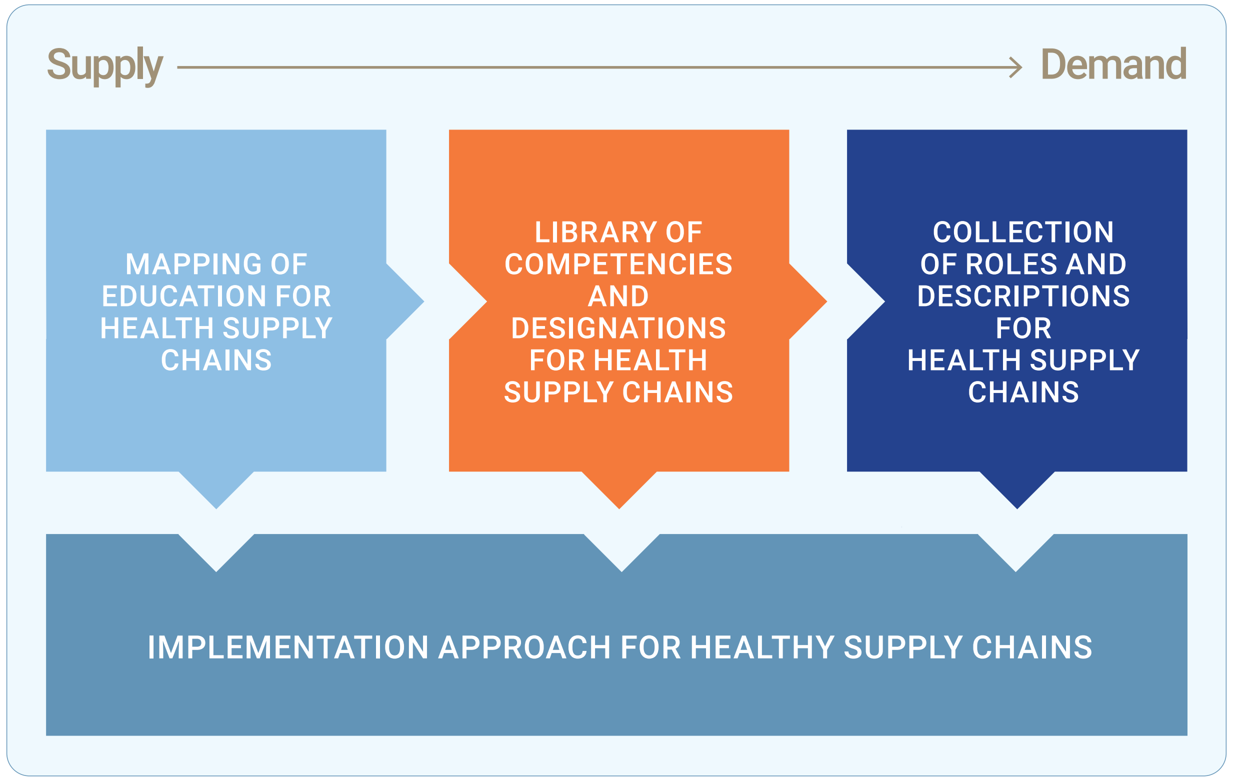

Figure 1:
Elements of the PtD Professionalisation Competency Framework

Library of Competencies & Designations for Health Supply Chains

Building on the PtD Health Supply Chain Management (HSCM) Competency Compendium (2014), this serviced-based (non-cadre specific) framework presents seven competency domains that cover the practice of HSCM across the public and private sector (Figure 1).This comprehensive framework provides 'behaviour statements' outlining workplace expectation in all listed competency domains and competency groups. This revised version provides a five-level maturity or designation classification for each competency area, acting as a 'standard' for comparing education requirements and the related job descriptions.

Collection of Roles and Job Descriptions for Health Supply Chains (Demand)

Building on previous work conducted by PtD (2018), this tool provides a systematic way to build job descriptions and consider SCM roles with reference to the Library of Competencies & Designations for Health Supply Chains. An example set of SCM job descriptions and organisational charts are provided.

Mapping of Education for Health Supply Chains (Supply)

This tool provides an explanation of how an education and training framework should be considered within a country context, to align with SCM job roles. The tool also uses a machine learning approach to review existing SCM education and training opportunities to determine alignment with domains and levels within the Library of Competencies & Designations for Health Supply Chains.

Implementation Approach for Health Supply Chains

This tool provides an overview of the step by step activities that can be undertaken over a three-week period to engage local stakeholders advocating the need to implement a SCM Professionalisation approach. This methodology also validates a plan of action to apply the Library of Competencies & Designations for Health Supply Chains, Collection of Roles and Job Descriptions for Health Supply Chains and Mapping of Education for Health Supply Chains to systematically improve SCM professionalisation in the country context.

Library of Competencies & Designations for Health Supply Chains

Competency terminology and definitions

Internationally, the use of competency-related terminology can vary, so the following definitions are offered here to encourage common understanding:

Competency compendium: A comprehensive catalogue of competency areas with the associated behavioural competencies referencing common supply chain processes and job level activities.

Domains: The high-level groups, or clusters of competency areas, within the compendium. Traditionally, domains do not exceed six. The six domains used in this PtD competency compendium are selection and quantification, procurement, storage and distribution, use, resource management, and professional and personal (Figure 3).

Competency area: The overarching capacity/skills of a person to perform in a specific area. For example, within the domain of procurement, PtD has suggested the following competency areas:

2.1 Manage procurement costs and budget
2.2 Build and maintain supplier relationships
2.3 Manage tendering processes and supplier agreements
2.4 Execute management of contract, including risk and quality management
2.5 Assure quality of products
2.6 Manage import and export of products
2.7 Manage donations of products

Behavioural competencies

Expressions of the work activities performed and observed when professionals apply motives, traits and skills to a relevant task.

For example, for the domain of procurement and the competency area of manage tendering processes and supplier agreements, such behavioural competencies may include “develop bidding documents”, “use WHO prequalification system to confirm quality suppliers” , “manage a tender process using country systems” , or “formalise contracts with successful companies.” This compendium contains behavioural competencies and references to required knowledge, depending on the source document used.

Competency framework

A collection of competency areas with associated behavioural competencies that define the expected requirements of a particular cadre/profession (Figure 2).

For example, a competency framework may be developed for a warehouse manager, while a separate competency framework would be required for a pharmacist. The composition of country-based, cadre-specific competency frameworks will also depend on the structure of the supply chain and at which levels various competencies are allocated.

Designation levels

Accommodating the differing levels of work focus and scope as well as denoting training and education required at each level. Each designation level has been aligned to ensure articulation between levels or elimination of competency overlap. The competency framework is divided into designation levels namely:

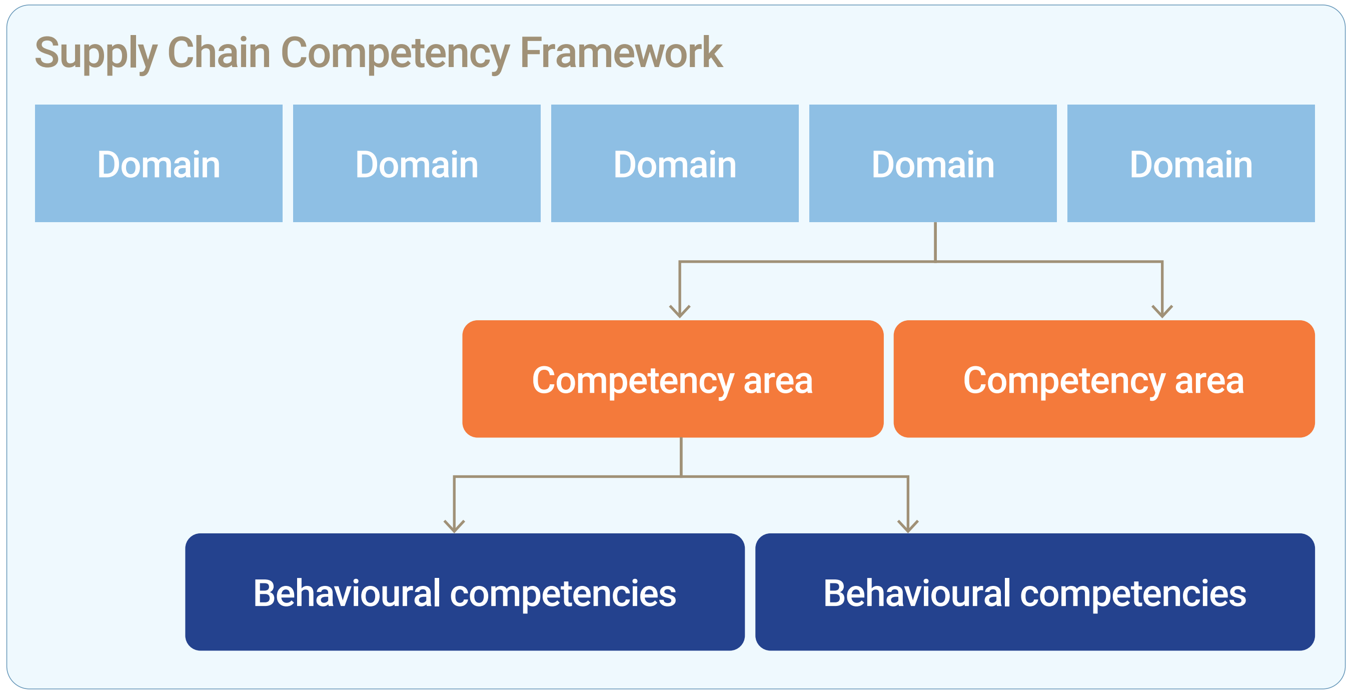

Figure 2:
The structure of competency frameworks

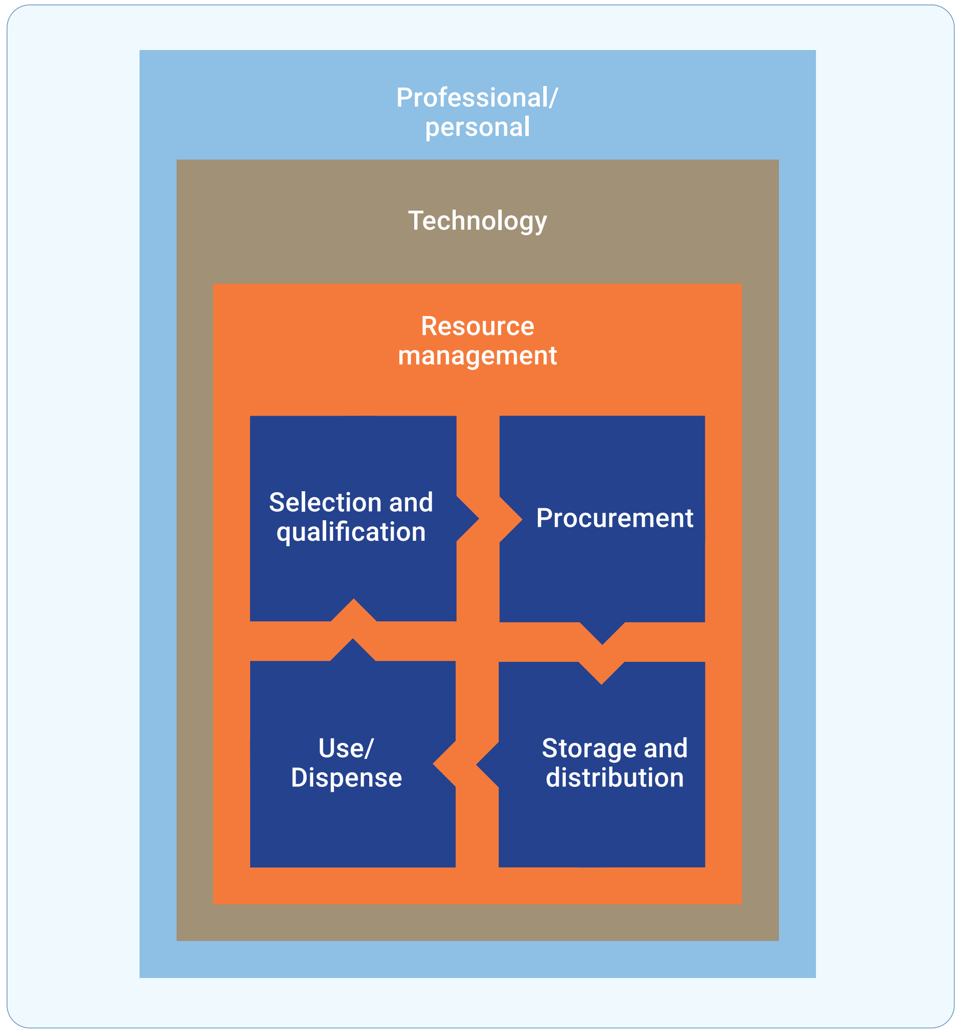

Figure 3:
The relationship of the six domains within the PtD Competency Compendium for Health Supply Chain Management

Associate

Associate level is equivalent to entry level in the competency framework. This is an execution level designation.

Practitioner

Practitioner level is the first management level in the competency framework. This is an execution level designation with some supervisory and management competencies.

Specialist

Specialist level is the mid-management level designation. Typically associated with management level accountability depending on domain.

Professional

Professional level is the first strategic level designation and is typically characterised by analysis and input into strategic decision making.

Leader

Leader level is the primary strategic level designation and is characterized by long-term decision-making competencies.

Each designation level has a set of associated terms that denote the level and nature of a particular competence an individual must possess in order in order to fulfil the workplace duties associated with a specific activity.

For example, in the Storage Domain the behavioural competency:

Ensure accurate verification of rolling stocks, the Associate designation level is required to have an "awareness of the importance of accurate verification of rolling stocks" while the Practitioner needs to "Understand the importance of accurate verification of rolling stocks".

In this case the difference is indicated by the terms Awareness and Understand. There are several terms used across the competency that have been referenced from multiple educational and vocational resources.

As displayed in the excerpt from the competency framework below:

Methodology

The very nature of a professional body is that it encapsulates a path to professionalisation. For this reason the PtD Competency Compendium for Health Supply Chain Management is an ideal base for the Library of Competencies & Designations for Health Supply Chains. In order to encapsulate a full pathway, however, the competencies needed to be expanded in responsibility and complexity. Additionally, PtD had already extensively reviewed the document and researched additions to the framework. These additions were added before the expansion of the competencies and then developed in the same manner as the rest of the framework.

In addition to this expansion, and based on the previous review, the authors conducted an extensive review, comparing the Professionalisation Framework to current best practice supply chain process frameworks in order to verify its validity in the broader sense of supply chain management. Where necessary wording was expanded, however this analysis was used more to develop the framework into the higher and lower levels as required. In order to develop these levels the authors had to first define how many levels would be needed. For this there were a few inputs: the first was the structure of other supply chain professional body frameworks and the second was job descriptions and hierarchies from private and public organisations either generously donated or from previous projects, which would both allow the framework to be compatible with the majority of hierarchies as well as other professional bodies in the sample set (Appendix A). From these inputs it was determined that five levels would be required to capture the complexity exhibited by these documents bearing in mind that the levels are in themselves only indicative and can be edited by countries in their own implementations.

In order to expand the PtD Competency Compendium for Health Supply Chain Management into those levels it was necessary to use a trusted framework to expand the responsibility and complexity requirements of each competency. In order to keep a standard toward this, blooms taxonomy's cognitive and affective domains are used to increase complexity of the competence and the previous analysis using supply chain frameworks is used to increase responsibility as domains increase in level using the appropriate terms associated with the domain aligned to the designation. The final domains chosen are named accordingly and are aligned to blooms taxonomy:

- Associate
- Practitioner
- Specialist
- Professional
- Leader

Table 1: Alignment of Blooms Taxonomy to Professional Designations

Knowledge	***Comprehension***	***Application***	***Analysis***	***Evaluation***	***Synthesis***
Associate	Associate	Practitioner	Specialist	Professional	Leader

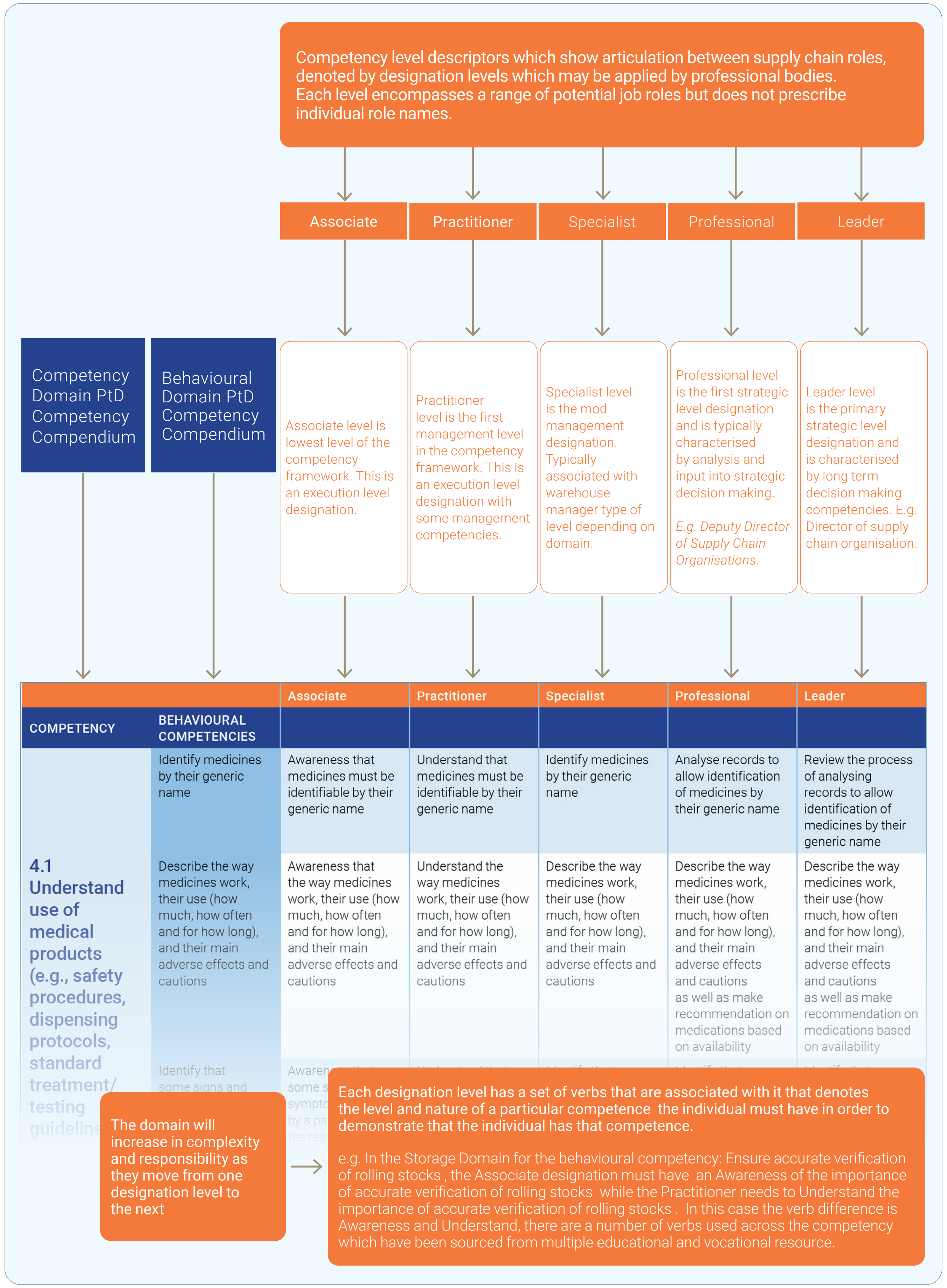

Figure 4:
Description of how the Library of Competencies & Designations for Health Supply Chains is constructed

Collection of Roles and Job Descriptions for Health Supply Chains (Demand)

The SCM Professionalisation Framework aims to professionalise supply chain management personnel and should have the effect of streamlining multiple aspects of supply chain management in a country or organisation. However, the country or organisation must need these personnel; in other words a demand must be created. The Collection of Roles and Job Descriptions for Health Supply Chains was created for this purpose: it outlines ways in which the Library of Competencies & Designations for Health Supply Chains can be organised operationally to create this demand.

There are several ways that this job description (JD) compendium can assist you, whether you are involved in operations management or workforce development.

This compendium was designed to be a reference guide for and to assist human resource and supply chain practitioners in the field in designing their organisational hierarchies and planning for human resource initiatives across the healthcare supply chain.

Whilst the job description templates are provided as a point of reference for workforce development activities, they are best utilised with the associated PtD Professionalisation Approach, which contains details pertaining to whole of labour market considerations where these templates are used as a component in creating a tailored workforce development plan.

Methodology

The Collection of Roles and Job Descriptions for Health Supply Chains (Demand) was created with primary input from previous work done within PtD, which received input from multiple country examples to create the base job descriptions. These base job descriptions were then compared with a set of hierarchies that encompass the majority of roles, as below, to create the hierarchy found in this document. Based on aggregated descriptions of the job descriptions studied, the base job descriptions were assigned a primary domain and primary competencies. It must be stressed, however, that the example in this document is only one of many ways to combine the job descriptions.

Once the base job descriptions were created, the metrics and training needed to be defined. In order to define the training and metrics, the domains in which the job descriptions reside and the primary competencies were used to align the job descriptions to various supply chain management frameworks. These define standard practices and metrics for the activities to which the job description pertains, and these alignments were then used to assign standard metrics to the job descriptions.

Finally using the competencies assigned to each job description, the Mapping of Education for Health Supply Chains was used to fill in possible education, which stipulates the competencies required for an individual to take up each job description.

To fully utilise the compendium, one must look at the attributes of a job description (JD), which are structured using the following table headers:

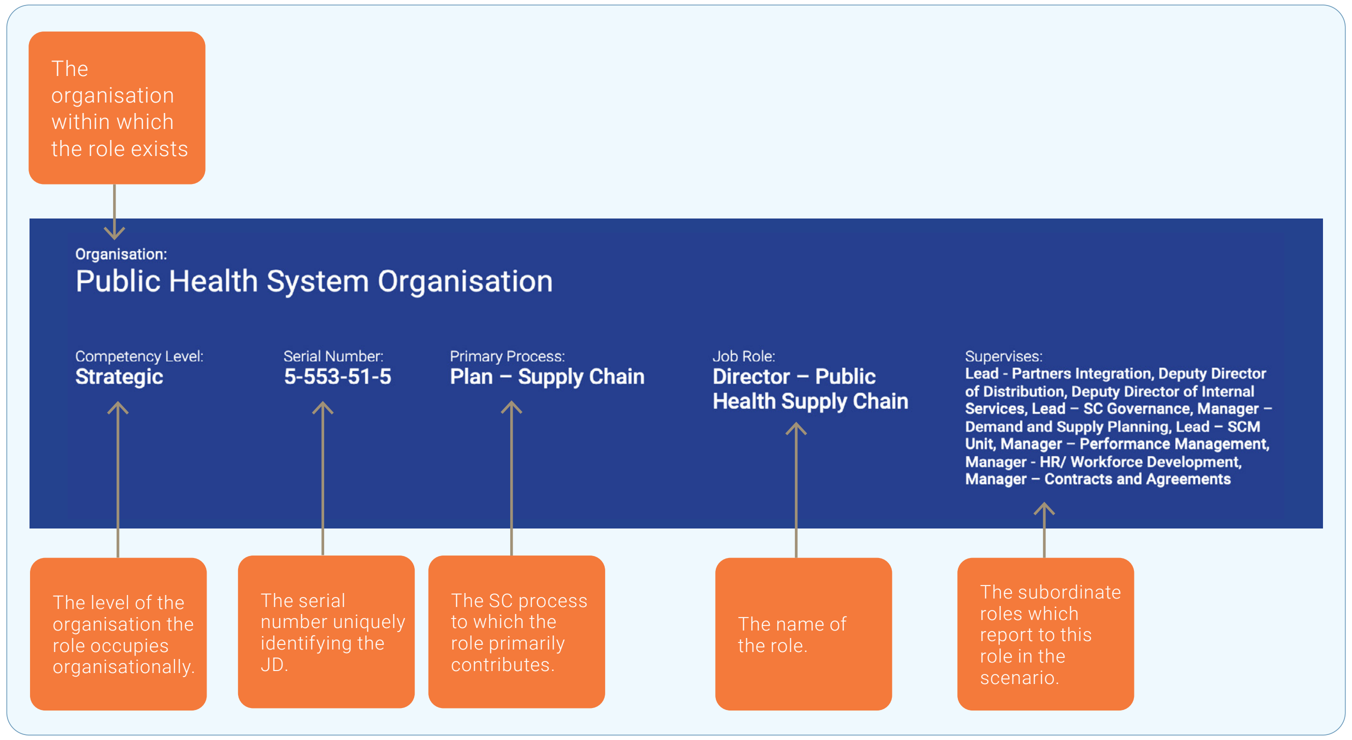

Figure 5:
Description of how the Collection of Roles and Job Descriptions for Health Supply Chains is constructed

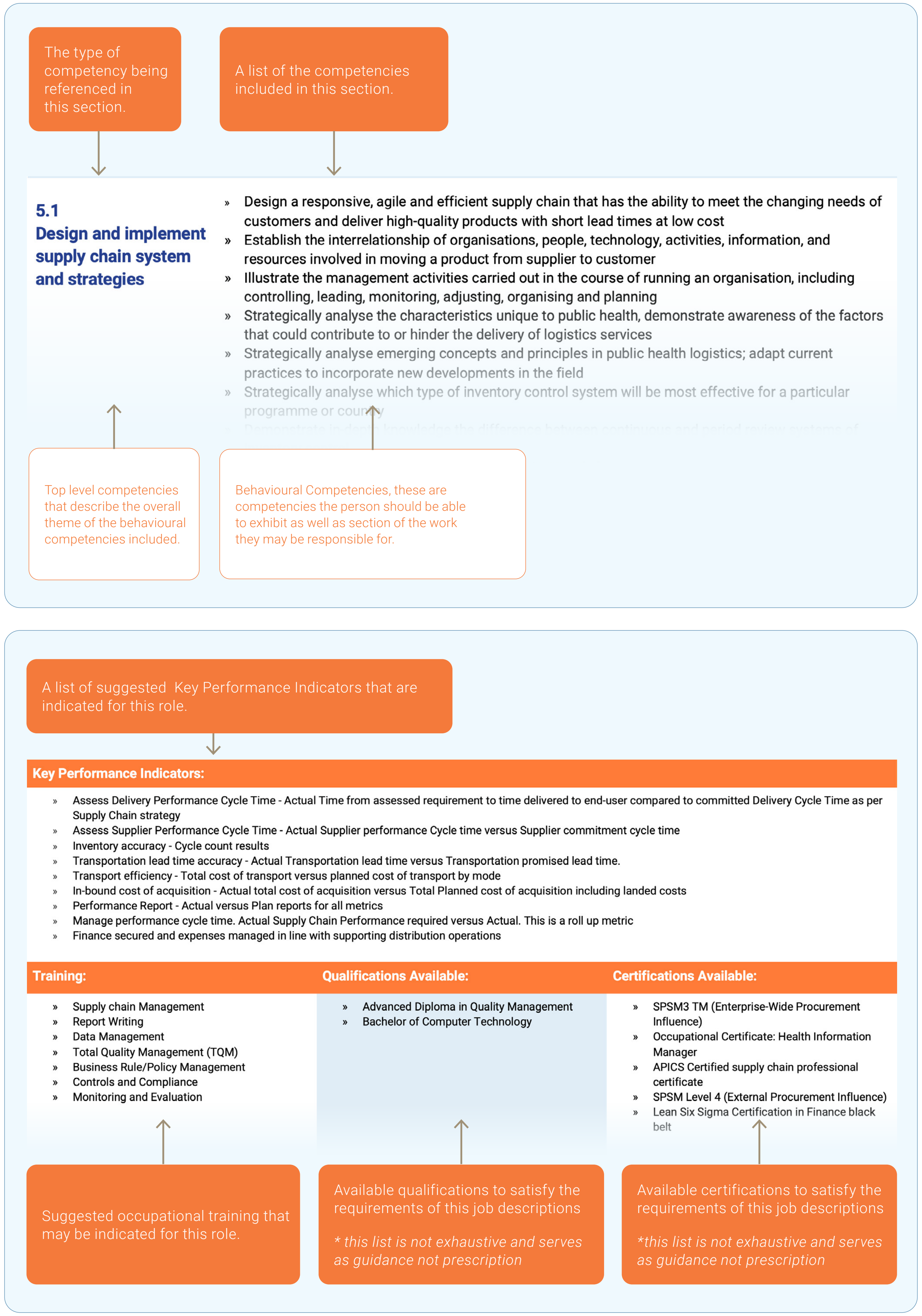

Figure 5 (cont.):
Description of how the Collection of Roles and Job Descriptions for Health Supply Chains is constructed

While these are standardised job roles, it is fully expected that countries will alter and merge the JDs according to their needs.

Level definitions

Organisation levels are defined in the compendium according to the following hierarchy:

Strategic:
Applies strategic, systems thinking; directs and advises, manages change, and influences internal and external stakeholders

Managerial:
Develops, improves and fulfils organisational and functional objectives; manages efficiency, quality and risk

Operational:
Provides guidance on procedures and connected processes

Tactical
Executes the process and assists operational levels to perform their overarching duties.

Organisations

Public health system organisation:
This refers to the organisational structures found in public sector health systems established to deliver health services to country populations. The primary purpose of the organisations that collectively make up the public health system is to ensure access to quality care through programmes targeting men, women, children and populations. As access to quality medicines, supplies and equipment is a core component of care, the public health system also ensures that systems are in place to design, procure, deliver and manage supply chains. This compendium refers to public health system roles that are involved in ensuring functioning supply chain (SC) systems are in place while not necessarily executing the SC functions (these SC functions and roles are captured under the SC organisation type). Meanwhile, this organisation type includes the service delivery levels of a public health system (such as hospital, clinics, and community health posts) that play a role as "customers" of supply chain systems.

The public health system is also typically tiered, where decision making authority for health and SC programming is the purview of a high level structure (a central body or a state or regional structure under devolved health systems), and functions and roles are 'cascaded' to lower-intermediate levels (such as regions in the case of centralised public systems or provinces.

Supply chain (SC) organisation:
This refers to the entities involved in carrying out core SC functions to service the needs of the public health system with quality, timely and adequate medicines, supplies and equipment. The SC organisation may be public sector owned, a parastatal or a privately owned entity. The compendium of roles for this organisation type is intended to reflect the full scope of functions and roles involved to ensure high performing, reliable supply chains. Typically, the SC organisation will include structures in different locations (such as branches or hubs) to more cost-effectively provide SC services closer to populations.

Scenario hierarchy

The Collection of Roles and Job Descriptions for Health Supply Chains provided only displays one possible organisation of job roles in a country and as such some choices have been made to encapsulate one specific scenario and that is a version of a semi-autonomous supply chain organisation. As such it is assumed that the primary seat of supply chain domain knowledge is held by this organisation. Assuming this, the highest level of public health would then perform a regulatory and compliance role keeping the SC organisation in compliance and synchronised with national priorities.

What this means is that any supply chain role within intermediate all the way down to community level will report into the supply chain organisation creating a flow of reporting and information that will align to the public healthcare supply chain operating model. The hierarchy used for this scenario is displayed in Figure 6 below. The organisations in this document are also colour coded according to the below diagram.

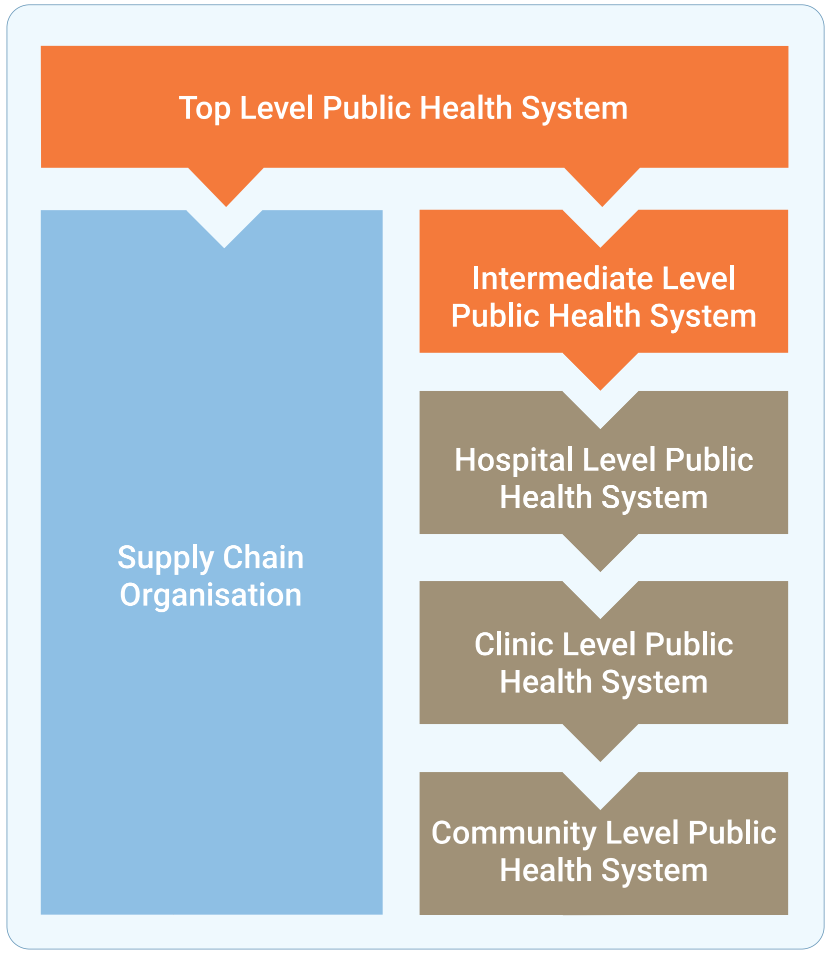

Figure 6:
Depiction of hierarchy used for generation of standardised job descriptions with corresponding colour codes

Mapping of Education for Health Supply Chains

To improve the supply of supply chain professionals there needs to be significant support for it in academia as well as from vocational training providers, certification and professional bodies in-country. To this end PtD has developed a list of available qualifications and certifications that can assist in acquiring the skills needed to be deemed a professional in the healthcare supply chain sector.

This list is by no means exhaustive nor is it prescriptive, it is only a reflection of courses, readily available to the authors at the time, that directly correspond with the skills and competencies specified in the competency framework and job descriptions. It includes more than 250 courses from various providers around the world.

In the Education Framework document, the user will find a similar structure to the competency framework; the difference is that there are no competency descriptors but rather in their place, a list of courses that have exhibited content for those competency descriptors.

The Education Framework is designed to give the user a broad idea of the types of education available to fill the skills gaps found in the country, but it is recognised that each country may have its own regulations and prerequisites.

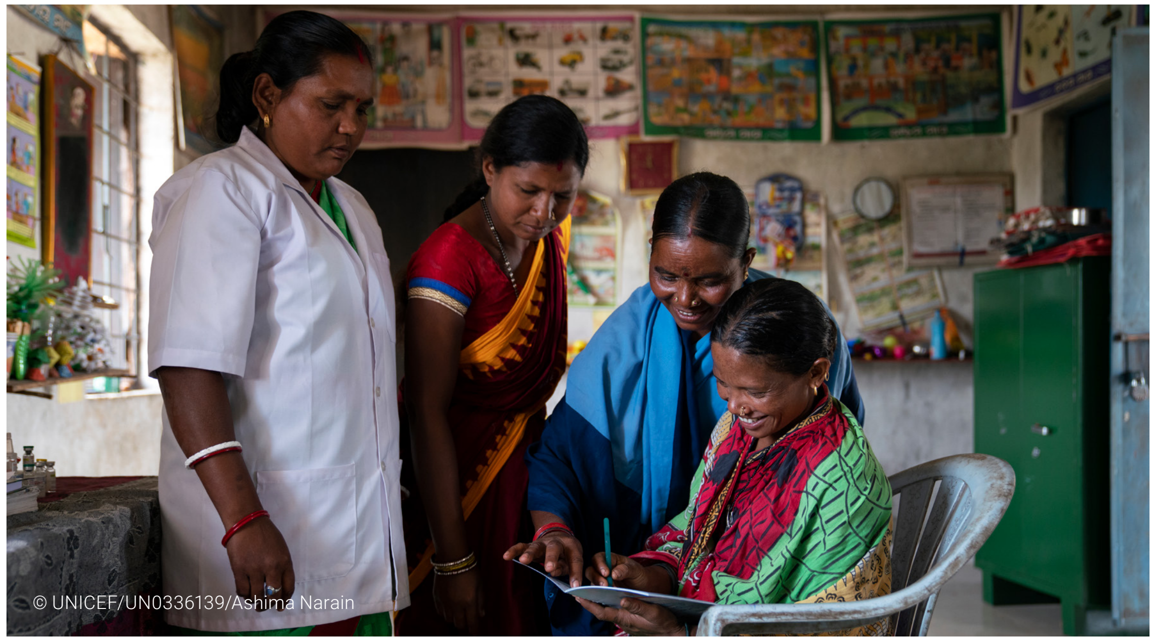
© UNICEF/UN0336139/Ashima Narain

Methodology

In order to develop a Mapping of Education for Health Supply Chains a model needed to be chosen. The author team had a firm grasp and easy access to one of the leading education frameworks in Africa, namely the South African Qualifications Authority (SAQA). SAQA contains more than 14000 qualifications for evaluation spanning various subjects including but not limited to public healthcare. (see Appendix C: SAQA structure)

Thus, because this dataset was unfocussed, the first step was to filter our irrelevant courses. To filter out the irrelevant courses the competency framework was used as a first pass filter: anything that did not directly relate to the competency framework was removed leaving 436 courses that related to the seven domains of the competency framework.

The competency framework comprises more than 3000 competencies across the levels and behavioural competencies, thus in order to speed up the process a set of natural language processing (NLP) algorithms were developed and deployed against the dataset.

The NLP algorithms were used to compare behavioural competencies to the course description, outcomes and overall information contained in the SAQA course records. Where there was significant overlap, the course was said to match the competency; only the top 5% of matches were kept as candidates for the Mapping of Education for Health Supply Chains. Once the first pass with SAQA was completed, certificate courses were added to this list and assigned in a similar method. This had two effects: one was to expand the dataset and the other was to partially validate the model as these certificate courses are more focussed than the degrees from SAQA and thus these were easier to validate. The results of this validation were that less than 2% of allocations made for the certificate programme were judged to be erroneous.

At this point a candidate list was created including SAQA and various certificate courses; the full list of course sources can be found below. Finally, these courses were compared to the competencies that matched them and removed where erroneous matches were observed, which was observed to be less than 5%. Finally validating the model and the final Mapping of Education for Health Supply Chains was finalised.

Courses are listed from:

- Coursera
- MIT
- edukazi.com
- South African Qualifications Authority
- (All registered qualifications in South Africa)
- Empower
- CIPS
- CILT

- I+ Solutions
- ASCM/APICS
- SAPICS
- Next Level Purchasing Association

If you are a training provider, your courses may be included in this list by contacting PtD and supplying the requisite information for merge into this document in later revisions.

An example of how to navigate this framework is displayed below:

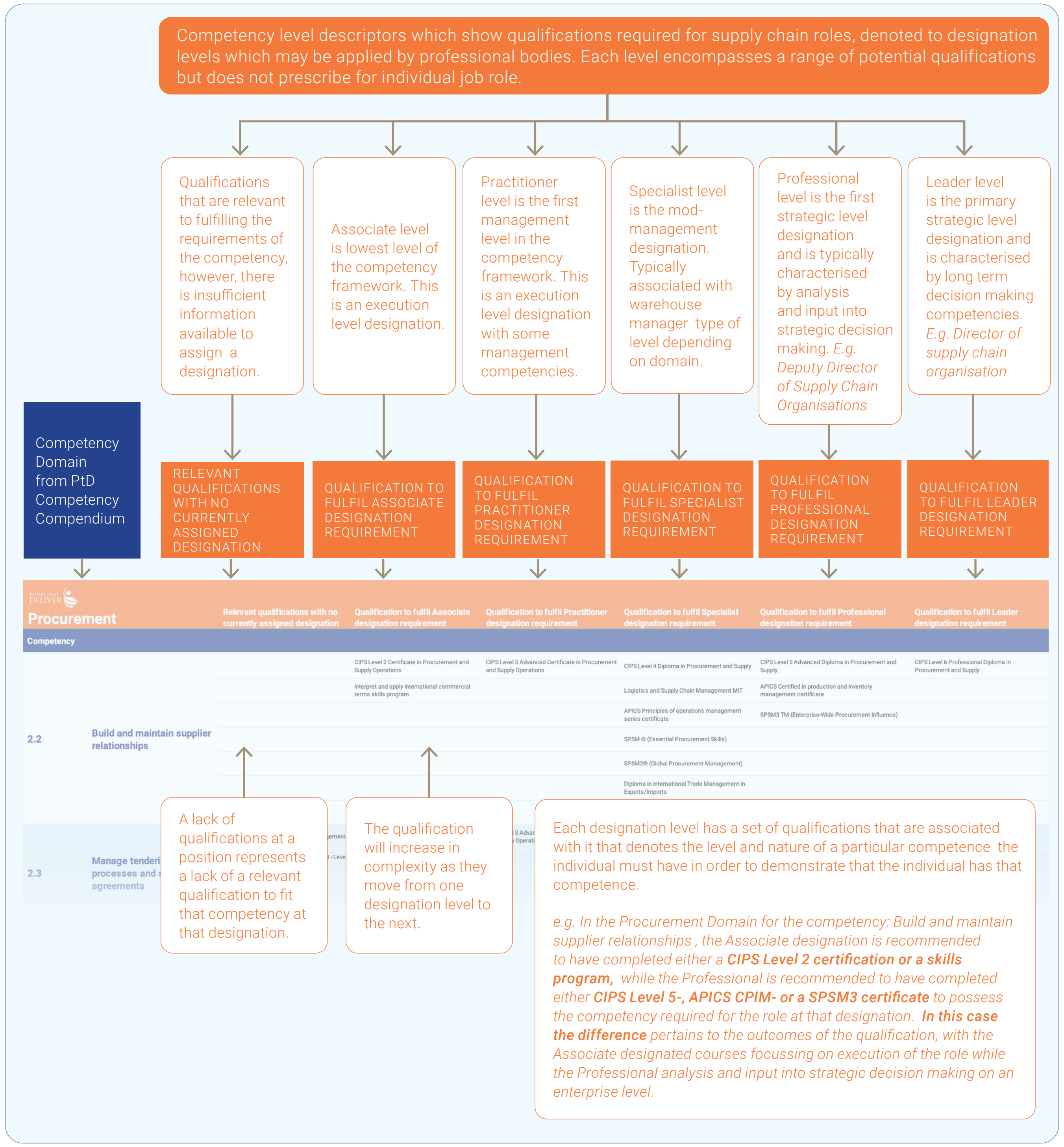

Figure 7:
Description of how the Mapping of Education for Health Supply Chains is constructed

A note on GHSC-PSM

GHSC-PSM recognises that without a strong, skilled workforce at the national and local levels, system-based and technological improvements won't have their intended effect. Achieving a well-performing, motivated workforce requires strengthening the organisational systems, processes, and environment in which supply chain workers perform their duties.

The GHSC-PSM vision is to foster self-sufficient organisations with institutionalised systems. This in turn will help ensure high performance from a professionalised and consumer-centred workforce for effective and efficient delivery of health commodities through to the last mile.

The project goes beyond basic capacity building to look at long-term solutions to organisational and people development, considering the development of human resources systems as an investment. We do this by providing technical support to continuously improve the systems, processes and factors affecting an organisation's ability to plan for, manage and support professionalised national cadres of supply chain professionals.

A note on People that Deliver

With more than 250 organisational members globally, PtD advocates for interventions that improve the demand and supply of a qualified health supply chain professional in organisations, which in turn strengthens the individual practitioners within those organisations. Since 2011, PtD has contributed significantly to the human resources body of knowledge available to health supply chain practitioners. As custodians for the stepped-approach toolkit, which features the Competency Compendium for Health Supply Chain Management, PtD was an obvious partner in considering an SCM professional framework.

A note on SAPICS

SAPICS has taken the step to professionalise SCM in South Africa by assuming the role of the professional body for supply chain management. Having served the profession for 50 years, it is well positioned to provide the services of the professional body that will see it uplift supply chain management as a profession as well as the practices and people within it. It also assists in fostering relationships with government to assist in addressing strategic imperatives for economic transformation in South Africa and the continent of Africa more broadly. SAPICS awards professional designations based on technical supply chain competencies.

Appendices

A	Organisation participation or past study organisations	27
B	Library of Competencies & Designations for Health Supply Chains	28
C	SAQA structure	95

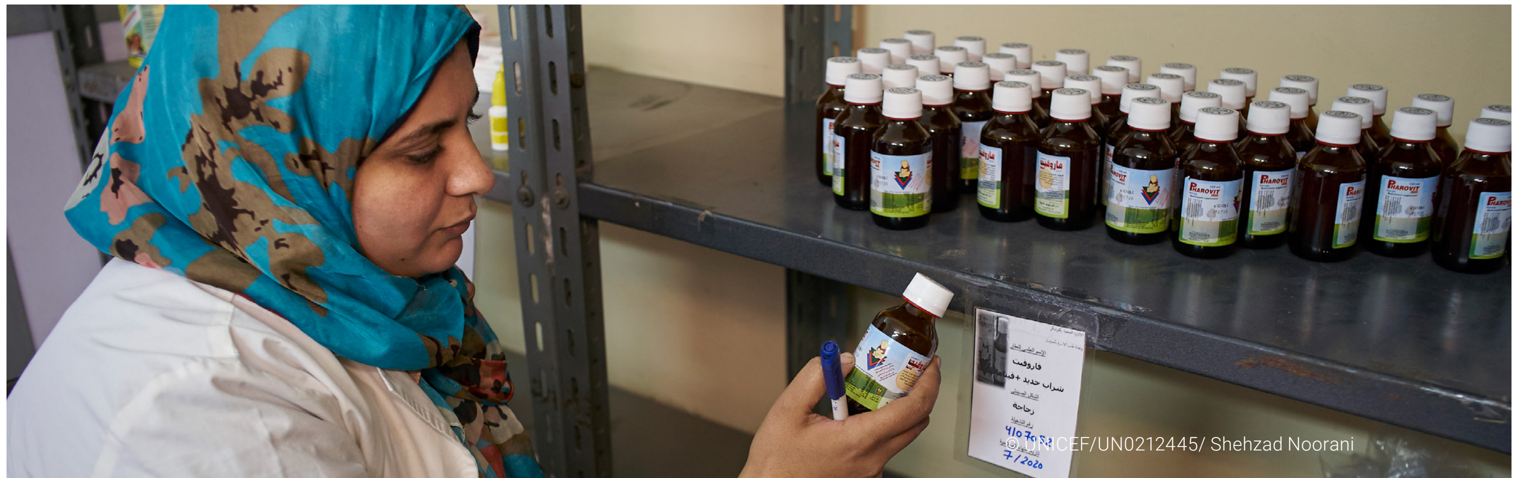

UNICEF/UN0212445/ Shehzad Noorani

Appendix A

Organisation participation or past study organisations

SAPICS:
The professional body for supply chain management

UNICEF

DSV Healthcare

GHSC-PSM

Mozambique, Haiti, Rwanda, Myanmar, Cameroon

Appendix B

Library of Competencies & Designations for Health Supply Chains

1. Selection and quantification

COMPETENCY	BEHAVIOURAL COMPETENCIES	Associate	Practitioner	Specialist	Professional	Leader
1.1 Select the appropriate product	Describe the broad concepts of the national medicines policy, essential medicines lists, essential equipment lists, standard treatment guidelines and "dangerous drug" (DDA) policy	Awareness of the broad concepts of the national medicines policy, essential medicines lists, essential equipment lists, standard treatment guidelines and "dangerous drug" (DDA) policy	Compare the broad concepts of the national medicines policy, essential medicines lists, essential equipment lists, standard treatment guidelines and "dangerous drug" (DDA) policy	Describe the broad concepts of the national medicines policy, essential medicines lists, essential equipment lists, standard treatment guidelines and "dangerous drug" (DDA) policy	Analyse the broad concepts of the national medicines policy, essential medicines lists, essential equipment lists, standard treatment guidelines and "dangerous drug" (DDA) policy	Review the broad concepts of the national medicines policy, essential medicines lists, essential equipment lists, standard treatment guidelines and "dangerous drug" (DDA) policy
	Use the government system required to add and subtract items from the essential medicines list and the essential equipment list	Basic operational knowledge of the government system required to add and subtract items from the essential medicines list and the essential equipment list	Understand the government system required to add and subtract items from the essential medicines list and the essential equipment list	Use the government system required to add and subtract items from the essential medicines list and the essential equipment list	Analyse the government system required to add and subtract items from the essential medicines list and the essential equipment list	Direct and review the government system required to add and subtract items from the essential medicines list and the essential equipment list
	Follow the government system required to alter standard treatment guidelines, dangerous drug policy and national medicines policy	Awareness that the government system is required to alter standard treatment guidelines, dangerous drug policy and national medicines policy	Understand that the government system required to alter standard treatment guidelines, dangerous drug policy and national medicines policy	Follow the government system required to alter standard treatment guidelines, dangerous drug policy and national medicines policy	Ensure adherence the government system required to alter standard treatment guidelines, dangerous drug policy and national medicines policy	Implement the government system required to alter standard treatment guidelines, dangerous drug policy and national medicines policy
	Confirm the type of supplies and services that are required	Awareness of the type of supplies and services that are required	Compare the type of supplies and services that are required	Confirm the type of supplies and services that are required	Determine the type of supplies and services that are required	Strategically analyse the type of supplies and services that are required

COMPETENCY	BEHAVIOURAL COMPETENCIES	Associate	Practitioner	Specialist	Professional	Leader
1.1 Select the appropriate product	Convene regularly scheduled coordination meetings with stakeholders involved in financing, procuring or distributing commodities	Awareness of regularly scheduled coordination meetings with stakeholders involved in financing, procuring or distributing commodities	Provide input for regularly scheduled coordination meetings with stakeholders involved in financing, procuring or distributing commodities	Participate in regularly scheduled coordination meetings with stakeholders involved in financing, procuring or distributing commodities	Convene regularly scheduled coordination meetings with stakeholders involved in financing, procuring or distributing commodities	Direct regularly scheduled coordination meetings with stakeholders involved in financing, procuring or distributing commodities
	Understand the steps needed to bring a medicinal product to the market, including the safety, quality, efficacy and pharmacoeconomic assessments of the product	Basic operational knowledge of the steps needed to bring a medicinal product to the market, including the safety, quality, efficacy and pharmacoeconomic assessments of the product	Understand the steps needed to bring a medicinal product to the market, including the safety, quality, efficacy and pharmacoeconomic assessments of the product	Describe the steps needed to bring a medicinal product to the market, including the safety, quality, efficacy and pharmacoeconomic assessments of the product	Evaluate the steps needed to bring a medicinal product to the market, including the safety, quality, efficacy and pharmacoeconomic assessments of the product	Strategically analyse the steps needed to bring a medicinal product to the market, including the safety, quality, efficacy and pharmacoeconomic assessments of the product
1.2 Define the specifications of the product, including product quality	Advise on specifications for procurement	Awareness of specifications for procurement	Understand specifications for procurement	Define specifications for procurement	Advise on specifications for procurement	Develop specifications for procurement
	Describe the characteristics of a good specification	Awareness of the characteristics of a good specification	Compare the characteristics of a good specification	Describe the characteristics of a good specification	Analyse the characteristics of a good specification	Demonstrate the characteristics of a good specification
	Discuss the types of specifications	Awareness of the types of specifications	Compare the types of specifications	Discuss the types of specifications	Determine the types of specifications	Strategically analyse the types of specifications
	Name the advantages of functional and performance specifications	Awareness of the advantages of functional and performance specifications	Name the advantages of functional and performance specifications	Identify the advantages of functional and performance specifications	Determine the advantages of functional and performance specifications	Demonstrate the advantages of functional and performance specifications
	List the contents of a specification	Basic operational knowledge of the contents of a specification	Examine the contents of a specification	List the contents of a specification	Evaluate the contents of a specification	Strategically analyse the contents of a specification
	Name the procurement staff responsibilities in the specification process	Awareness of the procurement staff responsibilities in the specification process	Name the procurement staff responsibilities in the specification process	Identify the procurement staff responsibilities in the specification process	Analyse the procurement staff responsibilities in the specification process	Define the procurement staff responsibilities in the specification process
1.3 List any special considerations for the product (e.g. temperature requirements, size, implications for infrastructure)	Describe the principles and processes of category management, including market segmentation principles	Basic operational knowledge the principles and processes of category management, including market segmentation principles	Compare the principles and processes of category management, including market segmentation principles	Describe the principles and processes of category management, including market segmentation principles	Evaluate the principles and processes of category management, including market segmentation principles	Strategically analyse the principles and processes of category management, including market segmentation principles
	List any specific considerations in the quantification of programme-specific products (e.g. ARVs, family planning commodities, vaccines)	Awareness of any specific considerations in the quantification of programme-specific products (e.g. ARVs, family planning commodities, vaccines)	Examine any specific considerations in the quantification of programme-specific products (e.g. ARVs, family planning commodities, vaccines)	List any specific considerations in the quantification of programme-specific products (e.g. ARVs, family planning commodities, vaccines)	Determine any specific considerations in the quantification of programme-specific products (e.g. ARVs, family planning commodities, vaccines)	Review specific considerations in the quantification of programme-specific products (e.g. ARVs, family planning commodities, vaccines)
	Describe current international trends in commodity availability	Awareness of current international trends in commodity availability	Examine current international trends in commodity availability	Describe current international trends in commodity availability	Evaluate current international trends in commodity availability	Strategically analyse current international trends in commodity availability

COMPETENCY	BEHAVIOURAL COMPETENCIES	Associate	Practitioner	Specialist	Professional	Leader
1.4 Forecast and quantify product needs	Identify the factors that affect usage patterns of medications and equipment and how this affects ordering (e.g. disease outbreaks), using national policies as a guide and to ensure consistent application	Awareness of the factors that affect usage patterns of medications and equipment and how this affects ordering (e.g., disease outbreaks), using national policies as a guide and to ensure consistent application	Examine the factors that affect usage patterns of medications and equipment and how this affects ordering (e.g., disease outbreaks), using national policies as a guide and to ensure consistent application	Identify the factors that affect usage patterns of medications and equipment and how this affects ordering (e.g., disease outbreaks), using national policies as a guide and to ensure consistent application	Determine the factors that affect usage patterns of medications and equipment and how this affects ordering (e.g., disease outbreaks), using national policies as a guide and to ensure consistent application	Strategically analyse the factors that affect usage patterns of medications and equipment and how this affects ordering (e.g., disease outbreaks), using national policies as a guide and to ensure consistent application
	Understand principles and applications of demand forecasting	Basic operational knowledge of the principles and applications of demand forecasting	Understand principles and applications of demand forecasting	Apply principles and applications of demand forecasting	Analyse principles and applications of demand forecasting	Strategically analyse and review principles and applications of demand forecasting for application
	Demonstrate the critical requirements for effective forecasting: establishing time horizons, level of detail and use of data	Basic operational knowledge of the critical requirements for effective forecasting: establishing time horizons, level of detail and use of data	Compare the critical requirements for effective forecasting: establishing time horizons, level of detail and use of data	Apply the critical requirements for effective forecasting: establishing time horizons, level of detail and use of data	Demonstrate the critical requirements for effective forecasting: establishing time horizons, level of detail and use of data	Implement the critical requirements for effective forecasting: establishing time horizons, level of detail and use of data
	Establish policies and procedures for forecasting	Awareness of the policies and procedures for forecasting	Understand policies and procedures for forecasting	Describe policies and procedures for forecasting	Analyse policies and procedures for forecasting	Establish policies and procedures for forecasting
	Quantify product requirements using a variety of methods	Awareness of the process of quantifying product requirements using a variety of methods	Understand the process of quantifying product requirements using a variety of methods	Quantify product requirements using a variety of methods	Analyse the process of quantifying product requirements using a variety of methods	Implement the process of quantifying product requirements using a variety of methods
	Organize and summarize consumption data at the national level for various country programmes	Awareness of consumption data at the national level for various country programmes	Examine consumption data at the national level for various country programmes	Organize and summarize consumption data at the national level for various country programmes	Evaluate consumption data at the national level for various country programmes	Strategically analyse consumption data at the national level for various country programmes
	Compare and reconcile different types of forecasts	Awareness of different types of forecasts	Compare and reconcile different types of forecasts	Describe the use different types of forecasts	Evaluate different types of forecasts	Strategically analyse different types of forecasts
	Apply VEN or ABC analysis to programme requirements for national level procurement	Awareness of the process of applying VEN or ABC analysis to programme requirements for national level procurement	Understand the process of applying VEN or ABC analysis to programme requirements for national level procurement	Apply VEN or ABC analysis to programme requirements for national level procurement	Analyse the VEN or ABC analysis to programme requirements for national level procurement	Implement the VEN or ABC analysis to programme requirements for national level procurement
	Calculate average monthly dispensed-to-user quantities for all service delivery points nationally	Awareness of the importance of calculating average monthly dispensed-to-user quantities for all service delivery points nationally	Understand the importance of calculating average monthly dispensed-to-user quantities for all service delivery points nationally	Calculate average monthly dispensed-to-user quantities for all service delivery points nationally	Evaluate methods of calculating average monthly dispensed-to-user quantities for all service delivery points nationally	Implement the process of calculating average monthly dispensed-to-user quantities for all service delivery points nationally
	Calculate storage space requirements for all levels in the supply chain	Awareness of the importance of calculating storage space requirements for all levels in the supply chain	Understand the importance of calculating storage space requirements for all levels in the supply chain	Calculate storage space requirements for all levels in the supply chain	Evaluate methods of calculating storage space requirements for all levels in the supply chain	Implement the process of calculating storage space requirements for all levels in the supply chain
	Calculate the months of stock, average monthly consumption and stock on hand for each commodity at the national level	Awareness of the importance of calculating the months of supply on hand for each commodity at the national level	Understand the importance of calculating the months of supply on hand for each commodity at the national level	Calculate the months of supply on hand for each commodity at the national level	Evaluate methods of calculating the months of supply on hand for each commodity at the national level	Implement the process of calculating the months of supply on hand for each commodity at the national level

COMPETENCY	BEHAVIOURAL COMPETENCIES	Associate	Practitioner	Specialist	Professional	Leader
1.4 Forecast and quantify product needs (cont.)	Determine the maximum and minimum stock levels for each level in a programme	Awareness of the maximum and minimum stock levels for each level in a programme	Examine the maximum and minimum stock levels for each level in a programme	Describe the maximum and minimum stock levels for each level in a programme	Determine the maximum and minimum stock levels for each level in a programme	Strategically analyse the maximum and minimum stock levels for each level in a programme
	Demonstrate knowledge about various tools used in quantification (e.g. quantification software)	Awareness of various tools used in quantification (e.g. quantification software)	Understand various tools used in quantification (e.g. quantification software)	Use various tools used in quantification (e.g. quantification software)	Demonstrate knowledge about various tools used in quantification (e.g. quantification software)	Demonstrate in-depth knowledge about various tools used in quantification (e.g. quantification software)
	Demonstrate the use of various data sources for commodity forecasting (e.g. consumption data, services data, demographic data)	Basic operational knowledge on the use of various data sources for commodity forecasting (e.g., consumption data, services data, demographic data)	Understand the use of various data sources for commodity forecasting (e.g., consumption data, services data, demographic data)	Describe the use of various data sources for commodity forecasting (e.g., consumption data, services data, demographic data)	Demonstrate the use of various data sources for commodity forecasting (e.g., consumption data, services data, demographic data)	Implement the use of various data sources for commodity forecasting (e.g., consumption data, services data, demographic data)
	Establish key performance indicators of forecast accuracy	Awareness of key performance indicators of forecast accuracy	Compare key performance indicators of forecast accuracy	Establish key performance indicators of forecast accuracy	Determine key performance indicators of forecast accuracy	Develop key performance indicators of forecast accuracy
	Establish the degree of error when using forecasting methods	Awareness of the degree of error when using forecasting methods	Examine the degree of error when using forecasting methods	Establish the degree of error when using forecasting methods	Determine the degree of error when using forecasting methods	Strategically analyse the degree of error when using forecasting methods

COMPETENCY	BEHAVIOURAL COMPETENCIES	Associate	Practitioner	Specialist	Professional	Leader
1.5. Supply planning	Design, implement and monitor a purchasing and supply plan in line with national priorities	Basic operational knowledge of a purchasing and supply plan in line with national priorities	Examine a purchasing and supply plan in line with national priorities	Describe a purchasing and supply plan in line with national priorities	Demonstrate a purchasing and supply plan in line with national priorities	Design, implement and monitor a purchasing and supply plan in line with national priorities
	Balance supply chain resources and trigger activities to correct any imbalances	Awareness of the importance of balancing of supply chain resources and trigger activities to correct any imbalances	Understand the importance of balancing of supply chain resources and trigger activities to correct any imbalances	Balance supply chain resources and trigger activities to correct any imbalances	Evaluate the balance supply chain resources and trigger activities to correct any imbalances	Design the process of balancing the supply chain resources and trigger activities to correct any imbalances
	Formalise the sales and operations plans through sales and operations meeting.	Awareness of the formalisation of sales and operations plans through sales and operations meeting.	Understand the formalisation of sales and operations plans through sales and operations meeting.	Explain the formalisation of sales and operations plans through sales and operations meeting.	Analyse the formalisation of sales and operations plans through sales and operations meeting.	Design the formalisation of sales and operations plans through sales and operations meeting.
	Translate sales and operations plan into executable plans to ensure dispatch of goods in line with overall priorities.	Awareness that the sales and operations plan must be translated into executable plans to ensure dispatch of goods in line with overall priorities.	Understand that the sales and operations plan must be translated into executable plans to ensure dispatch of goods in line with overall priorities.	Translate sales and operations plan into executable plans to ensure dispatch of goods in line with overall priorities.	Evaluate the translation of sales and operations plan into executable plans to ensure dispatch of goods in line with overall priorities.	Design processes for the translation of sales and operations plan into executable plans to ensure dispatch of goods in line with overall priorities.
	Monitor and manage the national commodity pipeline	Awareness of the national commodity pipeline	Understand the national commodity pipeline	Describe the national commodity pipeline	Monitor and manage the national commodity pipeline	Design and optimise the national commodity pipeline

2. Procurement

COMPETENCY	BEHAVIOURAL COMPETENCIES	Associate	Practitioner	Specialist	Professional	Leader
**2.1 Manage procurement costs and budget**	Follow public procurement regulations	Awareness of public procurement regulations	Understand public procurement regulations	Incorporate and follow public procurement regulations	Ensure the organisation follows public procurement regulations	Define processes to ensure the organisation follows public procurement regulations
	Ensure budget approval is in place before initiating procurement	Awareness of the fact that there is an approved procurement budget	Understand the importance of budget approval before initiating procurement	Ensure budget approval is in place before initiating procurement	Ensure adherence to the approved procurement budget	Define and approve the procurement budget
	Demonstrate sound understanding and application of financial and management accounting practices within procurement	Awareness of the importance of financial and management accounting practices within procurement	Understanding the importance of financial and management accounting practices within procurement	Demonstrate sound understanding and application of financial and management accounting practices within procurement	Evaluate financial and management accounting practices within procurement	Define the financial and management accounting practices within procurement
	Apply commercial factors that contribute toward cost reduction, price savings and value improvement opportunities	Awareness of commercial factors that contribute toward cost reduction, price savings and value improvement opportunities	Understand the commercial factors that contribute toward cost reduction, price savings and value improvement opportunities	Apply commercial factors that contribute toward cost reduction, price savings and value improvement opportunities	Analyse commercial factors that contribute toward cost reduction, price savings and value improvement opportunities	Strategically evaluate commercial factors that contribute toward cost reduction, price savings and value improvement opportunities
	Factor in principles of foreign exchange rates and how they impact prices	Awareness of foreign exchange rates and how they impact prices	Understand the principles of foreign exchange rates and how they impact prices	Describe the principles of foreign exchange rates and how they impact prices	Analyse the principles of foreign exchange rates and how they impact prices	In-depth knowledge of foreign exchange rate principles and how they impact prices
**2.2 Build and maintain supplier relationships**	Analyse the market	Awareness of the factors in the analysis of the market	Understand the factors in the analysis of the market	Describe factors in the analysis of the market	Analyse the market	Develop processes for the analysis of the market
	Use and monitor the processes for prequalification and tender contracting	Execute the processes for prequalification and tender contracting	Understand the processes for prequalification and tender contracting	Use and monitor the processes for prequalification and tender contracting	Analyse adherence to the processes for prequalification and tender contracting	Define the processes for prequalification and tender contracting, as well as the monitoring of them
	Define a good supplier	Awareness of factors that define a good supplier	Understand the factors that define a good supplier	Define of a good supplier	Determine the factors that define a good supplier	Strategically analyse the factors that define a good supplier
	Effectively locate and source key suppliers, while analysing the total cost associated with procuring an item or service	Awareness that key suppliers must be located and that there is a total cost associated with procuring an item or service	Understand that key suppliers must be located and that there is a total cost associated with procuring an item or service	Describe the process of locating and sourcing key suppliers, keeping in mind the total cost associated with procuring an item or service	Effectively locate and source key suppliers, while analysing the total cost associated with procuring an item or service	Define the methodology to locate and source key suppliers, while analysing the total cost associated with procuring an item or service
	Develop and implement supplier relationship management plans (e.g. sourcing, frequency of meetings, negotiate and monitor benchmarks for performance)	Awareness of supplier relationship management plans (e.g. sourcing, frequency of meetings, negotiate and monitor benchmarks for performance)	Understand supplier relationship management plans (e.g. sourcing, frequency of meetings, negotiate and monitor benchmarks for performance)	Describe supplier relationship management plans (e.g. sourcing, frequency of meetings, negotiate and monitor benchmarks for performance)	Analyse supplier relationship management plans (e.g. sourcing, frequency of meetings, negotiate and monitor benchmarks for performance)	Develop and implement supplier relationship management plans (e.g. sourcing, frequency of meetings, negotiate and monitor benchmarks for performance)

COMPETENCY	BEHAVIOURAL COMPETENCIES	Associate	Practitioner	Specialist	Professional	Leader
**2.2 Build and maintain supplier relationships (cont.)**	Educate suppliers to create value for customers by streamlining processes in the value chain	Awareness that suppliers need to be educated in order to create value for customers by streamlining processes in the value chain	Understand the process of educating suppliers to create value for customers by streamlining processes in the value chain	Educate suppliers to create value for customers by streamlining processes in the value chain	Analyse of the process of educating suppliers to create value for customers by streamlining processes in the value chain	Develop of the process to educate suppliers to create value for customers by streamlining processes in the value chain
	Explain the difference between strategic and non-strategic suppliers and the associated supplier management principles	Awareness of the difference between strategic and non-strategic suppliers and the associated supplier management principles	Understand the difference between strategic and non-strategic suppliers and the associated supplier management principles	Explain the difference between strategic and non-strategic suppliers and the associated supplier management principles	Analyse the difference between strategic and non-strategic suppliers and the associated supplier management principles	Define the process of differentiating the strategic and non-strategic suppliers and the associated supplier management principles
**2.3 Manage tendering processes and supplier agreements**	Demonstrate the use of request for qualifications (RFQ), invitations to bid (ITB) and request for proposals (RFP) methods and when to choose which method	Awareness of the use of request for qualifications (RFQ), invitations to bid (ITB) and request for proposals (RFP) methods and the fact that they are chosen	Understand the use of request for qualifications (RFQ), invitations to bid (ITB) and request for proposals (RFP) methods and when to choose which method	Demonstrate the correct use of request for qualifications (RFQ), invitations to bid (ITB) and request for proposals (RFP) methods	Evaluate when to the use of request for qualifications (RFQ), invitations to bid (ITB) and request for proposals (RFP) methods	Define the rules around when to use of request for qualifications (RFQ), invitations to bid (ITB) and request for proposals (RFP)
	Demonstrate knowledge of local and international tendering procedures and select procurement strategy and methods appropriate to special commodities and contextual situations	Awareness of local and international tendering procedures (e.g., open competitive bidding, restricted tender, competitive negotiation, direct procurement)	Understand the local and international tendering procedures (e.g., open competitive bidding, restricted tender, competitive negotiation, direct procurement)	Demonstrate knowledge of local and international tendering procedures (e.g., open competitive bidding, restricted tender, competitive negotiation, direct procurement)	Demonstrate in-depth knowledge of local and international tendering procedures and analyse the procurement strategy and methods appropriate to special commodities and contextual situations	Demonstrate in-depth knowledge of local and international tendering procedures and select procurement strategy and methods appropriate to special commodities and contextual situations
	Write detailed specifications for tenders	Awareness of the detailed specifications for tenders	Understand the detailed specifications for tenders	Write detailed specifications for tenders	Determine the detailed specifications for tenders	Review and implement the process of writing detailed specifications for tenders
	Provide high-level guidance for high-value and politically sensitive procurements	Awareness of the reason for high-level guidance when purchasing high-value and politically sensitive procurements	Understand the need for high-level guidance for high-value and politically sensitive procurements	Describe the high-level guidance required for high-value and politically sensitive procurements	Provide operational guidance for high-value and politically-sensitive procurements	Provide high level guidance for high-value and politically-sensitive procurements
	Facilitate the process of developing and managing contracts	Awareness that there is a process of developing and managing contracts	Understand the process of developing and managing contracts	Describe the process of developing and managing contracts	Facilitate the process of developing and managing contracts	Define the process of developing and managing contracts
	Formulate procurement, return and exchange policies consistent with approved financial delegation and sound business practice	Awareness of procurement, return and exchange policies	Understand the procurement, return and exchange policies	Describe the procurement, return and exchange policies	Ensure adherence to the procurement, return and exchange policies	Formulate procurement, return and exchange policies consistent with approved financial delegation and sound business practice

COMPETENCY	BEHAVIOURAL COMPETENCIES	Associate	Practitioner	Specialist	Professional	Leader
2.3 Manage tendering processes and supplier agreements (cont.)	List appropriate legislation and policy in regard to procurement processes, and what is required to comply	Basic operational knowledge of appropriate legislation and policy in regard to procurement processes, and what is required to comply	Understand appropriate legislation and policy in regard to procurement processes, and what is required to comply	List appropriate legislation and policy in regard to procurement processes, and what is required to comply	Ensure adherence to the appropriate legislation and policy in regard to procurement processes, and what is required to comply	Understand the strategic impact of compliance regarding appropriate legislation and policy in regard to procurement processes
	Ensure ethical behaviour, abiding by conflict of interest policies	Awareness of ethical behaviour and conflict of interest policies	Understand what constitutes ethical behaviour, abiding by conflict of interest policies	Ensure ethical behaviour, abiding by conflict of interest policies	Demonstrate ethical behaviour, abiding by conflict of interest policies	Determine what constitutes ethical behaviour, abiding by conflict of interest policies
	Evaluate tender bids based on the specification and evaluation criteria including capability of the supplier, problems relating to outcomes of the contract.	Awareness of the process of evaluating tender bids based on the specification and evaluation criteria including capability of the supplier, problems relating to outcomes of the contract.	Understand the process of evaluating tender bids based on the specification and evaluation criteria including capability of the supplier, problems relating to outcomes of the contract.	Describe the process of evaluating tender bids based on the specification and evaluation criteria including capability of the supplier, problems relating to outcomes of the contract.	Evaluate tender bids based on the specification and evaluation criteria including capability of the supplier, problems relating to outcomes of the contract.	Direct the process of evaluating tender bids based on the specification and evaluation criteria including capability of the supplier, problems relating to outcomes of the contract.
	Follow contract approval process, including contract negotiations	Awareness that there is a contract approval process, including contract negotiations	Understand that there is a contract approval process, including contract negotiations	Follow contract approval process, including contract negotiations	Evaluate the contract approval process, including contract negotiations	Develop the contract approval process, including contract negotiations
	Discuss some of the key issues relevant to negotiating supply contracts (e.g. volume usage over time, price volume agreements, alternate supplier clauses, duration of contract, period of review, terms for contract termination and renewal)	Awareness of some of the key issues relevant to negotiating supply contracts (e.g. volume usage over time, price volume agreements, alternate supplier clauses, duration of contract, period of review, terms for contract termination and renewal)	Understand some of the key issues relevant to negotiating supply contracts (e.g. volume usage over time, price volume agreements, alternate supplier clauses, duration of contract, period of review, terms for contract termination and renewal)	Discuss some of the key issues relevant to negotiating supply contracts (e.g. volume usage over time, price volume agreements, alternate supplier clauses, duration of contract, period of review, terms for contract termination and renewal)	Determine some of the key issues relevant to negotiating supply contracts (e.g. volume usage over time, price volume agreements, alternate supplier clauses, duration of contract, period of review, terms for contract termination and renewal)	Strategically analyse some of the key issues relevant to negotiating supply contracts (e.g. volume usage over time, price volume agreements, alternate supplier clauses, duration of contract, period of review, terms for contract termination and renewal)
	Award and manage contracts, resolving any issues as they arise	Awareness that contracts are awarded and managed, and that any issues that arise need to be resolved	Understand the process of awarding and managing contracts, resolving any issues as they arise	Describe the process of awarding and managing contracts, resolving any issues as they arise	Award and manage contracts, resolving any issues as they arise	Direct the process of awarding and managing contracts, resolving any issues as they arise
	Describe the process of debriefing unsuccessful suppliers	Awareness of the process of debriefing unsuccessful suppliers	Understand the process of debriefing unsuccessful suppliers	Describe the process of debriefing unsuccessful suppliers	Analyse the process of debriefing unsuccessful suppliers	Develop the process of debriefing unsuccessful suppliers
	Describe the ways in which compliance with purchasing policies and procedures is monitored	Awareness of the ways in which compliance with purchasing policies and procedures is monitored	Understand the ways in which compliance with purchasing policies and procedures is monitored	Describe the ways in which compliance with purchasing policies and procedures is monitored	Analyse the ways in which compliance with purchasing policies and procedures is monitored	Implement the ways in which compliance with purchasing policies and procedures is monitored

COMPETENCY	BEHAVIOURAL COMPETENCIES	Associate	Practitioner	Specialist	Professional	Leader
2.3 Manage tendering processes and supplier agreements (cont.)	Participate in procurement planning including a yearly activities schedule and developing a wide range of briefs, specifications and commercial documentation	Awareness of the process of procurement planning including a yearly activities schedule and developing a wide range of briefs, specifications and commercial documentation	Understand the process of procurement planning including a yearly activities schedule and developing a wide range of briefs, specifications and commercial documentation	Participate in procurement planning including a yearly activities schedule and developing a wide range of briefs, specifications and commercial documentation	Analyse procurement planning including a yearly activities schedule and developing a wide range of briefs, specifications and commercial documentation	Lead procurement planning including a yearly activities schedule and developing a wide range of briefs, specifications and commercial documentation
	Lead strategic sourcing projects, capturing benefits, work requirements, impacts and cost savings	Basic operational knowledge strategic sourcing projects, capturing benefits, work requirements, impacts and cost savings	Examine strategic sourcing projects, capturing benefits, work requirements, impacts and cost savings	Identify strategic sourcing projects, capturing benefits, work requirements, impacts and cost savings	Analyse strategic sourcing projects, capturing benefits, work requirements, impacts and cost savings	Lead strategic sourcing projects, capturing benefits, work requirements, impacts and cost savings
	Play a leading role in cross-functional teams handling strategic procurements	Awareness of cross-functional teams handling strategic procurements	Provide input to cross-functional teams handling strategic procurements	Participate in cross-functional teams handling strategic procurements	Play a leading role in cross-functional teams handling strategic procurements	Enable the creation of cross-functional teams handling strategic procurements
	Participate in effective internal and external procurement networks	Awareness of internal and external procurement networks	Compare internal and external procurement networks	Describe effective internal and external procurement networks	Participate in effective internal and external procurement networks	Direct participation in effective internal and external procurement networks
	List local and international health commodity prices and understand the factors affecting them	Awareness of local and international health commodity prices and the factors affecting them	Compare local and international health commodity prices and understand the factors affecting them	List local and international health commodity prices and understand the factors affecting them	Analyse local and international health commodity prices and understand the factors affecting them	Demonstrate in-depth knowledge of local and international health commodity prices and understand the factors affecting them
	Engage clients across the public sector in presentations demonstrating the benefits of a range of strategic projects designed to ensure cost savings and value for money for government procurement	Awareness of the process of engaging clients across the public sector in presentations demonstrating the benefits of a range of strategic projects designed to ensure cost savings and value for money for government procurement	Understand the process of engaging clients across the public sector in presentations demonstrating the benefits of a range of strategic projects designed to ensure cost savings and value for money for government procurement	Describe the process of engaging clients across the public sector in presentations demonstrating the benefits of a range of strategic projects designed to ensure cost savings and value for money for government procurement	Engage clients across the public sector in presentations demonstrating the benefits of a range of strategic projects designed to ensure cost savings and value for money for government procurement	Review and implement the process of engaging clients across the public sector in presentations demonstrating the benefits of a range of strategic projects designed to ensure cost savings and value for money for government procurement
2.4 Undertake contract management and risk						
2.4.1 Place commodity orders	Obtain authorisations to administer the contracts	Awareness that authorisations are required to administer the contracts	Understand that authorisations are required to administer the contracts	Obtain authorisations to administer the contracts	Analyse the process in which authorisations are required in order to administer contracts	Develop the process of authorisation in order to administer contracts
	Confirm the type of supplies required	Awareness of the type of supplies required	Understand the type of supplies required	Describe the type of supplies required	Confirm the type of supplies required	Strategically analyse the type of supplies required

COMPETENCY	BEHAVIOURAL COMPETENCIES	Associate	Practitioner	Specialist	Professional	Leader
2.4.1 Place commodity orders	Obtain reference sources and supplier catalogues to clarify required product and its availability	Awareness of the process of obtaining reference sources and supplier catalogues to clarify required product and its availability	Understand the process of obtaining reference sources and supplier catalogues to clarify required product and its availability	Obtain reference sources and supplier catalogues to clarify required product and its availability	Determine the process of obtaining reference sources and supplier catalogues to clarify required product and its availability	Review and implement the process of obtaining reference sources and supplier catalogues to clarify required product and its availability
	Comply with policies and procedures to order required stock and equipment	Awareness of the need to comply with policies and procedures to order required stock and equipment	Understand the process to comply with policies and procedures to order required stock and equipment	Comply with policies and procedures to order required stock and equipment	Ensure compliance with policies and procedures to order required stock and equipment	Develop the process of compliance with policies and procedures to order required stock and equipment
	Confirm the orders with suppliers	Awareness of the process of confirming the orders with suppliers	Understand the process of confirming the orders with suppliers	Confirm the orders with suppliers	Evaluate the process of confirming the orders with suppliers	Review and implement the process of confirming the orders with suppliers
	Identify any problems in placing orders	Awareness that any problems in placing orders need to be identified	Understand the process of identifying any problems in placing orders	Identify any problems in placing orders	Analyse the process of identifying any problems in placing orders	Review and implement the process of identifying any problems in placing orders
	Comply with procedures for placing orders	Awareness of the procedures for placing orders	Understand the procedures for placing orders	Comply with procedures for placing orders	Ensure adherence to the procedures for placing orders	Develop procedures for placing orders
	Identify any problems with order delivery and recommend options for progressing order delivery	Awareness of how to identify any problems with order delivery and recommended options for progressing order delivery	Examine any problems with order delivery and compare recommended options for progressing order delivery	Identify any problems with order delivery and recommend options for progressing order delivery	Evaluate any problems with order delivery and recommended options for progressing order delivery	Strategically analyse any problems with order delivery and recommended options for progressing order delivery
	Ensure effective mechanisms for checking invoices exist and take action to adjust payments accordingly	Awareness of effective mechanisms for checking invoices exist and take action to adjust payments accordingly	Enable the execution of effective mechanisms for checking invoices exist and take action to adjust payments accordingly	Ensure effective mechanisms for checking invoices exist and take action to adjust payments accordingly	Analyse effective mechanisms for checking invoices exist and take action to adjust payments accordingly	Design effective mechanisms for checking invoices exist and take action to adjust payments accordingly
	Receive or raise requisitions and arrange purchase orders and payments	Awareness the process of receiving or raising requisitions and arranging purchase orders and payments	Understand the process of receiving or raising requisitions and arranging purchase orders and payments	Receive or raise requisitions and arrange purchase orders and payments	Analyse the process of receiving or raising requisitions and arranging purchase orders and payments	Review and implement the process of receiving or raising requisitions and arranging purchase orders and payments
2.4.2 Manage contracts	Conduct reviews of performance against agreed key performance indicators	Awareness of the process of conducting reviews of performance against agreed key performance indicators	Understand the process of conducting reviews of performance against agreed key performance indicators	Conduct reviews of performance against agreed key performance indicators	Analyse the process of conducting reviews of performance against agreed key performance indicators	Review and implement the process of conducting reviews of performance against agreed key performance indicators
	Provide timely and expert guidance to remediate procurement performance issues	Awareness of the need for providing timely and expert guidance to remediate procurement performance issues	Understand the need for providing timely and expert guidance to remediate procurement performance issues	Provide timely and expert guidance to remediate procurement performance issues	Evaluate the process of providing timely and expert guidance to remediate procurement performance issues	Strategically analyse the process of providing timely and expert guidance to remediate procurement performance issues

COMPETENCY	BEHAVIOURAL COMPETENCIES	Associate	Practitioner	Specialist	Professional	Leader
2.4.2 Manage contracts (cont.)	Evaluate contract responsiveness and negotiate changes to a contract (contract variations)	Awareness of contract responsiveness and negotiating changes to a contract (contract variations)	Compare contract responsiveness and negotiating changes to a contract (contract variations)	Describe contract responsiveness and negotiate changes to a contract (contract variations)	Evaluate contract responsiveness and negotiate changes to a contract (contract variations)	Strategically analyse contract responsiveness and negotiate changes to a contract (contract variations)
	Manage contract disputes and terminate contracts legally and appropriately if necessary	Awareness of managing contract disputes and the process of terminating contracts legally and appropriately if necessary	Understand the process of managing contract disputes and terminating contracts legally and appropriately if necessary	Manage contract disputes and terminate contracts legally and appropriately if necessary	Evaluate the process of managing contract disputes and terminating contracts legally and appropriately if necessary	Review the process of managing contract disputes and terminating contracts legally and appropriately if necessary
	Describe the importance of supplier control mechanisms, contract administration and supplier management	Awareness of the importance of supplier control mechanisms, contract administration and supplier management	Understand the importance of supplier control mechanisms, contract administration and supplier management	Describe the importance of supplier control mechanisms, contract administration and supplier management	Demonstrate knowledge on the importance of supplier control mechanisms, contract administration and supplier management	Demonstrate in-depth knowledge on the importance of supplier control mechanisms, contract administration and supplier management
2.4.3 Addresses risk and ensures quality management	Provide risk assessments for the procurement process (technical, commercial, administrative)	Awareness of risk assessments for the procurement process (technical, commercial, administrative)	Understand the risk assessments for the procurement process (technical, commercial, administrative)	Describe the process of providing risk assessments for the procurement process (technical, commercial, administrative)	Provide risk assessments for the procurement process (technical, commercial, administrative)	Review and implement the process of providing risk assessments for the procurement process (technical, commercial, administrative)
	Use key performance indicators for assessing procurement and supplier performance and effectiveness	Awareness of the key performance indicators for assessing procurement and supplier performance and effectiveness	Understand the key performance indicators for assessing procurement and supplier performance and effectiveness	Use key performance indicators for assessing procurement and supplier performance and effectiveness	Analyse the key performance indicators for assessing procurement and supplier performance and effectiveness	Develop the key performance indicators for assessing procurement and supplier performance and effectiveness
	Identify any problems with the procurement of supplies in the supply chain	Awareness that problems with the procurement of supplies in the supply chain exist	Examine any problems with the procurement of supplies in the supply chain	Identify any problems with the procurement of supplies in the supply chain	Determine any problems with the procurement of supplies in the supply chain	Strategically analyse any problems with the procurement of supplies in the supply chain
	Develop and implement contingency plan for shortages	Awareness of the contingency plan for shortages	Understand the contingency plan for shortages	Identify the contingency plan for shortages	Evaluate the contingency plan for shortages	Develop and implement contingency plan for shortages
	Recommend options for improving the performance of suppliers	Awareness of the options for improving the performance of suppliers	Compare recommended options for improving the performance of suppliers	Describe recommended options for improving the performance of suppliers	Analyse recommended options for improving the performance of suppliers	Recommend options for improving the performance of suppliers
	Understand procurement portfolio analysis and risk assessment (supply positioning)	Awareness of procurement portfolio analysis and risk assessment (supply positioning)	Understand procurement portfolio analysis and risk assessment (supply positioning)	Apply procurement portfolio analysis and risk assessment (supply positioning)	Demonstrate procurement portfolio analysis and risk assessment (supply positioning)	Implement procurement portfolio analysis and risk assessment (supply positioning)
	Understand the fundamentals of risk planning and assessment	Awareness of risk planning and assessment	Understand the fundamentals of risk planning and assessment	Describe the fundamentals of risk planning and assessment	Demonstrate the fundamentals of risk planning and assessment	Implement the fundamentals of risk planning and assessment

COMPETENCY	BEHAVIOURAL COMPETENCIES	Associate	Practitioner	Specialist	Professional	Leader
2.5 Ensure quality of products	Use and monitor the processes for prequalification of suppliers	Basic operational knowledge on how to use and monitor the process for prequalification of suppliers	Understand the process of using and monitoring for prequalification of suppliers	Use and monitor the processes for prequalification of suppliers	Analyse the use and monitoring process for prequalification of suppliers	Direct the use and monitoring process for prequalification of suppliers
	Ensure health commodities are not counterfeit and meet quality standards	Execute the process of ensuring that health commodities are not counterfeit and meet quality standards	Understand the process of ensuring that health commodities are not counterfeit and meet quality standards	Ensure health commodities are not counterfeit and meet quality standards	Evaluate the process of ensuring that health commodities are not counterfeit and meet quality standards	Develop and implement the process of ensuring that health commodities are not counterfeit and meet quality standards
	Implement, conduct and maintain a reporting system of pharmacovigilance (e.g. report adverse drug reactions) and equipment faults	Awareness of the process of implementing, conducting and maintaining a reporting system of pharmacovigilance (e.g. report adverse drug reactions) and equipment faults	Understand the process of implementing, conducting and maintaining a reporting system of pharmacovigilance (e.g. report adverse drug reactions) and equipment faults	Describe the process of implementing, conducting and maintaining a reporting system of pharmacovigilance (e.g. report adverse drug reactions) and equipment faults	Implement, conduct and maintain a reporting system of pharmacovigilance (e.g. report adverse drug reactions) and equipment faults	Direct the process of implementing, conducting and maintaining a reporting system of pharmacovigilance (e.g. report adverse drug reactions) and equipment faults
	Inspect products when delivered and during storage to catch defects or problems before they are given to, or needed by, clients	Inspect products when delivered and during storage to catch defects or problems before they are given to, or needed by, clients	Examine the process of inspecting products when delivered and during storage to catch defects or problems before they are given to, or needed by, clients	Describe the process of inspecting products when delivered and during storage to catch defects or problems before they are given to, or needed by, clients	Analyse the process of inspecting products when delivered and during storage to catch defects or problems before they are given to, or needed by, clients	Review and Implement the process of inspecting products when delivered and during storage to catch defects or problems before they are given to, or needed by, clients
2.6 Manage import and export of products	Comply with import and export procedures and requirements	Awareness that there is a need to comply with import and export procedures and requirements	Examine compliance with import and export procedures and requirements	Comply with import and export procedures and requirements	Ensure adherence to the import and export procedures and requirements	Implement processes that ensure compliance for import and export procedures and requirements
	Identify import or export requirements for the supplies	Awareness of import or export requirements for the supplies	Compare import or export requirements for the supplies	Identify import or export requirements for the supplies	Determine import or export requirements for the supplies	Develop import or export requirements for the supplies
	Complete import or export documentation for the supplies	Complete import or export documentation for the supplies	Examine import or export documentation for the supplies	Describe import or export documentation for the supplies	Evaluate import or export documentation for the supplies	Strategically analyse import or export documentation for the supplies
	Monitor the completion of import and export procedures	Execute the completion of import and export procedures	Examine the completion of import and export procedures	Describe the completion of import and export procedures	Evaluate the import and export procedures	Develop the import and export procedures
	Identify any problems with the import and export procedures and requirements	Awareness of the importance to identify any problems with the import and export procedures and requirements	Understand the importance to identify any problems with the import and export procedures and requirements	Identify any problems with the import and export procedures and requirements	Evaluate any problems with the import and export procedures and requirements	Strategically analyse any problems with the import and export procedures and requirements
	Demonstrate knowledge about international business processes (e.g. shipping, air cargo, clearing and forwarding, financial transactions, business ethics)	Basic operational knowledge about international business processes (e.g. shipping, air cargo, clearing and forwarding, financial transactions, business ethics)	Understand international business processes (e.g. shipping, air cargo, clearing and forwarding, financial transactions, business ethics)	Apply knowledge about international business processes (e.g. shipping, air cargo, clearing and forwarding, financial transactions, business ethics)	Demonstrate knowledge about international business processes (e.g. shipping, air cargo, clearing and forwarding, financial transactions, business ethics)	Demonstrate in-depth knowledge about international business processes (e.g. shipping, air cargo, clearing and forwarding, financial transactions, business ethics)

COMPETENCY	BEHAVIOURAL COMPETENCIES	Associate	Practitioner	Specialist	Professional	Leader
2.7 Manage donations of products	Follow the national donations policy, referring to the appropriate Ministry for advice	Awareness of the national donations policy, referring to the appropriate Ministry for advice	Understand the national donations policy, referring to the appropriate Ministry for advice	Describe the national donations policy, referring to the appropriate Ministry for advice	Follow the national donations policy, referring to the appropriate Ministry for advice	Ensure adherence to the national donations policy, referring to the appropriate Ministry for advice
	Demonstrate the ability to say no to donations that are not consistent with national donation policy	Awareness to say no to donations that are not consistent with national donation policy	Understand to say no to donations that are not consistent with national donation policy	Demonstrate the ability to say no to donations that are not consistent with national donation policy	Support policies and procedures that enable the ability to say no to donations that are not consistent with national donation policy	Support policies and procedures that enable the ability to say no to donations that are not consistent with national donation policy
2.8 Prepare for product supply during disasters and emergencies	Communicate with the national disaster planning team	Awareness of the national disaster planning team	Understand the need to communicate with the national disaster planning team	Communicate with the national disaster planning team	Analyse communications with the national disaster planning team	Develop the methodology to communicate with the national disaster planning team
	Describe the procurement and logistic requirements for emergency and disaster supply	Awareness of the procurement and logistic requirements for emergency and disaster supply	Understand the procurement and logistic requirements for emergency and disaster supply	Describe the procurement and logistic requirements for emergency and disaster supply	Analyse the procurement and logistic requirements for emergency and disaster supply	Develop the procurement and logistic requirements for emergency and disaster supply
	Describe the importance of the assessment of local capacity before the emergency or disaster supply	Awareness of the importance of the assessment of local capacity before the emergency or disaster supply	Understand the importance of the assessment of local capacity before the emergency or disaster supply	Describe the importance of the assessment of local capacity before the emergency or disaster supply	Assess the local capacity before the emergency or disaster supply	Develop the methodology for assessment of local capacity before the emergency or disaster supply
	Describe the factors that may restrict emergency or disaster supply to take place	Awareness of the factors that may restrict emergency or disaster supply to take place	Understand the factors that may restrict emergency or disaster supply to take place	Describe the factors that may restrict emergency or disaster supply to take place	Analyse the factors that may restrict emergency or disaster supply to take place	Strategically analyse the factors that may restrict emergency or disaster supply to take place
2.9 Undertake or manage manufacturing or compounding of products	Compound under the good manufacturing practice for pharmaceutical (GMP) medicine	Awareness of the good manufacturing practice for pharmaceutical (GMP) medicine	Understand the good manufacturing practice for pharmaceutical (GMP) medicine	Compound under the good manufacturing practice for pharmaceutical (GMP) medicine	Ensure adherence to the good manufacturing practice for pharmaceutical (GMP) medicine when compounding	Implement processes to ensure compounding complies with good manufacturing practice for pharmaceutical (GMP) medicine

3. Storage

COMPETENCY	BEHAVIOURAL COMPETENCIES	Associate	Practitioner	Specialist	Professional	Leader
3.1 Undertake storage, warehousing and inventory management	Distribute products among suppliers, distribution centres, warehouses and customers through a logistics network	Execute distribution of products among suppliers, distribution centres, warehouses and customers through a logistics network	Enable distribution of products among suppliers, distribution centres, warehouses and customers through a logistics network	Efficiently distribute products among suppliers, distribution centres, warehouses and customers through a logistics network	Analyse methods to distribute products among suppliers, distribution centres, warehouses and customers through a logistics network	Develop methods to efficiently distribute products among suppliers, distribution centres, warehouses and customers through a logistics network

COMPETENCY	BEHAVIOURAL COMPETENCIES	Associate	Practitioner	Specialist	Professional	Leader
3.1 Undertake storage, warehousing and inventory management (cont.)	Apply a total systems approach to designing and managing the entire flow of information, materials and services	Be aware of a total systems approach to designing and managing the entire flow of information, materials, and services	Enable a total systems approach to designing and managing the entire flow of information, materials, and services	Identify inputs to a total systems approach to designing and managing the entire flow of information, materials, and services	Analyse a total systems approach to designing and managing the entire flow of information, materials, and services	Develop a total systems approach to designing and managing the entire flow of information, materials, and services
	Justify the choice of equipment or materials based on suitability for intended use, accuracy, safety of use and cost	Awareness of the choice of equipment or materials based on suitability for intended use, accuracy, safety of use and cost	Understand the choice of equipment or materials based on suitability for intended use, accuracy, safety of use and cost	Describe the choice of equipment or materials based on suitability for intended use, accuracy, safety of use and cost	Determine the choice of equipment or materials based on suitability for intended use, accuracy, safety of use and cost	Strategically analyse the choice of equipment or materials based on suitability for intended use, accuracy, safety of use and cost
	Configure warehouses to have formal storage locations that identify the row, rack section, level and shelf location, typically with an alphanumeric location bar code or label	Awareness of the formal storage locations in the warehouse that identify the row, rack section, level and shelf location, typically with an alphanumeric location bar code or label	Understand the importance of warehouses to have formal storage locations that identify the row, rack section, level and shelf location, typically with an alphanumeric location bar code or label	Maintain warehouses formal storage locations that identify the row, rack section, level and shelf location, typically with an alphanumeric location bar code or label	Analyse the warehouse configuration to have formal storage locations that identify the row, rack section, level and shelf location, typically with an alphanumeric location bar code or label	Define rules to configure warehouses to have formal storage locations that identify the row, rack section, level and shelf location, typically with an alphanumeric location bar code or label
	Store medicines appropriately, considering temperature, access and cleanliness	Store medicines appropriately, considering temperature, access and cleanliness	Understand that medicines are to be stored appropriately, considering temperature, access and cleanliness	Describe how medicines are appropriately stored, considering temperature, access and cleanliness	Evaluate how medicines are appropriately stored, considering temperature, access and cleanliness	Develop rules for how medicines are appropriately stored, considering temperature, access and cleanliness
	Demonstrate appropriate use of, and ability to maintain, the cold chain	Ability to maintain the cold chain	Enable use of, and ability to maintain, the cold chain	Identify appropriate use of, and ability to maintain, the cold chain	Determine the appropriate use of, and ability to maintain, the cold chain	Develop rules for the appropriate use of, and ability to maintain, the cold chain
	Control and monitor the movement and storage of materials within a warehouse	Execute the movement and storage of materials within a warehouse	Understand the movement and storage of materials within a warehouse	Describe the movement and storage of materials within a warehouse	Analyse the movement and storage of materials within a warehouse	Develop rules for the movement and storage of materials within a warehouse
	Secure the medical store and limit access to staff	Be aware of the need to secure the medical store and limit access to staff	Understand the need to secure the medical store and limit access to staff	Describe how to secure the medical store and limit access to staff	Analyse the process of securing the medical store and limiting access to staff	Develop methods to secure the medical store and limit access to staff
	Develop and follow organisation processes, instructions, rules and parameters for warehouse and inventory management and use of equipment (e.g. annual stock turns, expiry date tracking, stock procurement, rotation and retrieval, equipment maintenance)	Follow organisation processes, instructions, rules and parameters for warehouse and inventory management and use of equipment (e.g. annual stock turns, expiry date tracking, stock procurement, rotation and retrieval, equipment maintenance)	Understand and follow organisation processes, instructions, rules and parameters for warehouse and inventory management and use of equipment (e.g. annual stock turns, expiry date tracking, stock procurement, rotation and retrieval, equipment maintenance)	Describe organisation processes, instructions, rules and parameters for warehouse and inventory management and use of equipment (e.g. annual stock turns, expiry date tracking, stock procurement, rotation and retrieval, equipment maintenance)	Analyse organisation processes, instructions, rules and parameters for warehouse and inventory management and use of equipment (e.g. annual stock turns, expiry date tracking, stock procurement, rotation and retrieval, equipment maintenance)	Develop organisation processes, instructions, rules and parameters for warehouse and inventory management and use of equipment (e.g. annual stock turns, expiry date tracking, stock procurement, rotation and retrieval, equipment maintenance)

COMPETENCY	BEHAVIOURAL COMPETENCIES	Associate	Practitioner	Specialist	Professional	Leader
3.1 Undertake storage, warehousing and inventory management (cont.)	Develop policies and procedures for stock handling, distribution and withdrawal consistent with maintaining safety (e.g. policies for distribution of concentrated electrolyte solutions and cytotoxics, identification of cytotoxic drug products within the work environment, separation of like-named or like-packaged products)	Awareness of policies and procedures for stock handling, distribution and withdrawal consistent with maintaining safety (e.g. policies for distribution of concentrated electrolyte solutions and cytotoxics, identification of cytotoxic drug products within the work environment, separation of like-named or like-packaged products)	Enable and understand policies and procedures for stock handling, distribution and withdrawal consistent with maintaining safety (e.g. policies for distribution of concentrated electrolyte solutions and cytotoxics, identification of cytotoxic drug products within the work environment, separation of like-named or like-packaged products)	Describe policies and procedures for stock handling, distribution and withdrawal consistent with maintaining safety (e.g. policies for distribution of concentrated electrolyte solutions and cytotoxics, identification of cytotoxic drug products within the work environment, separation of like-named or like-packaged products)	Analyse policies and procedures for stock handling, distribution and withdrawal consistent with maintaining safety (e.g. policies for distribution of concentrated electrolyte solutions and cytotoxics, identification of cytotoxic drug products within the work environment, separation of like-named or like-packaged products)	Develop policies and procedures for stock handling, distribution and withdrawal consistent with maintaining safety (e.g. policies for distribution of concentrated electrolyte solutions and cytotoxics, identification of cytotoxic drug products within the work environment, separation of like-named or like-packaged products)
	Confirm information on the supplies being stored	Confirm information on the supplies being stored	Understand the information on the supplies being stored	Identify requirements of information on the supplies being stored	Analyse requirements for information on the supplies being stored	Develop a standard for information on the supplies being stored
	Consolidate several items into larger units for fewer handlings	Awareness that one must consolidate several items into larger units for fewer handlings	Enable the process of consolidating several items into larger units for fewer handlings	Describe the process of consolidating several items into larger units for fewer handlings	Analyse processes to consolidate several items into larger units for fewer handlings	Develop processes to consolidate several items into larger units for fewer handlings
	Apply methods of stock rotation (e.g. first in first out, FIFO, or first to expire first out, FEFO)	Awareness of methods of stock rotation (e.g. first in first out, FIFO, or first to expire first out, FEFO)	Enable methods of stock rotation (e.g. first in first out, FIFO, or first to expire first out, FEFO)	Describe methods of stock rotation (e.g. first in first out, FIFO, or first to expire first out, FEFO)	Analyse methods of stock rotation (e.g. first in first out, FIFO, or first to expire first out, FEFO)	Review and Implement methods of stock rotation (e.g. first in first out, FIFO, or first to expire first out, FEFO)
	Enter the quantity received and update the warehouse/ inventory/LMIS system software with the stock on hand when each order is delivered according to requisite SOP's	Enter the quantity received and update the warehouse/ inventory/LMIS system software with the stock on hand when each order is delivered according to requisite SOP's	Understand the process of entering the quantity received and update the warehouse/ inventory/LMIS system software with the stock on hand when each order is delivered according to requisite SOP's	Describe the process of entering the quantity received, and updating the warehouse management system software with the stock on hand when each order is delivered	Analyse the process of entering the quantity received and updating the warehouse management system software with the stock on hand when each order is delivered	Develop a process on how to enter the quantity received and update the warehouse management system software with the stock on hand when each order is delivered
	Asses required levels of stock and/ or equipment in specific situations	Awareness that there are required levels of stock and/ or equipment in specific situations	Understand that there are required levels of stock and/or equipment in specific situations	Asses required levels of stock and/ or equipment in specific situations	Determine required levels of stock and/ or equipment in specific situations	Define policies for the assessment of the required levels of stock and/ or equipment in specific situations
	Ensure accurate verification of rolling stocks	Awareness of the importance of accurate verification of rolling stocks	Understand the importance of accurate verification of rolling stocks	Ensure accurate verification of rolling stocks	Measure the accuracy of verification of rolling stocks	Develop processes that accurately verify rolling stocks
	Monitor the location and condition of the supplies being stored	Awareness of the importance of monitoring the location and condition of the supplies being stored	Enable the process of monitoring the location and condition of the supplies being stored	Monitor the location and condition of the supplies being stored	Implement the methodology for monitoring the location and condition of the supplies being stored	Develop a methodology for monitoring the location and condition of the supplies being stored
	Analyse information on the storage locations and facilities	Awareness of the importance of the information on the storage locations and facilities	Understand the importance of the information on the storage locations and facilities	Collate and prepare information on the storage locations and facilities	Analyse information on the storage locations and facilities	Develop solutions with the information on the storage locations and facilities

COMPETENCY	BEHAVIOURAL COMPETENCIES	Associate	Practitioner	Specialist	Professional	Leader
3.1.1 Manage storage of commodities during emergency	Use collated data to identify any problems in using the storage locations and facilities	Awareness of the need to communicate any problems in using the storage locations and facilities	Examine any problems in using the storage locations and facilities	Identify any problems in using the storage locations and facilities	Analyse any problems in using the storage locations and facilities	Develop strategies for the resolution of problems in using the storage locations and facilities
	Describe the types of warehouses available for emergency or disaster supply	Awareness of the types of warehouses available for emergency or disaster supply	Understand the types of warehouses with those available for emergency or disaster supply	Describe the types of warehouses available for emergency or disaster supply	Analyse the different types of warehouses available for emergency or disaster supply	Direct processes for the different types of warehouses available for emergency or disaster supply

COMPETENCY	BEHAVIOURAL COMPETENCIES	Associate	Practitioner	Specialist	Professional	Leader
3.2 Supply commodities to facilities	Describe the importance of the choice of storage site for emergency or disaster supply	Awareness of the choice of storage site for emergency or disaster supply	Understanding the importance of the choice of storage site for emergency or disaster supply	Describe the importance of the choice of storage site for emergency or disaster supply	Analyse the choice of storage site for emergency or disaster supply	Directs the choice of storage site for emergency or disaster supply
	Describe the staff required for the storage of emergency or disaster supply	Awareness of the staff required for the storage of emergency or disaster supply	Understanding of the staff required for the storage of emergency or disaster supply	Describe the staff required for the storage of emergency or disaster supply	Evaluate the staff required for the storage of emergency or disaster supply	Direct the staff required for the storage of emergency or disaster supply
	Describe the equipment and material required for the warehousing of emergency or disaster supply	Awareness of the equipment and material required for the warehousing of emergency or disaster supply	Compare the equipment and material required for the warehousing of emergency or disaster supply	Describe the equipment and material required for the warehousing of emergency or disaster supply	Determine the equipment and material required for the warehousing of emergency or disaster supply	Strategically analyse the equipment and material required for the warehousing of emergency or disaster supply
	Conduct a rapid assessment of logistical needs in emergencies	Awareness of the need for a rapid assessment of logistical needs in emergencies	Enable the conduct of a rapid assessment of logistical needs in emergencies	Provide input to a rapid assessment of logistical needs in emergencies	Conduct a rapid assessment of logistical needs in emergencies	Develop methods on how to conduct rapid assessments of logistical needs in emergencies
	List the sources of distribution demand (customers)	Working knowledge of the various sources of distribution demand (customers)	List the sources of distribution demand (customers)	Describe the various sources of distribution demand (customers)	Determine the sources of distribution demand (customers)	Strategically analyse the sources of distribution demand (customers)
	Demonstrate use of order policies for planned order generation	Basic operational knowledge of order policies for planned order generation	Understand the use of order policies for planned order generation	Describe the use of order policies for planned order generation	Demonstrate the use of order policies for planned order generation	Develop the order policies for planned order generation
	Calculate re-supply quantities using a variety of inventory methods, including visual review, two bin, periodic review, order point and just in time	Execute re-supply quantities using a variety of inventory methods, including visual review, two bin, periodic review, order point and just in time	Explain the calculation of re-supply quantities using a variety of inventory methods, including visual review, two bin, periodic review, order point and just in time	Describe the calculation of re-supply quantities using a variety of inventory methods, including visual review, two bin, periodic review, order point and just in time	Calculate re-supply quantities using a variety of inventory methods, including visual review, two bin, periodic review, order point and just in time	Design calculation to re-supply quantities that use a variety of inventory methods, including visual review, two bin, periodic review, order point and just in time
	Prepare and use order schedules	Basic knowledge of the use of order schedules	Use order schedules	Prepare and use order schedules	Evaluate the process of preparing and using order schedules	Develop and implement the process of preparing and using order schedules
	Calculate safety stock	Basic operational knowledge of safety stocks	Understand safety stock calculations	Calculate safety stock	Evaluate safety stock calculations	Develop safety stock calculations
	Use economic order quantity (EOQ) principles in ordering	Awareness of the principles of EOQ in the ordering process	Understand the principles of EOQ in the ordering process	Use economic order quantity (EOQ) principles in ordering	Analyse the use of economic order quantity (EOQ) principles in ordering	Develop parameters for the use of economic order quantity (EOQ) principles in ordering

COMPETENCY	BEHAVIOURAL COMPETENCIES	Associate	Practitioner	Specialist	Professional	Leader
3.2 Supply commodities to facilities (cont.)	Demonstrate the use of pull and push inventory control systems	Awareness of pull and push inventory control systems	Understand the use of pull and push inventory control systems	Apply the use of pull and push inventory control systems	Evaluate the effectiveness of using pull and push inventory control systems	Develop the methodology for using of pull and push inventory control systems
	Promote a regular, rather than urgent, order culture	Execute a regular, rather than urgent, order culture	Enable a regular, rather than urgent, order culture	Apply a regular, rather than urgent, order culture	Determine a regular, rather than urgent, order culture	Promote a regular, rather than urgent, order culture
	Determine order-filling priorities in relation to delivery opportunities and urgency	Execute order-filling priorities in relation to delivery opportunities and urgency	Enable order-filling priorities in relation to delivery opportunities and urgency	Apply order-filling priorities in relation to delivery opportunities and urgency	Determine order-filling priorities in relation to delivery opportunities and urgency	Lead order-filling priorities in relation to delivery opportunities and urgency
	Screen orders (modify order quantities on the basis of available stock, impact on service delivery, distance of facility from hospital)	Awareness that orders must be screened (modify order quantities on the basis of available stock, impact on service delivery, distance of facility from hospital)	Understand that orders must be screened (modify order quantities on the basis of available stock, impact on service delivery, distance of facility from hospital)	Screen orders (modify order quantities on the basis of available stock, impact on service delivery, distance of facility from hospital)	Analyse the parameters under which one must screen orders (e.g. modify order quantities on the basis of available stock, impact on service delivery, distance of facility from hospital)	Strategically analyse the parameters under which one must screen orders (e.g. modify order quantities on the basis of available stock, impact on service delivery, distance of facility from hospital)
	Assemble, check and pack orders	Assemble, check and pack orders	Enable assembling, checking and packing of orders	Describe assembling, checking and packing of orders	Evaluate assembling, checking and packing of orders	Strategically analyse assembling, checking and packing of orders
	Understand making and using dispatch lists and invoices	Execute dispatch lists and invoices	Understand making and using dispatch lists and invoices	Describe making and using dispatch lists and invoices	Demonstrate making and using dispatch lists and invoices	Direct making and using dispatch lists and invoices
	Balance supply with demand, considering both lead time and demand variability created by supply patterns not matching demand patterns	Awareness of the importance of the balancing of supply with demand, considering both lead time and demand variability created by supply patterns not matching demand patterns	Enable the balancing of supply with demand, considering both lead time and demand variability created by supply patterns not matching demand patterns	Describe the balancing of supply with demand, considering both lead time and demand variability created by supply patterns not matching demand patterns	Balance supply with demand, considering both lead time and demand variability created by supply patterns not matching demand patterns	Review and Implement processes for the balancing of supply with demand, considering both lead time and demand variability created by supply patterns not matching demand patterns
	Analyse information on the storage locations and facilities	Basic operational knowledge on the storage locations and facilities	Understand information on the storage locations and facilities	Describe information on the storage locations and facilities	Analyse information on the storage locations and facilities	Strategically analyse information on the storage locations and facilities
	Measure customer satisfaction and develop loyal customers by using performance metrics taken from the customer perspective, with criteria such as on-time delivery, perception of quality, complaints and length of wait times	Awareness of the measures of customer satisfaction by using performance metrics taken from the customer perspective, with criteria such as on-time delivery, perception of quality, complaints and length of wait times	Understand measures of customer satisfaction by using performance metrics taken from the customer perspective, with criteria such as on-time delivery, perception of quality, complaints and length of wait times	Apply measures of customer satisfaction and identify loyal customers by using performance metrics taken from the customer perspective, with criteria such as on-time delivery, perception of quality, complaints and length of wait times	Measure customer satisfaction and develop loyal customers by using performance metrics taken from the customer perspective, with criteria such as on-time delivery, perception of quality, complaints and length of wait times	Develop measures of customer satisfaction and develop loyal customers by using performance metrics taken from the customer perspective, with criteria such as on-time delivery, perception of quality, complaints and length of wait times

COMPETENCY	BEHAVIOURAL COMPETENCIES	Associate	Practitioner	Specialist	Professional	Leader
**3.3 Supply commodities to sections within a facility**	Establish, review, implement and use an imprest list in conjunction with input from doctors and nurses	Awareness of the use of an imprest list	Understand the use of imprest lists	Review imprest quantities regularly and assist in the development of the list in conjunction with doctors and nurses	Review imprest list and their implementations in the context	Strategically analyse and direct the implementation of imprest lists in the context.
**3.4 Manage transport for commodities**	Manage distribution activities, including a fleet of vehicles and distribution schedules, to deliver health commodities to facilities.	Execute distribution activities, including a fleet of vehicles and distribution schedules, to deliver health commodities to facilities.	Understand distribution activities, including a fleet of vehicles and distribution schedules, to deliver health commodities to facilities.	Manage distribution activities, including a fleet of vehicles and distribution schedules, to deliver health commodities to facilities.	Analyse distribution activities, including a fleet of vehicles and distribution schedules, to deliver health commodities to facilities.	Direct distribution activities, including a fleet of vehicles and distribution schedules, to deliver health commodities to facilities.
	Demonstrate knowledge of policies and procedures related to handling and transporting special products such as vaccines, HIV/AIDS drugs, other high value products and narcotics	Execute the policies and procedures related to handling and transporting special products such as vaccines, HIV/AIDS drugs, other high value products and narcotics	Understand the policies and procedures related to handling and transporting special products such as vaccines, HIV/AIDS drugs, other high value products and narcotics	Apply knowledge of policies and procedures related to handling and transporting special products such as vaccines, HIV/AIDS drugs, other high value products and narcotics	Demonstrate knowledge of policies and procedures related to handling and transporting special products such as vaccines, HIV/AIDS drugs, other high value products and narcotics	Develop policies and procedures related to handling and transporting special products such as vaccines, HIV/AIDS drugs, other high value products and narcotics
	Choose shipping methods, considering trade-offs between costs and benefits	Basic operational knowledge of shipping methods, considering trade-offs between costs and benefits	Understands shipping methods, considering trade-offs between costs and benefits	Choose and compare shipping methods, considering trade-offs between costs and benefits	Analyse shipping methods, considering trade-offs between costs and benefits	Strategically analyse shipping methods, considering trade-offs between costs and benefits
	Understand local and national laws/ requirements for vehicle safety, driving regulations and licensing	Awareness of local and national laws/ requirements for vehicle safety, driving regulations and licensing	Understand local and national laws/ requirements for vehicle safety, driving regulations and licensing	Apply knowledge of all local and national laws/requirements for vehicle safety, driving regulations and licensing	Ensure compliance to all local and national laws/ requirements for vehicle safety, driving regulations and licensing	Understand strategic impact of compliance regarding all local and national laws/ requirements for vehicle safety, driving regulations and licensing
	Identify and implement proper packing and labelling methods for transportation of hazardous materials	Execute proper packing and labelling methods for transportation of hazardous materials	Understand proper packing and labelling methods for transportation of hazardous materials		Evaluate proper packing and labelling methods for transportation of hazardous materials	Review and Implement proper packing and labelling methods for transportation of hazardous materials
	Maximise freight loads while minimizing freight costs	Awareness of the process of maximizing freight loads while minimizing freight costs	Understand the process of maximizing freight loads while minimizing freight costs	Demonstrate maximizing freight loads while minimizing freight costs	Determine how to maximize freight loads while minimizing freight costs	Strategically analyse how to maximize freight loads while minimizing freight costs
	Ensure efficient use of transportation resources while meeting customers' needs	Execute efficient use of transportation resources while meeting customers' needs	Enable efficient use of transportation resources while meeting customers' needs	Describe the efficient use of transportation resources while meeting customers' needs	Determine the efficient use of transportation resources while meeting customers' needs	Illustrate efficient use of transportation resources while meeting customers' needs
	Integrate movement demands with vehicle resources	Execute movement demands with vehicle resources	Examine movement demands with vehicle resources	Integrate movement demands with vehicle resources	Evaluate movement demands with vehicle resources	Direct movement demands with vehicle resources
**3.4 Manage transport for commodities**	Apply quantitative techniques when solving logistics problems, such as designing routes and scheduling vehicles	Basic operational knowledge of quantitative techniques when solving logistics problems, such as designing routes and scheduling vehicles	Understand quantitative techniques when solving logistics problems, such as designing routes and scheduling vehicles	Apply quantitative techniques when solving logistics problems, such as designing routes and scheduling vehicles	Demonstrate quantitative techniques when solving logistics problems, such as designing routes and scheduling vehicles	Develop quantitative techniques for solving logistics problems, such as designing routes and scheduling vehicles

COMPETENCY	BEHAVIOURAL COMPETENCIES	Associate	Practitioner	Specialist	Professional	Leader
**3.4 Manage transport for commodities (cont.)**	Deliver products to hospitals and district health offices with the accompanying dispatch note and report and requisition forms	Execute the delivery of products to hospitals and district health offices with the accompanying dispatch note and report and requisition forms	Understand the process of delivering products to hospitals and district health offices with the accompanying dispatch note and report and requisition forms	Describe the process of delivering products to hospitals and district health offices with the accompanying dispatch note and report and requisition forms	Evaluate the delivery of products to hospitals and district health offices with the accompanying dispatch note and report and requisition forms	Strategically analyse the delivery of products to hospitals and district health offices with the accompanying dispatch note and report and requisition forms
	Coordinate transport options for order delivery	Execute transport options for order delivery	Coordinate transport options for order delivery	Describe transport options for order delivery	Determine transport options for order delivery	Formulate transport options for order delivery
	Ensure vehicle availability through developing and implementing a vehicle maintenance plan	Awareness that vehicle availability is due to a vehicle maintenance plan	Understand that vehicle availability is due to a vehicle maintenance plan	Ensure vehicle availability through developing and implementing a vehicle maintenance plan	Analysing the requirements of a vehicle maintenance plan	Develop a system for implementing a vehicle maintenance plan
	Collect and analyse information on distribution requirements	List information on distribution requirements	Interpret information on distribution requirements	Describe information on distribution requirements	Collect and analyse information on distribution requirements	Synthesize information on distribution requirements
	Identify any problems in distribution requirements	Awareness of any problems in distribution requirements	Examine any problems in distribution requirements	Identify any problems in distribution requirements	Resolve any problems in distribution requirements	Strategically analyse any problems in distribution requirements
	Equip distribution sites	Execute the process of equipping distribution sites	Enable the process of equipping distribution sites	Integrate the process of equipping distribution sites	Analyse the process of equipping distribution sites	Develop the process of equipping distribution sites
	Inform stakeholders for better coordination of distribution	Awareness of the importance of informing stakeholders for better coordination of distribution	Understand the need to inform stakeholders for better coordination of distribution	Inform stakeholders for better coordination of distribution	Interpret and coordinate the information provided to stakeholders for better coordination of distribution	Strategically analyse what information needs to be provided to stakeholders for better coordination of distribution
	Implement tracking devices (tools and indicators)	Awareness of tracking devices (tools and indicators)	Compare tracking devices (tools and indicators)	Integrate tracking devices (tools and indicators)	Evaluate tracking devices (tools and indicators)	Review and Implement tracking devices (tools and indicators)
**3.4.1 Manage transport for commodities during disaster**	List knowledge of the different types of transport and their characteristics for emergency or disaster supply	List knowledge of the different types of transport and their characteristics for emergency or disaster supply	Be able to compare of the different types of transport and their characteristics for emergency or disaster supply	Describe the different types of transport and their characteristics for emergency or disaster supply	Evaluate applicability of each of the different types of transport and their characteristics for emergency or disaster supply	Determine which of the different types of transport and their characteristics should be used for emergency or disaster supply
**3.5 Manage disposal of products (e.g. expired, damaged, redundant products)**						
**3.5.1 Define and direct process for managing redundant and returned stock**	Confirm information on the supplies being returned	Take down information on the supplies being returned.	Understand information on the supplies being returned	Confirm information on the supplies being returned	Evaluate information on the supplies being returned	Strategically analyse information on the supplies being returned
	Collate, prepare and analyse data on the flow of returned supplies	Basic operational knowledge of the flow of returned supplies	Understand the flow of returned supplies	Describe the flow of returned supplies	Collate, prepare and analyse data on the flow of returned supplies	In-depth knowledge on the flow of returned supplies
	Identify any problems with the flow of returned supplies	Awareness of any problems with the flow of returned supplies	Interpret any problems with the flow of returned supplies	Identify any problems with the flow of returned supplies	Resolve any problems with the flow of returned supplies	Strategically analyse any problems with the flow of returned supplies

COMPETENCY	BEHAVIOURAL COMPETENCIES	Associate	Practitioner	Specialist	Professional	Leader
3.5.1 Define and direct process for managing redundant and returned stock (cont.)	Recommend options for improving the flow of returned supplies	Awareness of the various options for improving the flow of returned supplies	Compare options for improving the flow of returned supplies	Integrate options for improving the flow of returned supplies	Recommend options for improving the flow of returned supplies	Develop options for improving the flow of returned supplies
	Comply with procedures for monitoring the flow of returned supplies	Awareness of procedures for monitoring the flow of returned supplies	Understand procedures for monitoring the flow of returned supplies	Apply procedures for monitoring the flow of returned supplies	Analyse procedures for monitoring the flow of returned supplies	Develop procedures for monitoring the flow of returned supplies
	Describe and/or demonstrate recall procedures to be used in response to a product recall notice or to access the information promptly.	Awareness of the recall procedures to be used in response to a product recall notice or to access the information promptly.	Understand the recall procedures to be used in response to a product recall notice or to access the information promptly.	Describe and/or demonstrate recall procedures to be used in response to a product recall notice or to access the information promptly.	Determine recall procedures to be used in response to a product recall notice or to access the information promptly.	Develop recall procedures to be used in response to a product recall notice or to access the information promptly.
	Identify issues relevant to the urgency and scope of action required in response to a product recall notice	Execute action required in response to a product recall notice	Understand issues relevant to the urgency and scope of action required in response to a product recall notice	Identify issues relevant to the urgency and scope of action required in response to a product recall notice	Demonstrate knowledge of issues relevant to the urgency and scope of action required in response to a product recall notice	Directs issues relevant to the urgency and scope of action required in response to a product recall notice
	Explain the importance of reporting and the procedures for following up on medication incidents	Awareness of reporting and the procedures for following up on medication incidents	Explain the importance of reporting and the procedures for following up on medication incidents	Identify the information required for reporting and procedures with regards to following up on medication incidents	Analyse the information provided in the reports when following up on medication incidents	Develop the process of reporting and the procedures for following up on medication incidents
	Identify follow-up strategies likely to be effective in preventing recurrence (e.g. root cause analysis)	Realize follow-up strategies likely to be effective in preventing recurrence (e.g. root cause analysis)	Understand follow-up strategies likely to be effective in preventing recurrence (e.g. root cause analysis)	Identify follow-up strategies likely to be effective in preventing recurrence (e.g. root cause analysis)	Evaluate follow-up strategies likely to be effective in preventing recurrence (e.g. root cause analysis)	Design follow-up strategies likely to be effective in preventing recurrence (e.g. root cause analysis)
	Develop an integrated healthcare waste management plan	Execute an integrated healthcare waste management plan	Enable an integrated healthcare waste management plan	Describe an integrated healthcare waste management plan	Evaluate an integrated healthcare waste management plan	Develop an integrated healthcare waste management plan
	Follow guides and procedures for collection, sorting, transport and disposal	Follow guides and procedures for collection, sorting, transport and disposal	Enable guides and procedures for collection, sorting, transport and disposal	Describe guides and procedures for collection, sorting, transport and disposal	Evaluate guides and procedures for collection, sorting, transport and disposal	Develop guides and procedures for collection, sorting, transport and disposal
3.5.2 Manage process for disposal of returned stock	Dispose of expired medications and/or medical equipment according to national policy	Execute the disposal of expired medications and/or medical equipment according to national policy	Enable the disposal of expired medications and/or medical equipment according to national policy	Dispose of expired medications and/or medical equipment according to national policy	Analyse the process of disposing of expired medications and/or medical equipment according to national policy	Develop processes of disposing of expired medications and/or medical equipment according to national policy
	Describe and/or use an appropriate recording system for disposal	Awareness of a recording system for disposal	Understand the recording system for disposal	Describe and/or use an appropriate recording system for disposal	Evaluate an appropriate recording system for disposal	Develop an appropriate recording system for disposal
	Dispose of specific individual items of greatest risk with appropriate care (e.g. oncology medicine)	Execute the disposal of specific individual items of greatest risk with appropriate care (e.g. oncology medicine)	Enable the disposal of specific individual items of greatest risk with appropriate care (e.g. oncology medicine)	Describe the disposal of specific individual items of greatest risk with appropriate care (e.g. oncology medicine)	Analyse the process of disposal for specific individual items of greatest risk with appropriate care (e.g. oncology medicine)	Develop the process of disposal for specific individual items of greatest risk with appropriate care (e.g. oncology medicine)

COMPETENCY	BEHAVIOURAL COMPETENCIES	Associate	Practitioner	Specialist	Professional	Leader
3.6 Manage manufacturing or compounding of products	Compound under the good manufacturing practice (GMP) for pharmaceutical medicines	Awareness of good manufacturing practice (GMP) for pharmaceutical medicines	Understand the reason for good manufacturing practice (GMP) for pharmaceutical medicines	Compound under the good manufacturing practice (GMP) for pharmaceutical medicines	Evaluate the applicability of the good manufacturing practice (GMP) for pharmaceutical medicines	Develop processes for complying with good manufacturing practice (GMP) for pharmaceutical medicines
	Identify factors that can affect medicine stability	Awareness of factors that can affect medicine stability	Examine factors that can affect medicine stability	Identify factors that can affect medicine stability	Evaluate factors that can affect medicine stability	Demonstrate knowledge of factors that can affect medicine stability
	Recognise when a product needs to be prepared under sterile or special conditions (e.g., cytotoxic)	Awareness that some products need to be prepared under sterile or special conditions (e.g. cytotoxic)	Understand why some products need to be prepared under sterile or special conditions (e.g. cytotoxic)	Recognise when a product needs to be prepared under sterile or special conditions (e.g. cytotoxic)	Determine when a product needs to be prepared under sterile or special conditions (e.g. cytotoxic)	Develop processes to enable recognition when a product needs to be prepared under sterile or special conditions (e.g. cytotoxic)
	Prepare the formulation worksheet, calculations and labels	Awareness of the formulation worksheet, calculations and labels	Interpret the formulation worksheet, calculations and labels	Prepare the formulation worksheet, calculations and labels	Evaluate the process for the preparation of the formulation worksheet, calculations and labels	Develop the process for the preparation of the formulation worksheet, calculations and labels
	Compound the product using appropriate compounding techniques and principles	Awareness that products must be compounded using appropriate compounding techniques and principles	Understand that products must be compounded using appropriate compounding techniques and principles	Compound the product using appropriate compounding techniques and principles	Evaluate the process of compounding a product using appropriate compounding techniques and principles	Develop the process for the compounding of a product using appropriate compounding techniques and principles
	Comply with legal, workplace and professional requirements when preparing and dispensing compounded products	Awareness of the legal, workplace and professional requirements when preparing and dispensing compounded products	Enable compliance with legal, workplace and professional requirements when preparing and dispensing compounded products	Comply with legal, workplace and professional requirements when preparing and dispensing compounded products	Evaluate legal, workplace and professional requirements when preparing and dispensing compounded products	Develop legal, workplace and professional requirements when preparing and dispensing compounded products
	Pack and label compounded products for safety, stability and patient compliance	Awareness that compounded products must be packed and labelled for safety, stability and patient compliance.	Understand that compounded products must be packed and labelled for safety, stability and patient compliance.	Pack and label compounded products for safety, stability and patient compliance	Evaluate the process of packing and labelling compounded products for safety, stability and patient compliance	Develop the processes of packing and labelling compounded products for safety, stability and patient compliance
	Clean and maintain compounding equipment and area	Awareness that compounding equipment and area must be maintained and cleaned.	Understand that compounding equipment and area must be maintained and cleaned.	Clean and maintain compounding equipment and area	Evaluate the process of cleaning and maintaining compounding equipment and area	Develop the process of cleaning and maintaining compounding equipment and area
	Complete documentation and records related to compounding or product manufacturing	Awareness that complete documentation and records related to compounding or product manufacturing must be kept	Understand that complete documentation and records related to compounding or product manufacturing must be kept	Complete documentation and records related to compounding or product manufacturing	Evaluate the process of completing documentation and records related to compounding or product manufacturing	Develop the process of completing documentation and records related to compounding or product manufacturing
	Schedule production activities (Including issuing, product testing, staging and releasing product)	Execute against the schedule of production activities (Including issuing, product testing, staging and releasing product)	Enable the execution against the schedule of production activities (Including issuing, product testing, staging and releasing product)	Schedule production activities (Including issuing, product testing, staging and releasing product)	Evaluate the scheduling of production activities (Including issuing, product testing, staging and releasing product)	Develop processes for the scheduling of production activities (Including issuing, product testing, staging and releasing product)

COMPETENCY	BEHAVIOURAL COMPETENCIES	Associate	Practitioner	Specialist	Professional	Leader
3.6 Manage manufacturing or compounding of products (cont.)	Manage manufacturing equipment and facilities, including transportation	Awareness that manufacturing equipment and facilities, including transportation need to be managed	Understand that manufacturing equipment and facilities, including transportation need to be managed	Manage manufacturing equipment and facilities, including transportation	Evaluate the management of manufacturing equipment and facilities, including transportation	Develop processes for the management of manufacturing equipment and facilities, including transportation
	Explain the use of compounding techniques and equipment	Awareness of the use of compounding techniques and equipment	Understand the use of compounding techniques and equipment	Explain the use of compounding techniques and equipment	Evaluate the use of compounding techniques and equipment	Develop the use of compounding techniques and equipment
	Explain the role of non-therapeutic agents, such as suspending agents, preservatives, buffers and flavourings.	Awareness of non-therapeutic agents, such as suspending agents, preservatives, buffers and flavourings.	Understand of role of non-therapeutic agents, such as suspending agents, preservatives, buffers and flavourings.	Explain the role of non-therapeutic agents, such as suspending agents, preservatives, buffers and flavourings.	Incorporate the use of non-therapeutic agents, such as suspending agents, preservatives, buffers and flavourings into the role based processes.	Strategically direct the use of non-therapeutic agents, such as suspending agents, preservatives, buffers and flavourings to support the operating.
3.7 Manage re-packing of products	Predict when re-packing or pre-packs are required	Execute re-packing or pre-packing	Understand when re-packing or pre-packs are required	Predict when re-packing or pre-packs are required	Determine when re-packing or pre-packs are required	Strategically analyse and design the process to control when re-packing or pre-packs are required
	Demonstrate a safe system for repacking	Execute a safe system for repacking	Enable a safe system for repacking	Demonstrate a safe system for repacking	Determine a safe system for repacking	Review process for a safe system for repacking
3.8 Customer relationship management	Implementation of different product strategies in line with market and demand source segmentation strategies.	Awareness of the need for different product strategies in line with market and demand source segmentation strategies.	Understand the need for different product strategies in line with market and demand source segmentation strategies.	Identify different product strategies in line with market and demand source segmentation strategies.	Evaluation of different product strategies in line with market and demand source segmentation strategies.	Implementation of different product strategies in line with market and demand source segmentation strategies.
	Establishing relationships with new demand sources and managing relationships with existing demand sources.	Awareness of the need for the establishment of relationships with new demand sources and managing relationships with existing demand sources.	Understand the need for the establishment of relationships with new demand sources and managing relationships with existing demand sources.	Establishing relationships with new demand sources and managing relationships with existing demand sources.	Evaluate the establishment of relationships with new demand sources and managing relationships with existing demand sources.	Design processes for the establishment of relationships with new demand sources and managing relationships with existing demand sources.
	Alignment of demand sources with demand segmentation strategies	Awareness of the alignment of demand sources with demand segmentation strategies	Understand the alignment of demand sources with demand segmentation strategies	Describe the alignment of demand sources with demand segmentation strategies	Alignment of demand sources with demand segmentation strategies	Design standards of alignment of demand sources with demand segmentation strategies

COMPETENCY	BEHAVIOURAL COMPETENCIES	Associate	Practitioner	Specialist	Professional	Leader
3.9 Facility design	Design and layout of the size and configuration of the facility/building.	Awareness of the requirements of the design and layout of the size and configuration of the facility/building.	Understand the requirements of the design and layout of the size and configuration of the facility/building.	Provide inputs to the design and layout of the size and configuration of the facility/building.	Evaluate inputs to design and layout of the size and configuration of the facility/building.	Design and layout of the size and configuration of the facility/building.
	Design the flow of product through the facility to making sure it is achievable with the selection of material handling equipment.	Execute the flow of product through the facility to making sure it is achievable with the selection of material handling equipment	Enable the flow of product through the facility to making sure it is achievable with the selection of material handling equipment	Describe the flow of product through the facility to making sure it is achievable with the selection of material handling equipment	Evaluate the flow of product through the facility to making sure it is achievable with the selection of material handling equipment	Design the flow of product through the facility to making sure it is achievable with the selection of material handling equipment
	Balance the health and safety requirements of the facility with its performance requirements	Awareness of the need to balance the health and safety requirements of the facility with its performance requirements	Enable the achievement balancing the health and safety requirements of the facility with its performance requirements	Achieve the balance of the health and safety requirements of the facility with its performance requirements	Evaluate the achievement of the balance of the health and safety requirements of the facility with its performance requirements	Design processes to the balance of the health and safety requirements of the facility with its performance requirements

4. Use/Dispense

COMPETENCY	BEHAVIOURAL COMPETENCIES	Associate	Practitioner	Specialist	Professional	Leader
4.1 Understand use of medical products (e.g. safety procedures, dispensing protocols, standard treatment/ testing guidelines)	Identify medicines by their generic name	Awareness that medicines must be identifiable by their generic name	Understand that medicines must be identifiable by their generic name	Identify medicines by their generic name	Analyse records to allow identification of medicines by their generic name	Review the process of analysing records to allow identification of medicines by their generic name
	Describe the way medicines work, their use (how much, how often and for how long), and their main adverse effects and cautions	Awareness that the way medicines work, their use (how much, how often and for how long), and their main adverse effects and cautions	Understand the way medicines work, their use (how much, how often and for how long), and their main adverse effects and cautions	Describe the way medicines work, their use (how much, how often and for how long), and their main adverse effects and cautions	Describe the way medicines work, their use (how much, how often and for how long), and their main adverse effects and cautions as well as make recommendation on medications based on availability	Describe the way medicines work, their use (how much, how often and for how long), and their main adverse effects and cautions as well as make recommendation on medications based on availability
	Identify that some signs and symptoms shown by a patient may be the result of adverse medication effects, and these people need to be referred to the appropriate health personnel	Awareness that some signs and symptoms shown by a patient may be the result of adverse medication effects, and these people need to be referred to the appropriate health personnel	Understand that some signs and symptoms shown by a patient may be the result of adverse medication effects, and these people need to be referred to the appropriate health personnel	Identify that some signs and symptoms shown by a patient may be the result of adverse medication effects, and these people need to be referred to the appropriate health personnel and use medicines adverse effect reporting systems	Identify that some signs and symptoms shown by a patient may be the result of adverse medication effects, and these people need to be referred to the appropriate health personnel and use medicines adverse effect reporting systems	Identify that some signs and symptoms shown by a patient may be the result of adverse medication effects, and these people need to be referred to the appropriate health personnel and use medicines adverse effect reporting systems
	Describe how individual pieces of medical equipment are used, noting personal and patient safety	Describe how individual pieces of medical equipment are used, noting personal and patient safety	Describe how individual pieces of medical equipment are used, noting personal and patient safety	Describe how individual pieces of medical equipment are used, noting personal and patient safety	Describe how individual pieces of medical equipment are used, noting personal and patient safety	Describe how individual pieces of medical equipment are used, noting personal and patient safety
	Identify when to dispose of medical equipment or sundries	Awareness of when to dispose of medical equipment or sundries	Understand when to dispose of medical equipment or sundries	Identify when to dispose of medical equipment or sundries	Define processes of identifying when to dispose of medical equipment or sundries	Review processes of identifying when to dispose of medical equipment or sundries

COMPETENCY	BEHAVIOURAL COMPETENCIES	Associate	Practitioner	Specialist	Professional	Leader
4.1 Understand use of medical products (e.g. safety procedures, dispensing protocols, standard treatment/ testing guidelines) (cont.)	Demonstrate to patients how to use any equipment given to them for their care	Awareness that demonstration must be done to patients on how to use any equipment given to them for their care	Understand that demonstration must be done to patients on how to use any equipment given to them for their care	Demonstrate to patients how to use any equipment given to them for their care	Define processes to demonstrate to patients how to use any equipment given to them for their care	Review processes to demonstrate to patients how to use any equipment given to them for their care
	Maintain equipment supplied by the pharmacy and use any existing maintenance support network	Awareness that equipment supplied by the pharmacy must be maintained and use any existing maintenance support network	Understand that equipment supplied by the pharmacy must be maintained and use any existing maintenance support network	Maintain equipment supplied by the pharmacy and use any existing maintenance support network	Define processes to maintain equipment supplied by the pharmacy and use any existing maintenance support network	Review and implement processes to maintain equipment supplied by the pharmacy and use any existing maintenance support network
	Keep up to date with standard treatment guidelines	Awareness of the importance of keeping up to date with standard treatment guidelines	Understand the importance of keeping up to date with standard treatment guidelines	Keep up to date with standard treatment guidelines	Define standard to keep up to date with standard treatment guidelines	Review standard to keep up to date with standard treatment guidelines

COMPETENCY	BEHAVIOURAL COMPETENCIES	Associate	Practitioner	Specialist	Professional	Leader
4.2 Provide information and advice to the product user/patient	Follow country-based treatment guidelines to ensure the appropriate use of medicines	Awareness of the importance of following country-based treatment guidelines to ensure the appropriate use of medicines	Understand the importance of following country-based treatment guidelines to ensure the appropriate use of medicines	Follow country-based treatment guidelines to ensure the appropriate use of medicines	Define country-based treatment guidelines to ensure the appropriate use of medicines	Review country-based treatment guidelines to ensure the appropriate use of medicines
	Provide structured, patient-centred consultation with the consumer/ carer without engendering concern, resistance or other adverse reactions	Awareness of the importance of providing structured, patient-centred consultation with the consumer/ carer without engendering concern, resistance or other adverse reactions	Understand the importance of providing structured, patient-centred consultation with the consumer/ carer without engendering concern, resistance or other adverse reactions	Provide structured, patient-centred consultation with the consumer/ carer without engendering concern, resistance or other adverse reactions	Define processes to provide structured, patient-centred consultation with the consumer/ carer without engendering concern, resistance or other adverse reactions	Review processes to provide structured, patient-centred consultation with the consumer/ carer without engendering concern, resistance or other adverse reactions
	Counsel patients when handing out medicines and explain the main adverse effects and special considerations, including storage and food requirements	Awareness of the importance of counselling patients when handing out medicines and explain the main adverse effects and special considerations, including storage and food requirements	Understand the importance of counselling patients when handing out medicines and explain the main adverse effects and special considerations, including storage and food requirements	Counsel patients when handing out medicines and explain the main adverse effects and special considerations, including storage and food requirements	Define processes to counsel patients when handing out medicines and explain the main adverse effects and special considerations, including storage and food requirements	Review and implement processes to counsel patients when handing out medicines and explain the main adverse effects and special considerations, including storage and food requirements
	Obtain sufficient information about a patient request to determine if the situation can be managed by the individual or referred	Awareness of the importance of obtaining sufficient information about a patient request to determine if the situation can be managed by the individual or referred	Understand the importance of obtaining sufficient information about a patient request to determine if the situation can be managed by the individual or referred	Obtain sufficient information about a patient request to determine if the situation can be managed by the individual or referred	Analyse the process to obtain sufficient information about a patient request to determine if the situation can be managed by the individual or referred	Review and implement the process to obtain sufficient information about a patient request to determine if the situation can be managed by the individual or referred

COMPETENCY	BEHAVIOURAL COMPETENCIES	Associate	Practitioner	Specialist	Professional	Leader
4.2 Provide information and advice to the product user/patient (cont.)	Clarify the nature and duration of the symptoms/ condition, other associated symptoms or signs, current or recent medications, and actions/treatments already used and their effectiveness, asking appropriate questions when the required information is not readily volunteered	Awareness of the importance of clarifying the nature and duration of the symptoms/ condition, other associated symptoms or signs, current or recent medications, and actions/treatments already used and their effectiveness, asking appropriate questions when the required information is not readily volunteered	Understand the importance of clarifying the nature and duration of the symptoms/ condition, other associated symptoms or signs, current or recent medications, and actions/treatments already used and their effectiveness, asking appropriate questions when the required information is not readily volunteered	Clarify the nature and duration of the symptoms/ condition, other associated symptoms or signs, current or recent medications, and actions/treatments already used and their effectiveness, asking appropriate questions when the required information is not readily volunteered	Define standards to clarify the nature and duration of the symptoms/ condition, other associated symptoms or signs, current or recent medications, and actions/treatments already used and their effectiveness, asking appropriate questions when the required information is not readily volunteered	Review standards to clarify the nature and duration of the symptoms/ condition, other associated symptoms or signs, current or recent medications, and actions/treatments already used and their effectiveness, asking appropriate questions when the required information is not readily volunteered
	Describe and justify additional clinical information required (e.g., concurrent medical conditions, laboratory test results) to form an opinion about the treatment options	Awareness of the requirement of additional clinical information required (e.g. concurrent medical conditions, laboratory test results) to form an opinion about the treatment options	Understand the requirement of additional clinical information required (e.g. concurrent medical conditions, laboratory test results) to form an opinion about the treatment options	Describe and justify additional clinical information required (e.g. concurrent medical conditions, laboratory test results) to form an opinion about the treatment options	Define standards for describing and justifying additional clinical information required (e.g. concurrent medical conditions, laboratory test results) to form an opinion about the treatment options	Review and implement standards for describing and justifying additional clinical information required (e.g. concurrent medical conditions, laboratory test results) to form an opinion about the treatment options
	Supply non-prescription medicines, therapies and diagnostic aids to meet patients' needs	Awareness of the need to identify issues with medicines, dose forms and methods of administration that need to be discussed or referred	Follow processes to assist in the identification of issues with medicines, dose forms and methods of administration that need to be discussed or referred	Identify issues with medicines, dose forms and methods of administration that need to be discussed or referred	Define standards to identify issues with medicines, dose forms and methods of administration that need to be discussed or referred	Review standards to identify issues with medicines, dose forms and methods of administration that need to be discussed or referred
	Identify, prioritise and act upon medicine-medicine interactions, medicine-disease interactions, medicine-patient interactions and medicine-food interactions	Awareness of the need to identify, prioritise and act upon medicine-medicine interactions, medicine-disease interactions, medicine-patient interactions and medicine-food interactions	Understand the need to identify, prioritise and act upon medicine-medicine interactions, medicine-disease interactions, medicine-patient interactions and medicine-food interactions	Identify, prioritise and act upon medicine-medicine interactions, medicine-disease interactions, medicine-patient interactions and medicine-food interactions	Define standards to identify, prioritise and act upon medicine-medicine interactions, medicine-disease interactions, medicine-patient interactions and medicine-food interactions	Review and implement standards to identify, prioritise and act upon medicine-medicine interactions, medicine-disease interactions, medicine-patient interactions and medicine-food interactions
	Report defective or substandard medicines to the appropriate authorities	Assist in the reporting of defective or substandard medicines to the appropriate authorities	Assist in the reporting of defective or substandard medicines to the appropriate authorities	Report defective or substandard medicines to the appropriate authorities	Analyse the process of reporting of defective or substandard medicines to the appropriate authorities	Review and implement the process of reporting of defective or substandard medicines to the appropriate authorities
	Discuss ways in which consumer privacy and confidentiality may be protected during a clinical consultation	Discuss ways in which consumer privacy and confidentiality may be protected during a clinical consultation	Discuss ways in which consumer privacy and confidentiality may be protected during a clinical consultation	Discuss ways in which consumer privacy and confidentiality may be protected during a clinical consultation	Discuss ways in which consumer privacy and confidentiality may be protected during a clinical consultation	Define and review ways in which consumer privacy and confidentiality may be protected during a clinical consultation

COMPETENCY	BEHAVIOURAL COMPETENCES	Associate	Practitioner	Specialist	Professional	Leader
**4.2 Provide information and advice to the product user/patient (cont.)**	Describe circumstances for which the consumer's right to receive primary healthcare services anonymously should be protected	Describe circumstances for which the consumer's right to receive primary healthcare services anonymously should be protected	Describe circumstances for which the consumer's right to receive primary healthcare services anonymously should be protected	Describe circumstances for which the consumer's right to receive primary healthcare services anonymously should be protected	Analyse circumstances for which the consumer's right to receive primary healthcare services anonymously should be protected	Define circumstances for which the consumer's right to receive primary healthcare services anonymously should be protected
**4.3 Report product use/ consumption (cont.)**	Use patient records as a tool for calculating usage	Awareness of the use of patient records as a tool for calculating usage	Enable the use of patient records as a tool for calculating usage	Use patient records as a tool for calculating usage	Evaluate methods for using patient records as a tool for calculating usage	Review and implement methods for using patient records as a tool for calculating usage
	Review records to provide information for government and NGO reporting	Awareness of the need for correct records to provide information for government and NGO reporting	Understand the need for correct records to provide information for government and NGO reporting	Review records to provide information for government and NGO reporting	Analyse the process for collection of records to provide information for government and NGO reporting	Review the process for collection of records to provide information for government and NGO reporting
	Record the quantities of drugs dispensed on the daily activity register every time drugs are dispensed	Record the quantities of drugs dispensed on the daily activity register every time drugs are dispensed	Record the quantities of drugs dispensed on the daily activity register every time drugs are dispensed	Record the quantities of drugs dispensed on the daily activity register every time drugs are dispensed	Provide analysis of the process for the recording of the quantities of drugs dispensed on the daily activity register every time drugs are dispensed	Review the process for the recording of the quantities of drugs dispensed on the daily activity register every time drugs are dispensed
	Update the stock control card every time products are received, issued or transferred to another facility	Update the stock control card every time products are received, issued or transferred to another facility	Update the stock control card every time products are received, issued or transferred to another facility	Update the stock control card every time products are received, issued or transferred to another facility	Provide analysis of the process for the updating of the stock control card every time products are received, issued or transferred to another facility	Review the process for the updating of the stock control card every time products are received, issued or transferred to another facility
	Compile and send the report and requisition form to the upper levels of the supply chain in time for the reporting requisition period	Awareness of the need to compile and send the report and requisition form to the upper levels of the supply chain in time for the reporting requisition period	Provide input to compile and send the report and requisition form to the upper levels of the supply chain in time for the reporting requisition period	Compile and send the report and requisition form to the upper levels of the supply chain in time for the reporting requisition period	Analyse the received report and requisition form during the reporting requisition period	Review the received report and requisition form during the reporting requisition period
**4.4. Dispense or provide commodities to patients/ users (i.e., ensuring the product goes "the last mile" appropriately)**	List which medicines are allowed to be prescribed by different prescribers and how to monitor this	Understand that lists that list which medicines are allowed to be prescribed by different prescribers must be complied to	Comply with lists that list which medicines are allowed to be prescribed by different prescribers	List which medicines are allowed to be prescribed by different prescribers and how to monitor this	Analyse the process to list which medicines are allowed to be prescribed by different prescribers and how to monitor this	Review and define the process to list which medicines are allowed to be prescribed by different prescribers and how to monitor this
	Validate prescriptions, ensuring they are correctly interpreted and legal	Awareness that prescriptions, must be correctly interpreted and legal	Understand that prescriptions, must be correctly interpreted and legal	Validate prescriptions, ensuring they are correctly interpreted and legal	Analyse prescriptions, ensuring they are correctly interpreted and legal	Review results of analysis of prescriptions, ensuring they are correctly interpreted and legal
	Supply medication to patients, considering packaging, storage and labelling	Supply medication to patients, considering packaging, storage and labelling	Supply medication to patients, considering packaging, storage and labelling	Supply medication to patients, considering packaging, storage and labelling	Analyse the process of supplying medication to patients, considering packaging, storage and labelling	Review the process of supplying medication to patients, considering packaging, storage and labelling

COMPETENCY	BEHAVIOURAL COMPETENCES	Associate	Practitioner	Specialist	Professional	Leader
**4.4. Dispense or provide commodities to patients/ users (i.e., ensuring the product goes "the last mile" appropriately) (cont.)**	Identify which medicines are especially dangerous and need more care when dispensing	Identify which medicines are especially dangerous and need more care when dispensing	Identify which medicines are especially dangerous and need more care when dispensing	Identify which medicines are especially dangerous and need more care when dispensing	Define rules for identifying which medicines are especially dangerous and need more care when dispensing	Review rules for identifying which medicines are especially dangerous and need more care when dispensing
	Describe checking processes required to ensure consumer safety (e.g. double check of calculations, weight and measurements, quarantine of products prior to final check and release, label reconciliation, and check and release of final products)	Perform checking processes required to ensure consumer safety (e.g. double check of calculations, weight and measurements, quarantine of products prior to final check and release, label reconciliation, and check and release of final products)	Enable checking processes required to ensure consumer safety (e.g. double check of calculations, weight and measurements, quarantine of products prior to final check and release, label reconciliation, and check and release of final products)	Describe checking processes required to ensure consumer safety (e.g. double check of calculations, weight and measurements, quarantine of products prior to final check and release, label reconciliation, and check and release of final products)	Define checking processes required to ensure consumer safety (e.g. double check of calculations, weight and measurements, quarantine of products prior to final check and release, label reconciliation, and check and release of final products)	Review checking processes required to ensure consumer safety (e.g. double check of calculations, weight and measurements, quarantine of products prior to final check and release, label reconciliation, and check and release of final products)
	Act upon dispensing errors	Enable the process of acting upon dispensing errors	Enable the process of acting upon dispensing errors	Act upon dispensing errors	Analyse the process for acting upon dispensing errors	Review an implement processes for acting upon dispensing errors
	Keep clients up to date about decisions that affect them; adjust services based on feedback from upper levels as appropriate	Awareness of the process of keeping clients up to date about decisions that affect them; adjust services based on feedback from upper levels as appropriate	Enable the process of keeping clients up to date about decisions that affect them; adjust services based on feedback from upper levels as appropriate	Keep clients up to date about decisions that affect them; adjust services based on feedback from upper levels as appropriate	Define the process of keeping clients up to date about decisions that affect them; adjust services based on feedback from upper levels as appropriate	Review and implement the process of keeping clients up to date about decisions that affect them; adjust services based on feedback from upper levels as appropriate
	List the risks to consumer safety posed by care that extends between care settings (e.g. hospital to community, hospital to hospital) and/ or is delivered by multiple healthcare providers (GP to specialist, GP to pharmacist)	List the risks to consumer safety posed by care that extends between care settings (e.g. hospital to community, hospital to hospital) and/ or is delivered by multiple healthcare providers (GP to specialist, GP to pharmacist)	List the risks to consumer safety posed by care that extends between care settings (e.g. hospital to community, hospital to hospital) and/ or is delivered by multiple healthcare providers (GP to specialist, GP to pharmacist)	List the risks to consumer safety posed by care that extends between care settings (e.g. hospital to community, hospital to hospital) and/ or is delivered by multiple healthcare providers (GP to specialist, GP to pharmacist)	Provide research for the list of risks to consumer safety posed by care that extends between care settings (e.g. hospital to community, hospital to hospital) and/ or is delivered by multiple healthcare providers (GP to specialist, GP to pharmacist)	Define and advocate the list of risks to consumer safety posed by care that extends between care settings (e.g. hospital to community, hospital to hospital) and/ or is delivered by multiple healthcare providers (GP to specialist, GP to pharmacist)
	Provide assistance to meet the requirements, requests and concerns of clients; provide accurate information to answer client questions; look for ways to help clients by identifying and proposing appropriate solutions and/or services	Provide assistance to meet the requirements, requests and concerns of clients.	Provide assistance to meet the requirements, requests and concerns of clients.	Provide assistance to meet the requirements, requests and concerns of clients; provide accurate information to answer client questions; look for ways to help clients by identifying and proposing appropriate solutions and/or services	Provide assistance to meet the requirements, requests and concerns of clients; provide accurate information to answer client questions; look for ways to help clients by identifying and proposing appropriate solutions and/or services	Provide assistance to meet the requirements, requests and concerns of clients; provide accurate information to answer client questions; look for ways to help clients by identifying and proposing appropriate solutions and/or services
	Ensure patients are transferred from hospitals to clinics with a continuing supply of medicine	Awareness of the process of ensuring patients are transferred from hospitals to clinics with a continuing supply of medicine	Enable the process of ensuring patients are transferred from hospitals to clinics with a continuing supply of medicine	Ensure patients are transferred from hospitals to clinics with a continuing supply of medicine	Analyse the process of ensuring patients are transferred from hospitals to clinics with a continuing supply of medicine	Review and implement the process of ensuring patients are transferred from hospitals to clinics with a continuing supply of medicine

5. Resource Management

COMPETENCY	BEHAVIOURAL COMPETENCIES (including knowledge requirements)	Associate	Practitioner	Specialist	Professional	Leader
5.1 Design and implement supply chain system and strategies	Design a responsive, agile and efficient supply chain that has the ability to meet the changing needs of customers and deliver high-quality products with short lead times at low cost	Basic operational knowledge of a responsive, agile and efficient supply chain that has the ability to meet the changing needs of customers and deliver high-quality products with short lead times at low cost	Enable a responsive, agile and efficient supply chain that has the ability to meet the changing needs of customers and deliver high-quality products with short lead times at low cost	Apply a responsive, agile and efficient supply chain that has the ability to meet the changing needs of customers and deliver high-quality products with short lead times at low cost	Demonstrate a responsive, agile and efficient supply chain that has the ability to meet the changing needs of customers and deliver high-quality products with short lead times at low cost	Design a responsive, agile and efficient supply chain that has the ability to meet the changing needs of customers and deliver high-quality products with short lead times at low cost
	Describe the interrelationship of organisations, people, technology, activities, information, and resources involved in moving a product from supplier to customer	Awareness of the interrelationship of organisations, people, technology, activities, information, and resources involved in moving a product from supplier to customer	Examine the interrelationship of organisations, people, technology, activities, information, and resources involved in moving a product from supplier to customer	Describe the interrelationship of organisations, people, technology, activities, information, and resources involved in moving a product from supplier to customer	Demonstrate the interrelationship of organisations, people, technology, activities, information, and resources involved in moving a product from supplier to customer	Establish the interrelationship of organisations, people, technology, activities, information, and resources involved in moving a product from supplier to customer
	List the management activities carried out in the course of running an organisation, including controlling, leading, monitoring, adjusting, organising and planning	Awareness of the management activities carried out in the course of running an organisation, including controlling, leading, monitoring, adjusting, organising and planning	Understand the management activities carried out in the course of running an organisation, including controlling, leading, monitoring, adjusting, organising and planning	List the management activities carried out in the course of running an organisation, including controlling, leading, monitoring, adjusting, organising and planning	Demonstrate the management activities carried out in the course of running an organisation, including controlling, leading, monitoring, adjusting, organising and planning	Illustrate the management activities carried out in the course of running an organisation, including controlling, leading, monitoring, adjusting, organising and planning
	Describe the characteristics unique to public health; demonstrate awareness of the factors that could contribute to or hinder the delivery of logistics services	Basic operational knowledge of the characteristics unique to public health, demonstrate awareness of the factors that could contribute to or hinder the delivery of logistics services	Understand the characteristics unique to public health, demonstrate awareness of the factors that could contribute to or hinder the delivery of logistics services	Describe the characteristics unique to public health, demonstrate awareness of the factors that could contribute to or hinder the delivery of logistics services	Determine the characteristics unique to public health, demonstrate awareness of the factors that could contribute to or hinder the delivery of logistics services	Strategically analyse the characteristics unique to public health, demonstrate awareness of the factors that could contribute to or hinder the delivery of logistics services
	Demonstrate awareness of emerging concepts and principles in public health logistics; adapt current practices to incorporate new developments in the field	Awareness of emerging concepts and principles in public health logistics; adapt current practices to incorporate new developments in the field	Examine emerging concepts and principles in public health logistics; adapt current practices to incorporate new developments in the field	Describe emerging concepts and principles in public health logistics; adapt current practices to incorporate new developments in the field	Demonstrate awareness of emerging concepts and principles in public health logistics; adapt current practices to incorporate new developments in the field	Strategically analyse emerging concepts and principles in public health logistics; adapt current practices to incorporate new developments in the field
	Determine which type of inventory control system will be most effective for a programme or country	Awareness of which type of inventory control system will be most effective for a particular programme or country	Compare which type of inventory control system will be most effective for a particular programme or country	Describe which type of inventory control system will be most effective for a particular programme or country	Determine which type of inventory control system will be most effective for a programme or country	Strategically analyse which type of inventory control system will be most effective for a programme or country

COMPETENCY	BEHAVIOURAL COMPETENCIES (including knowledge requirements)	Associate	Practitioner	Specialist	Professional	Leader
5.1 Design and implement supply chain system and strategies (cont.)	Explain the difference between continuous and period review systems of inventory control	Awareness of the difference between continuous and period review systems of inventory control	Compare the difference between continuous and period review systems of inventory control	Explain the difference between continuous and period review systems of inventory control	Demonstrate the difference between continuous and period review systems of inventory control	Demonstrate in-depth knowledge the difference between continuous and period review systems of inventory control
	Develop and implement a formal logistics strategy and plan	Observe and execute a formal logistics strategy and plan	Enable and follow a formal logistics strategy and plan	Define and apply a formal logistics strategy and plan	Determine and demonstrate a formal logistics strategy and plan	Develop and implement a formal logistics strategy and plan

COMPETENCY	BEHAVIOURAL COMPETENCIES (including knowledge requirements)	Associate	Practitioner	Specialist	Professional	Leader
5.2 Oversee operating of a logistics management information system	Integrate activities across organisations in the supply chain by ensuring information visibility in inventory levels, production and materials in transit	Observe information visibility in inventory levels, production and materials in transit	Enable information visibility in inventory levels, production and materials in transit	Apply information visibility in inventory levels, production and materials in transit	Analyse activities across organisations in the supply chain by ensuring information visibility in inventory levels, production and materials in transit	Integrate activities across organisations in the supply chain by ensuring information visibility in inventory levels, production and materials in transit
	Use appropriate recording systems. (e.g. stock cards, order forms, computer systems)	Awareness of appropriate recording systems. (e.g. stock cards, order forms, computer systems)	Understand appropriate recording systems. (e.g. stock cards, order forms, computer systems)	Use appropriate recording systems. (e.g. stock cards, order forms, computer systems)	Demonstrate the use of appropriate recording systems. (e.g. stock cards, order forms, computer systems)	Implement the use of appropriate recording systems. (e.g. stock cards, order forms, computer systems)
	Aggregate, analyse, and interpret logistics data to produce reports on logistics system performance	Awareness of the process of aggregating, analysing, and interpreting logistics data to produce reports on logistics system performance	Understand the process of aggregating, analysing, and interpreting logistics data to produce reports on logistics system performance	Aggregate, analyse, and interpret logistics data to produce reports on logistics system performance	Evaluate the process of aggregating, analysing, and interpreting logistics data to produce reports on logistics system performance	Implement the process of aggregating, analysing, and interpreting logistics data to produce reports on logistics system performance
	Disseminate reports up and down the supply chain to all appropriate stakeholders	Awareness that reports are disseminated up and down the supply chain to all appropriate stakeholders	Understand that reports are disseminated up and down the supply chain to all appropriate stakeholders	Disseminate reports up and down the supply chain to all appropriate stakeholders	Evaluate reports that are disseminated up and down the supply chain to all appropriate stakeholders	Develop reports that are disseminated up and down the supply chain to all appropriate stakeholders
	Communicate directly with facilities for receipt, review and approval of reports and/or orders; follow up on missing reports/ orders; and generate feedback reports for the facilities	Awareness of the need to communicate directly with facilities for receipt, review and approval of reports and/or orders; follow up on missing reports/ orders; and generate feedback reports for the facilities	Understand the need to communicate directly with facilities for receipt, review and approval of reports and/or orders; follow up on missing reports/ orders; and generate feedback reports for the facilities	Communicate directly with facilities for receipt, review and approval of reports and/or orders; follow up on missing reports/ orders; and generate feedback reports for the facilities	Ensure the process of communication directly with facilities for receipt, review and approval of reports and/or orders; follow up on missing reports/ orders; and generate feedback reports for the facilities	Develop the methodology for communication directly with facilities for receipt, review and approval of reports and/or orders; follow up on missing reports/ orders; and generate feedback reports for the facilities
	Monitor the supply pipeline and assess stock status	Awareness of the need to monitor the supply pipeline and assessing stock status	Understand the need to monitor the supply pipeline and assessing stock status	Monitor the supply pipeline and assess stock status	Analyse the process of monitoring the supply pipeline and assessing stock status	Implement the process of monitoring the supply pipeline and assessing stock status
	Apply a logical system for secure storage of records	Execute a logical system for secure storage of records	Enable a logical system for secure storage of records	Apply a logical system for secure storage of records	Demonstrate a logical system for secure storage of records	Develop and implement a logical system for secure storage of records
	Establish the urgency of required information	Awareness of the urgency of required information	Understand the urgency of required information	Recognize the urgency of required information	Demonstrate the urgency of required information	Establish the urgency of required information

COMPETENCY	BEHAVIOURAL COMPETENCIES (including knowledge requirements)	Associate	Practitioner	Specialist	Professional	Leader
	Assess specific requirements for undertaking work (e.g. information, stock, equipment, access to specific expertise) and ensure those requirements are or can be met	Execute the process of assessing specific requirements for undertaking work (e.g. information, stock, equipment, access to specific expertise) and ensuring those requirements are or can be met	Follow the process of assessing specific requirements for undertaking work (e.g. information, stock, equipment, access to specific expertise) and ensuring those requirements are or can be met	Apply the process of assessing specific requirements for undertaking work (e.g. information, stock, equipment, access to specific expertise) and ensuring those requirements are or can be met	Assess specific requirements for undertaking work (e.g. information, stock, equipment, access to specific expertise) and ensure those requirements are or can be met	Implement the process of assessing specific requirements for undertaking work (e.g. information, stock, equipment, access to specific expertise) and ensuring those requirements are or can be met
	Demonstrate a detailed knowledge of the national medicines supply information system (electronic or manual)	Basic operational knowledge of the national medicines supply information system (electronic or manual)	Show knowledge of the national medicines supply information system (electronic or manual)	Apply knowledge of the national medicines supply information system (electronic or manual)	Demonstrate a detailed knowledge of the national medicines supply information system (electronic or manual)	Demonstrate in-depth knowledge of the national medicines supply information system (electronic or manual)
**5.3 Implement risk management and monitoring and evaluation activities for the supply chain**						
**5.3.1 Ensure monitoring and evaluation activities are completed**	Perform periodic evaluations to maintain processes by gathering pertinent information — such as problem symptoms from knowledgeable sources, carrying these through to the problems, potential causes and root causes of the problem	Awareness of the periodic evaluations to maintain processes by gathering pertinent information — such as problem symptoms from knowledgeable sources, carrying these through to the problems, potential causes and root causes of the problem	Understand the periodic evaluations to maintain processes by gathering pertinent information — such as problem symptoms from knowledgeable sources, carrying these through to the problems, potential causes and root causes of the problem	Describe the periodic evaluations to maintain processes by gathering pertinent information — such as problem symptoms from knowledgeable sources, carrying these through to the problems, potential causes and root causes of the problem	Perform periodic evaluations of pertinent information — such as problem symptoms from knowledgeable sources, carrying these through to the problems, potential causes and root causes of the problem	Strategically analyse pertinent information — such as problem symptoms from knowledgeable sources, carrying these through to the problems, potential causes and root causes of the problem to maintain processes
	Quantify the operational performance of similar companies and establish internal targets based on best-in-class results	Awareness of the process of quantifying the operational performance of similar companies and establish internal targets based on best-in-class results	Understand the process of quantifying the operational performance of similar companies and establish internal targets based on best-in-class results	Quantify the operational performance of similar companies and establish internal targets based on best-in-class results	Determine the process of quantifying the operational performance of similar companies and establish internal targets based on best-in-class results	Implement the process of quantifying the operational performance of similar companies and establish internal targets based on best-in-class results
	Establish key performance measurements and continuous process improvement initiatives to improve process quality on a continual basis	Awareness of key performance measurements and continuous process improvement initiatives to improve process quality on a continual basis	Enable key performance measurements and continuous process improvement initiatives to improve process quality on a continual basis	Apply key performance measurements and continuous process improvement initiatives to improve process quality on a continual basis	Analyse key performance measurements and continuous process improvement initiatives to improve process quality on a continual basis	Establish key performance measurements and continuous process improvement initiatives to improve process quality on a continual basis

COMPETENCY	BEHAVIOURAL COMPETENCIES (including knowledge requirements)	Associate	Practitioner	Specialist	Professional	Leader
**5.3.1 Ensure monitoring and evaluation activities are completed (cont.)**	Provide results of the analysis to colleagues	Awareness of the need to provide results of the analysis to colleagues	Understand the need to provide results of the analysis to colleagues	Provide results of the analysis to colleagues	Analyse the results of the analysis provided	Strategically analyse the results of the analysis provided
	Control and check errors, taking corrective action so deviation from standards are minimised and the organisation's goals are achieved	Awareness of the need to control and check errors, taking corrective action so deviation from standards are minimised and the organisation's goals are achieved	Understand of the need to control and check errors, taking corrective action so deviation from standards are minimised and the organisation's goals are achieved	Control and check errors, taking corrective action so deviation from standards are minimised and the organisation's goals are achieved	Determine the process of controlling and checking errors, taking corrective action so deviation from standards are minimised and the organisation's goals are achieved	Implement the process of controlling and checking errors, taking corrective action so deviation from standards are minimised and the organisation's goals are achieved
	Consider the use of appropriate technological developments to improve the system	Awareness of the use of appropriate technological developments to improve the system	Examine the use of appropriate technological developments to improve the system	Identify the use of appropriate technological developments to improve the system	Analyse the use of appropriate technological developments to improve the system	Consider the use of appropriate technological developments to improve the system
	Recognise that continuous process improvement is an accepted way of organisational life	Realize that continuous process improvement is an accepted way of organisational life	Understand that continuous process improvement is an accepted way of organisational life	Describe that continuous process improvement is an accepted way of organisational life	Demonstrate that continuous process improvement is an accepted way of organisational life	Recognize that continuous process improvement is an accepted way of organisational life
	Execute ways of eliminating unnecessary steps in system design	Awareness that ways of eliminating unnecessary steps in system design are often possible	Examine ways of eliminating unnecessary steps in system design	Execute ways of eliminating unnecessary steps in system design	Demonstrate ways of eliminating unnecessary steps in system design	Implement ways of eliminating unnecessary steps in system design
	Develop processes that strive to eliminate waste	Execute processes that strive to eliminate waste	Enable processes that strive to eliminate waste	Apply processes that strive to eliminate waste	Analyse processes that strive to eliminate waste	Develop processes that strive to eliminate waste
	Review processes to encourage sustainability (e.g. reducing carbon footprint, establishing a paperless office, using renewable energy)	Execute processes to encourage sustainability (e.g. reducing carbon footprint, establishing a paperless office, using renewable energy)	Enable processes to encourage sustainability (e.g. reducing carbon footprint, establishing a paperless office, using renewable energy)	Apply processes to encourage sustainability (e.g. reducing carbon footprint, establishing a paperless office, using renewable energy)	Review processes to encourage sustainability (e.g. reducing carbon footprint, establishing a paperless office, using renewable energy)	Implement processes to encourage sustainability (e.g. reducing carbon footprint, establishing a paperless office, using renewable energy)
	Describe the systematic approach used to close process or system performance gaps through streamlining and cycle time reduction, and identify and eliminate causes of quality below specifications, process variation and non-value-adding activities	Awareness of the systematic approach used to close process or system performance gaps through streamlining and cycle time reduction, and identify and eliminate causes of quality below specifications, process variation and non-value-adding activities	Understand the systematic approach used to close process or system performance gaps through streamlining and cycle time reduction, and identify and eliminate causes of quality below specifications, process variation and non-value-adding activities	Describe the systematic approach used to close process or system performance gaps through streamlining and cycle time reduction, and identify and eliminate causes of quality below specifications, process variation and non-value-adding activities	Demonstrate the systematic approach used to close process or system performance gaps through streamlining and cycle time reduction, and identify and eliminate causes of quality below specifications, process variation and non-value-adding activities	Direct the systematic approach used to close process or system performance gaps through streamlining and cycle time reduction, and identify and eliminate causes of quality below specifications, process variation and non-value-adding activities
	Outline the relationship between technology and process functionality	Awareness of the relationship between technology and process functionality	Examine the relationship between technology and process functionality	Outline the relationship between technology and process functionality	Determine the relationship between technology and process functionality	Strategically analyse the relationship between technology and process functionality

COMPETENCY	BEHAVIOURAL COMPETENCIES (including knowledge requirements)	Associate	Practitioner	Specialist	Professional	Leader
5.3.2 Implement risk management activities	Identify risks affecting supply, transformation, delivery and customer demand	Observe risks affecting supply, transformation, delivery and customer demand	Examine risks affecting supply, transformation, delivery and customer demand	Identify risks affecting supply, transformation, delivery and customer demand	Determine risks affecting supply, transformation, delivery and customer demand	Strategically analyse risks affecting supply, transformation, delivery and customer demand
	Identify unsafe working conditions and take prompt, corrective action; stay alert to and take preventive action against hazards and threats; recommend measures to protect employees from hazardous working conditions; follow protocol for reporting safety violations; and investigate accidents to determine how to prevent them in the future	Awareness of what constitutes an unsafe working conditions and how to take prompt, corrective action; stay alert to and take preventive action against hazards and threats; awareness of the process of recommending measures to protect employees from hazardous working conditions; follow protocol for reporting safety violations	Understand what constitutes an unsafe working conditions and how to take prompt, corrective action; stay alert to and take preventive action against hazards and threats; follow the process of recommending measures to protect employees from hazardous working conditions; follow protocol for reporting safety violations	Identify unsafe working conditions and take prompt, corrective action; stay alert to and take preventive action against hazards and threats; recommend measures to protect employees from hazardous working conditions; follow protocol for reporting safety violations; and investigate accidents to determine how to prevent them in the future	Determine what constitutes an unsafe working conditions and how to take prompt, corrective action; stay alert to and take preventive action against hazards and threats; recommend measures to protect employees from hazardous working conditions; follow protocol for reporting safety violations; and investigate accidents to determine how to prevent them in the future	Strategically analyse what constitutes an unsafe working conditions and how to take prompt, corrective action; stay alert to and take preventive action against hazards and threats; recommend measures to protect employees from hazardous working conditions; follow protocol for reporting safety violations; and investigate accidents to determine how to prevent them in the future
	Develop and/or implement training programmes/drills in which personnel participate (e.g. fire and armed hold-up drills, safe manual handling practices, use of mandatory safety clothing)	Execute training programmes/drills in which personnel participate (e.g. fire and armed hold-up drills, safe manual handling practices, use of mandatory safety clothing)	Follow training programmes/drills in which personnel participate (e.g. fire and armed hold-up drills, safe manual handling practices, use of mandatory safety clothing)	Apply training programmes/drills in which personnel participate (e.g. fire and armed hold-up drills, safe manual handling practices, use of mandatory safety clothing)	Demonstrate training programmes/drills in which personnel participate (e.g. fire and armed hold-up drills, safe manual handling practices, use of mandatory safety clothing)	Develop and/or implement training programmes/drills in which personnel participate (e.g. fire and armed hold-up drills, safe manual handling practices, use of mandatory safety clothing)
	Develop and apply systems of review (e.g. audit) to check compliance with policies and procedures intended to maintain workplace safety (e.g. manual handling techniques, correct use of protective clothing)	Execute systems of review (e.g. audit) to check compliance with policies and procedures intended to maintain workplace safety (e.g. manual handling techniques, correct use of protective clothing)	Enable systems of review (e.g. audit) to check compliance with policies and procedures intended to maintain workplace safety (e.g. manual handling techniques, correct use of protective clothing)	Apply systems of review (e.g. audit) to check compliance with policies and procedures intended to maintain workplace safety (e.g. manual handling techniques, correct use of protective clothing)	Analyse systems of review (e.g. audit) to check compliance with policies and procedures intended to maintain workplace safety (e.g. manual handling techniques, correct use of protective clothing)	Develop systems of review (e.g. audit) to check compliance with policies and procedures intended to maintain workplace safety (e.g. manual handling techniques, correct use of protective clothing)
	Ensure equipment and tools are operating to prescribed standards	Execute the process of ensuring equipment and tools are operating to prescribed standards	Ensure equipment and tools are operating to prescribed standards	Apply the process of ensuring equipment and tools are operating to prescribed standards	Determine the process of ensuring equipment and tools are operating to prescribed standards	Implement the process of ensuring equipment and tools are operating to prescribed standards
	Implement key security systems for the workplace (e.g. for cash, narcotics and other controlled substances, investigational drugs, consumer records, entry and exit points) and levels of access and/or authority applicable to each	Awareness of key security systems for the workplace (e.g. for cash, narcotics and other controlled substances, investigational drugs, consumer records, entry and exit points) and levels of access and/or authority applicable to each	Enable key security systems for the workplace (e.g. for cash, narcotics and other controlled substances, investigational drugs, consumer records, entry and exit points) and levels of access and/or authority applicable to each	Apply key security systems for the workplace (e.g. for cash, narcotics and other controlled substances, investigational drugs, consumer records, entry and exit points) and levels of access and/or authority applicable to each	Determine key security systems for the workplace (e.g. for cash, narcotics and other controlled substances, investigational drugs, consumer records, entry and exit points) and levels of access and/or authority applicable to each	Implement key security systems for the workplace (e.g. for cash, narcotics and other controlled substances, investigational drugs, consumer records, entry and exit points) and levels of access and/or authority applicable to each

COMPETENCY	BEHAVIOURAL COMPETENCIES (including knowledge requirements)	Associate	Practitioner	Specialist	Professional	Leader
5.3.2 Implement risk management activities (cont.)	Describe the prevention strategies adopted (e.g. protocols, security barriers, fixed or personal duress alarms)	Execute the prevention strategies adopted (e.g. protocols, security barriers, fixed or personal duress alarms)	Follow the prevention strategies adopted (e.g. protocols, security barriers, fixed or personal duress alarms)	Describe the prevention strategies adopted (e.g. protocols, security barriers, fixed or personal duress alarms)	Demonstrate the prevention strategies adopted (e.g. protocols, security barriers, fixed or personal duress alarms)	Implement the prevention strategies adopted (e.g. protocols, security barriers, fixed or personal duress alarms)
	Document critical incidents	Awareness of the need to document critical incidents	Understand the need to document critical incidents	Document critical incidents	Evaluate documented critical incidents	Strategically analyse documented critical incidents
	Engage in contingency planning and managing operational continuity	Awareness of contingency planning and the need to manage operational continuity	Understand contingency planning and the need to manage operational continuity	Provide input when engaging in contingency planning and managing operational continuity	Engage in contingency planning and managing operational continuity	Develop methodology for contingency planning and managing operational continuity
	Increase understanding of a safe working environment and engender a shared commitment from all personnel to creating one	Awareness of a safe working environment and engender a shared commitment from all personnel to creating one	Understand what constitutes a safe working environment and engender a shared commitment from all personnel to creating one	Increase understanding of a safe working environment and engender a shared commitment from all personnel to creating one	Ensure that there is an understanding of what a safe working environment is and engender a shared commitment from all personnel to creating one	Demonstrate an understanding of a safe working environment and engender a shared commitment from all personnel to creating one
	Demonstrate knowledge of relevant safety and health laws and regulations; comply with safety codes, standards and guidelines; adhere to worksite safety programmes; understand emergency response plans when they exist	Awareness of relevant safety and health laws and regulations; comply with safety codes, standards and guidelines; adhere to worksite safety programmes; understand emergency response plans when they exist	Understand relevant safety and health laws and regulations; comply with safety codes, standards and guidelines; adhere to worksite safety programmes; understand emergency response plans when they exist	Describe relevant safety and health laws and regulations; comply with safety codes, standards and guidelines; adhere to worksite safety programmes; understand emergency response plans when they exist	Demonstrate knowledge of relevant safety and health laws and regulations; comply with safety codes, standards and guidelines; adhere to worksite safety programmes; understand emergency response plans when they exist	Implement processes to comply with relevant safety and health laws and regulations; comply with safety codes, standards and guidelines; adhere to worksite safety programmes; understand emergency response plans when they exist
	Demonstrate knowledge of how to handle hazardous commodities and first aid procedures	Awareness of how to handle hazardous commodities and first aid procedures	Understand how to handle hazardous commodities and first aid procedures	Describe how to handle hazardous commodities and first aid procedures	Demonstrate knowledge of how to handle hazardous commodities and first aid procedures	Implement processes to handle hazardous commodities and first aid procedures
5.4 Manage outsourcing of SCM functions	Negotiate and compile contracts with outside parties for delivery of logistics services	Awareness of the process of negotiating and compiling contracts with outside parties for delivery of logistics services	Understand the process of negotiating and compiling contracts with outside parties for delivery of logistics services	Provide input on negotiating and compiling contracts with outside parties for delivery of logistics services	Negotiate and compile contracts with outside parties for delivery of logistics services	Formulate contracts with outside parties for delivery of logistics services

COMPETENCY	BEHAVIOURAL COMPETENCIES (including knowledge requirements)	Associate	Practitioner	Specialist	Professional	Leader
**5.5 Manage and plan projects**						
**5.5.1 Develop and direct project plans**	Anticipate obstacles and critical events for ensuring the six rights of logistics management (right quantity, product, condition, place, time and cost) and develop contingency plans to address them; monitor progress and take necessary corrective action when needed	Awareness of the process to anticipate obstacles and critical events for ensuring the six rights of logistics management (right quantity, product, condition, place, time and cost) and the contingency plans to address them; as well as the need to monitor progress and take necessary corrective action when needed	Understand the process to anticipate obstacles and critical events for ensuring the six rights of logistics management (right quantity, product, condition, place, time and cost) and the contingency plans to address them; as well as the need to monitor progress and take necessary corrective action when needed	Anticipate obstacles and critical events for ensuring the six rights of logistics management (right quantity, product, condition, place, time and cost) and develop contingency plans to address them; monitor progress and take necessary corrective action when needed	Evaluate obstacles and critical events for ensuring the six rights of logistics management (right quantity, product, condition, place, time and cost) and develop contingency plans to address them; monitor progress and take necessary corrective action when needed	Strategically analyse obstacles and critical events for ensuring the six rights of logistics management (right quantity, product, condition, place, time and cost) and develop contingency plans to address them; monitor progress and take necessary corrective action when needed
	Create an operational plan that is consistent with the strategic plan, links specific goals and strategies, and identifies the performance indicators to be used to measure achievements	Awareness of an operational plan that is consistent with the strategic plan, links specific goals and strategies, and identifies the performance indicators to be used to measure achievements	Understand an operational plan that is consistent with the strategic plan, links specific goals and strategies, and identifies the performance indicators to be used to measure achievements	Describe an operational plan that is consistent with the strategic plan, links specific goals and strategies, and identifies the performance indicators to be used to measure achievements	Determine an operational plan that is consistent with the strategic plan, links specific goals and strategies, and identifies the performance indicators to be used to measure achievements	Create an operational plan that is consistent with the strategic plan, links specific goals and strategies, and identifies the performance indicators to be used to measure achievements
	Demonstrate the use of information gathering techniques from stakeholders, analysing situations and identifying implications to make correct decisions	Awareness of information gathering techniques from stakeholders, analysing situations and identifying implications to make correct decisions	Enable the use of information gathering techniques from stakeholders, analysing situations and identifying implications to make correct decisions	Apply the use of information gathering techniques from stakeholders, analysing situations and identifying implications to make correct decisions	Demonstrate the use of information gathering techniques from stakeholders, analysing situations and identifying implications to make correct decisions	Implement the use of information gathering techniques from stakeholders, analysing situations and identifying implications to make correct decisions
	Answer the questions, "Where are we going?" and "How are we going to get there?" and create a specific and purposeful path to achieve this.	Awareness of the questions, "Where are we going?" and "How are we going to get there?" and the need to create a specific and purposeful path to achieve this.	Examine of the questions, "Where are we going?" and "How are we going to get there?" and the need to create a specific and purposeful path to achieve this.	Answer the questions, "Where are we going?" and "How are we going to get there?" and create a specific and purposeful path to achieve this.	Review the questions, "Where are we going?" and "How are we going to get there?" and create a specific and purposeful path to achieve this.	Analyse the questions, "Where are we going?" and "How are we going to get there?" and create a specific and purposeful path to achieve this.
	Apply the process reference model to managing supply chain processes (i.e. integrating business process re-engineering, benchmarking and process measurement)	Execute the process reference model to managing supply chain processes (i.e. integrating business process re-engineering, benchmarking and process measurement)	Enable the process reference model to managing supply chain processes (i.e. integrating business process re-engineering, benchmarking and process measurement)	Apply the process reference model to managing supply chain processes (i.e. integrating business process re-engineering, benchmarking and process measurement)	Demonstrate the process reference model to managing supply chain processes (i.e. integrating business process re-engineering, benchmarking and process measurement)	Implement the process reference model to managing supply chain processes (i.e. integrating business process re-engineering, benchmarking and process measurement)
	Capture the current state of a process and derive the desired future state	Awareness of the need to capture the current state of a process and derive the desired future state	Understand the process of capturing the current state of a process and deriving the desired future state	Capture the current state of a process and derive the desired future state	Analyse the current state of a process and derive the desired future state	Strategically analyse the current state of a process and derive the desired future state

COMPETENCY	BEHAVIOURAL COMPETENCIES (including knowledge requirements)	Associate	Practitioner	Specialist	Professional	Leader
**5.5.1 Develop and direct project plans (cont.)**	Select and apply tools or technological solutions to frequently encountered problems	Execute the process of selecting and applying tools or technological solutions to frequently encountered problems	Enable the process of selecting and applying tools or technological solutions to frequently encountered problems	Select and apply tools or technological solutions to frequently encountered problems	Demonstrate the process of selecting and applying tools or technological solutions to frequently encountered problems	Direct and implement the process of selecting and applying tools or technological solutions to frequently encountered problems
	Provide staff with the necessary tools to carry out plans	Awareness of the need to provide staff with the necessary tools to carry out plans	Understand the need to provide staff with the necessary tools to carry out plans	Provide staff with the necessary tools to carry out plans	Analyse the process of providing staff with the necessary tools to carry out plans	Direct the process of providing staff with the necessary tools to carry out plans
	Demonstrate the ability to monitor progress and make changes as required	Awareness of the need to monitor progress and make changes as required	Understand the need to monitor progress and make changes as required	Describe the process of monitoring progress and making changes as required	Demonstrate the ability to monitor progress and make changes as required	Implement the process of monitoring progress and making changes as required
	Facilitate meetings for planning, organisation and monitoring logistics activities	Awareness of meetings for planning, organisation and monitoring logistics activities	Provide input at meetings for planning, organisation and monitoring logistics activities	Facilitate meetings for planning, organisation and monitoring logistics activities	Convene meetings for planning, organisation and monitoring logistics activities	Direct meetings for planning, organisation and monitoring logistics activities
	Ensure staff are aware that they are accountable for achieving the desired results	Awareness that the staff are accountable for achieving the desired results	Understand that the staff are accountable for achieving the desired results	Describe the process of ensuring staff are aware that they are accountable for achieving the desired results	Ensure staff are aware that they are accountable for achieving the desired results	Implement the process of ensuring staff are aware that they are accountable for achieving the desired results
	Understand planning, organising and managing resources to bring about the successful completion of projects	Awareness that planning, organising and managing resources bring about the successful completion of projects	Understand that planning, organising and managing resources bring about the successful completion of projects	Describe how planning, organising and managing resources will bring about the successful completion of projects	Determine how planning, organising and managing resources will bring about the successful completion of projects	Illustrate planning, organising and managing resources to bring about the successful completion of projects
	Describe the key features of a successful change management strategy	Awareness of the key features of a successful change management strategy	Compare the key features of a successful change management strategy	Describe the key features of a successful change management strategy	Determine the key features of a successful change management strategy	Demonstrate in-depth knowledge of the key features of a successful change management strategy
**5.5.2 Execute strategic decision making**	Make decisions regarding one's own workload and area of responsibility	Realize that one can make decisions regarding one's own workload and area of responsibility	Understand that one can make decisions regarding one's own workload and area of responsibility	Make decisions regarding one's own workload and area of responsibility	Determine how one can make decisions regarding one's own workload and area of responsibility	Demonstrate making decisions regarding one's own workload and area of responsibility
	Prioritize competing tasks and perform them quickly and efficiently according to their importance; find new ways of organising or planning work to accomplish it more efficiently	Awareness of need to prioritise competing tasks and perform them quickly and efficiently according to their importance; finding new ways of organising or planning work to accomplish it more efficiently	Understand of need to prioritise competing tasks and perform them quickly and efficiently according to their importance; finding new ways of organising or planning work to accomplish it more efficiently	Prioritize competing tasks and perform them quickly and efficiently according to their importance; find new ways of organising or planning work to accomplish it more efficiently	Determine the process of prioritising competing tasks and perform them quickly and efficiently according to their importance; finding new ways of organising or planning work to accomplish it more efficiently	Illustrate the process of prioritising competing tasks and perform them quickly and efficiently according to their importance; finding new ways of organising or planning work to accomplish it more efficiently

COMPETENCY	BEHAVIOURAL COMPETENCIES (including knowledge requirements)	Associate	Practitioner	Specialist	Professional	Leader
5.5.2 Execute strategic decision making (cont.)	Ensure proper time and space for consultation around decisions being made.	Awareness of the need to ensure proper time and space for consultation around decisions being made.	Understand the need to ensure proper time and space for consultation around decisions being made.	Ensure proper time and space for consultation around decisions being made.	Evaluate the proper time and space for consultation around decisions being made.	Implement the proper time and space for consultation around decisions being made.
	Consider the input of staff needed	Awareness of the process of considering the input of staff needed	Follow the process of considering the input of staff needed	Consider the input of staff needed	Demonstrate the process of considering the input of staff needed	Implement the process of considering the input of staff needed
	Check assumptions against facts	Awareness of the need to check assumptions against facts	Understand the need to check assumptions against facts	Check assumptions against facts	Analyse assumptions against facts	Develop the process of checking assumptions against facts
	Identify the key issues in a complex situation and come to the heart of the problem quickly	Observe the key issues in a complex situation and come to the heart of the problem quickly	Examine the key issues in a complex situation and come to the heart of the problem quickly	Identify the key issues in a complex situation and come to the heart of the problem quickly	Determine the key issues in a complex situation and come to the heart of the problem quickly	Strategically analyse the key issues in a complex situation and come to the heart of the problem quickly
	Gather relevant information before making decisions	Awareness of the need to gather relevant information before making decisions	Examine relevant information before making decisions	Gather relevant information before making decisions	Evaluate relevant information before making decisions	Strategically analyse relevant information before making decisions
	Make strategic decisions in the interest of the organisation's goals	Awareness of the fact that strategic decisions are made in the interest of the organisation's goals	Understand that strategic decisions are made in the interest of the organisation's goals	Describe the process of providing input for making strategic decisions in the interest of the organisation's goals	Provide input to strategic decisions in the interest of the organisation's goals	Make strategic decisions in the interest of the organisation's goals
	Make tough decisions when necessary	Awareness of the need to make tough decisions when necessary	Understand when to make tough decisions when necessary	Make tough decisions when necessary	Analyse tough decisions when necessary	Demonstrate making tough decisions when necessary
	Consider the impact of decisions on others' work or team goals	Awareness of the impact of decisions on others' work or team goals	Understand the impact of decisions on others' work or team goals	Consider the impact of decisions on others' work or team goals	Analyse the impact of decisions on others' work or team goals	Strategically analyse the impact of decisions on others' work or team goals
	Communicate decisions and ensure they are incorporated into policies and processes	Awareness of the need to communicate decisions and ensure they are incorporated into policies and processes	Understand the process to communicate decisions and ensure they are incorporated into policies and processes	Describe the process to communicate decisions and ensure they are incorporated into policies and processes	Communicate decisions and ensure they are incorporated into policies and processes	Develop the process of communicating decisions and ensure they are incorporated into policies and processes
	Undertake inclusive and consultative strategic planning to establish strategic objectives	Awareness of inclusive and consultative strategic planning to establish strategic objectives	Understand inclusive and consultative strategic planning to establish strategic objectives	Describe inclusive and consultative strategic planning to establish strategic objectives	Demonstrate inclusive and consultative strategic planning to establish strategic objectives	Undertake inclusive and consultative strategic planning to establish strategic objectives
	Identify and support emerging trends and practices	Awareness of emerging trends and practices	Examine and enable emerging trends and practices	Identify and support emerging trends and practices	Evaluate emerging trends and practices	Strategically analyse and implement emerging trends and practices
	Outline the macro and long-term consequences of decisions	Awareness of the macro and long-term consequences of decisions	Understand the macro and long-term consequences of decisions	Describe the macro and long-term consequences of decisions	Analyse the macro and long-term consequences of decisions	Outline the macro and long-term consequences of decisions
5.5.3 Manage partnerships	Apply the concepts of partnership working	Execute the concepts of partnership working	Enable the concepts of partnership working	Apply the concepts of partnership working	Demonstrate the concepts of partnership working	Implement the concepts of partnership working

COMPETENCY	BEHAVIOURAL COMPETENCIES (including knowledge requirements)	Associate	Practitioner	Specialist	Professional	Leader
5.5.3 Manage partnerships (cont.)	Support implementation of partnership programmes	Awareness of the process of supporting implementation of partnership programmes	Understand the process of supporting implementation of partnership programmes	Support implementation of partnership programmes	Determine the process of supporting implementation of partnership programmes	Develop the process of supporting implementation of partnership programmes
	Experiment with and trial new ideas with partners	Awareness of the process of experimenting with and trialling new ideas with partners	Understand the process of experimenting with and trialling new ideas with partners	Describe the process of experimenting with and trialling new ideas with partners	Analyse the process of experimenting with and trialling new ideas with partners	Experiment with and trial new ideas with partners
	Build partnerships to deliver programme and increase impact	Awareness of the need to build partnerships to deliver programme and increase impact	Understand the need to build partnerships to deliver programme and increase impact	Identify partnerships to deliver programme and increase impact	Analyse partnerships to deliver programme and increase impact	Build partnerships to deliver programme and increase impact
	Promote innovation and creativity in partnership work	Execute innovation and creativity in partnership work	Enable innovation and creativity in partnership work	Use innovation and creativity in partnership work	Analyse innovation and creativity in partnership work	Promote innovation and creativity in partnership work
	Communicate key information with partner members	Awareness of the need to communicate key information with partner members	Understand the need to communicate key information with partner members	Describe the process of communicating key information with partner members	Communicate key information with partner members	Develop the process of communicating key information with partner members
	Involve and value partners in all aspects of programming	Awareness of the need to involve and value partners in all aspects of programming	Understand the need to involve and value partners in all aspects of programming	Describe the process of involving and valuing partners in all aspects of programming	Involve and value partners in all aspects of programming	Develop the process of involving and valuing partners in all aspects of programming
	Identify problems with supply chain relationships	Awareness of problems with supply chain relationships	Examine problems with supply chain relationships	Identify problems with supply chain relationships	Analyse problems with supply chain relationships	Strategically analyse problems with supply chain relationships
	Communicate effectively with nurses, doctors and other members of the healthcare team	Awareness of the need to communicate effectively with nurses, doctors and other members of the healthcare team	Understand the need to communicate effectively with nurses, doctors and other members of the healthcare team	Communicate effectively with nurses, doctors and other members of the healthcare team	Demonstrate the process of communicating effectively with nurses, doctors and other members of the healthcare team	Illustrate the process of communicating effectively with nurses, doctors and other members of the healthcare team
	Respond to consumer complaints or comments about services and/or advice received	Awareness of the need to respond to consumer complaints or comments about services and/or advice received	Understand the need to respond to consumer complaints or comments about services and/or advice received	Respond to consumer complaints or comments about services and/or advice received	Analyse the process of responding to consumer complaints or comments about services and/or advice received	Develop the process of responding to consumer complaints or comments about services and/or advice received
	Foster collaboration and consultation across the sector and encourage the team to work with others	Awareness of the need for collaboration and consultation across the sector and encourage the team to work with others	Understand the need for collaboration and consultation across the sector and encourage the team to work with others	Foster collaboration and consultation across the sector and encourage the team to work with others	Analyse collaboration and consultation across the sector and encourage the team to work with others	Illustrate collaboration and consultation across the sector and encourage the team to work with others
	Encourage input from other key actors in the humanitarian sector and develop relationships with a cross-section of actors	Awareness of the need to encourage input from other key actors in the humanitarian sector and develop relationships with a cross-section of actors	Understand the need to encourage input from other key actors in the humanitarian sector and develop relationships with a cross-section of actors	Assist in sharing input from other key actors in the humanitarian sector and develop relationships with a cross-section of actors	Encourage input from other key actors in the humanitarian sector and develop relationships with a cross-section of actors	Develop the process of sharing input from other key actors in the humanitarian sector and develop relationships with a cross-section of actors

COMPETENCY	BEHAVIOURAL COMPETENCIES (including knowledge requirements)	Associate	Practitioner	Specialist	Professional	Leader
5.5.3 Manage partnerships (cont.)	Meet the reporting requirements of vertical programmes	Awareness of the need to meet the reporting requirements of vertical programmes	Understand the need to meet the reporting requirements of vertical programmes	Meet the reporting requirements of vertical programmes	Analyse the process of meeting the reporting requirements of vertical programmes	Direct the process of meeting the reporting requirements of vertical programmes
	Outline the structure of the health system at a national level and explain this to others	Awareness of the structure of the health system at a national level and explain this to others	Understand the structure of the health system at a national level and explain this to others	Outline the structure of the health system at a national level	Analyse the structure of the health system at a national level and explain this to others	Illustrate the structure of the health system at a national level and explain this to others
	Discuss the role of other members of the healthcare team (including with consumers) in a way that engenders understanding and confidence in the team and its members	Awareness of the role of other members of the healthcare team (including with consumers) in a way that engenders understanding and confidence in the team and its members	Compare the role of other members of the healthcare team (including with consumers) in a way that engenders understanding and confidence in the team and its members	Discuss the role of other members of the healthcare team (including with consumers) in a way that engenders understanding and confidence in the team and its members	Analyse the roles of other members of the healthcare team (including with consumers) in a way that engenders understanding and confidence in the team and its members	Illustrate of the roles of other members of the healthcare team (including with consumers) in a way that engenders understanding and confidence in the team and its members
	Describe the structure of the organisation, environment and/ or service in which they work	Awareness of the structure of the organisation, environment and/ or service in which they work	Understand the structure of the organisation, environment and/ or service in which they work	Describe the structure of the organisation, environment and/ or service in which they work	Analyse the structure of the organisation, environment and/ or service in which they work	Direct the structure of the organisation, environment and/ or service in which they work
	Describe how vertical programmes work within the health system	Awareness of how vertical programmes work within the health system	Understand how vertical programmes work within the health system	Describe how vertical programmes work within the health system	Demonstrate how vertical programmes work within the health system	Illustrate how vertical programmes work within the health system
	Describe the communication network established to achieve work outcomes	Awareness of the communication network established to achieve work outcomes	Understand the communication network established to achieve work outcomes	Describe the communication network established to achieve work outcomes	Evaluate the communication network established to achieve work outcomes	Establish the communication network to achieve work outcomes
	Describe the structure of the health system at the provincial/regional level and explain this to others	Awareness of the structure of the health system at the provincial/regional level and explain this to others	Understand the structure of the health system at the provincial/regional level and explain this to others	Describe the structure of the health system at the provincial/regional level and explain this to others	Demonstrate knowledge of the structure of the health system at the provincial/regional level and explain this to others	Demonstrate in-depth knowledge of the structure of the health system at the provincial/regional level and explain this to others
	List the roles and functions of government agencies in regulating and supporting supply chain organisations	Awareness of the roles and functions of government agencies in regulating and supporting supply chain organisations	Understand the roles and functions of government agencies in regulating and supporting supply chain organisations	List the roles and functions of government agencies in regulating and supporting supply chain organisations	Analyse the roles and functions of government agencies in regulating and supporting supply chain organisations	Demonstrate in-depth knowledge of the roles and functions of government agencies in regulating and supporting supply chain organisations
5.5.4 Direct/ participate in teamwork	Work with team members within their area of authority to establish achievable goals and strategies that are consistent with the objectives established for the organisation as a whole	Awareness of the need to work with team members within their area of authority to establish achievable goals and strategies that are consistent with the objectives established for the organisation as a whole	Understand the need to work with team members within their area of authority to establish achievable goals and strategies that are consistent with the objectives established for the organisation as a whole	Describe the process of working with team members within their area of authority to establish achievable goals and strategies that are consistent with the objectives established for the organisation as a whole	Work with team members within their area of authority to establish achievable goals and strategies that are consistent with the objectives established for the organisation as a whole	Develop a methodology to work with team members within their area of authority to establish achievable goals and strategies that are consistent with the objectives established for the organisation as a whole
5.5.4 Direct/ participate in teamwork (cont.)	Develop an organisational chart that shows the lines of reporting and responsibility among staff	Awareness of an organisational chart that shows the lines of reporting and responsibility among staff	Interpret an organisational chart that shows the lines of reporting and responsibility among staff	Describe an organisational chart that shows the lines of reporting and responsibility among staff	Evaluate an organisational chart that shows the lines of reporting and responsibility among staff	Develop an organisational chart that shows the lines of reporting and responsibility among staff
	Actively contribute a perspective and make a positive contribution to team-based problem solving and decision making	Awareness of the need to actively contribute a perspective and making a positive contribution to team-based problem solving and decision making	Understand the need to actively contribute a perspective and making a positive contribution to team-based problem solving and decision making	Actively contribute a perspective and make a positive contribution to team-based problem solving and decision making	Determine a culture of actively contributing a perspective and making a positive contribution to team-based problem solving and decision making	Implement a culture of actively contributing a perspective and making a positive contribution to team-based problem solving and decision making
	Provide feedback, encouragement and support to team members for progressing strategic goals	Awareness of the need to provide feedback, encouragement and support to team members for progressing strategic goals	Understand the need to provide feedback, encouragement and support to team members for progressing strategic goals	Provide feedback, encouragement and support to team members for progressing strategic goals	Determine a culture of providing feedback, encouragement and support to team members for progressing strategic goals	Create a culture of providing feedback, encouragement and support to team members for progressing strategic goals
	Monitor team performance in relation to the organisation's mission and goals	Awareness of the need to monitor team performance in relation to the organisation's mission and goals	Understand the need to monitor team performance in relation to the organisation's mission and goals	Define team performance in relation to the organisation's mission and goals	Monitor team performance in relation to the organisation's mission and goals	Strategically analyse team performance in relation to the organisation's mission and goals
	Encourage teams to think for themselves and resolve problems	Awareness of the need for teams to think for themselves and resolve problems	Understand the need for teams to think for themselves and resolve problems	Encourage teams to think for themselves and resolve problems	Enable the creation of a culture of teams thinking for themselves and resolving problems	Create a culture of teams thinking for themselves and resolving problems
	Encourage and harness diversity within the team to boost team effectiveness	Awareness of the need to encourage and harness diversity within the team to boost team effectiveness	Understand the need to encourage and harness diversity within the team to boost team effectiveness	Encourage and harness diversity within the team to boost team effectiveness	Demonstrate the process of encouraging and harnessing diversity within the team to boost team effectiveness	Develop the process of encouraging and harnessing diversity within the team to boost team effectiveness
	Encourage and support the team to work through its stages of development and perform well	Awareness of the need to encourage and support the team to work through its stages of development and perform well	Understand the need to encourage and support the team to work through its stages of development and perform well	Encourage and support the team to work through its stages of development and perform well	Demonstrate the process of encouraging and supporting the team to work through its stages of development and perform well	Develop the process of encouraging and supporting the team to work through its stages of development and perform well
	Describe roles and responsibilities in relation to employees' expertise and the expectations of collaborating team members	Awareness of roles and responsibilities in relation to employees' expertise and the expectations of collaborating team members	Understand roles and responsibilities in relation to employees' expertise and the expectations of collaborating team members	Describe roles and responsibilities in relation to employees' expertise and the expectations of collaborating team members	Determine roles and responsibilities in relation to employees' expertise and the expectations of collaborating team members	Develop roles and responsibilities in relation to employees' expertise and the expectations of collaborating team members
	Describe where their position fits in the structure and their responsibilities and accountabilities	Realize where their position fits in the structure and their responsibilities and accountabilities	Understand where their position fits in the structure and their responsibilities and accountabilities	Describe where their position fits in the structure and their responsibilities and accountabilities	Demonstrate where their position fits in the structure and their responsibilities and accountabilities	Illustrate where their position fits in the structure and their responsibilities and accountabilities
5.6 Manage financial activities	Manage all resources with care, applying budgetary principles	Awareness of the need to manage all resources with care, applying budgetary principles	Follow rules for managing all resources with care, applying budgetary principles	Manage all resources with care, applying budgetary principles	Determine rules for managing all resources with care, applying budgetary principles	Create rules for managing all resources with care, applying budgetary principles

COMPETENCY	BEHAVIOURAL COMPETENCIES (including knowledge requirements)	Associate	Practitioner	Specialist	Professional	Leader
5.6 Manage financial activities (cont.)	Take overall responsibility for meeting budgets and donor requirements	Awareness of the need to take overall responsibility for meeting budgets and donor requirements	Understand the need to take overall responsibility for meeting budgets and donor requirements	Participate in meeting budgets and donor requirements	Take ownership for meeting budgets and donor requirements	Take overall responsibility for meeting budgets and donor requirements
	Create and manage budgets (national, regional, etc.) as necessary for work (e.g. wages budget, touring budget, stationery budget, project budgets for using NGO funds)	Awareness of budgets (national, regional, etc.) as necessary for work (e.g., wages budget, touring budget, stationery budget, project budgets for using NGO funds)	Understand budgets (national, regional, etc.) as necessary for work (e.g., wages budget, touring budget, stationery budget, project budgets for using NGO funds)	Recognize budgets (national, regional, etc.) as necessary for work (e.g., wages budget, touring budget, stationery budget, project budgets for using NGO funds)	Analyse budgets (national, regional, etc.) as necessary for work (e.g., wages budget, touring budget, stationery budget, project budgets for using NGO funds)	Create and manage budgets (national, regional, etc.) as necessary for work (e.g., wages budget, touring budget, stationery budget, project budgets for using NGO funds)
	Produce timely and clear financial reports for funders and donors	Awareness of the need to produce timely and clear financial reports for funders and donors	Understand the process of producing timely and clear financial reports for funders and donors	Describe the process of producing timely and clear financial reports for funders and donors	Produce timely and clear financial reports for funders and donors	Implement the process of producing timely and clear financial reports for funders and donors
	Keep records following government budgeting, accounting and financial practices	Awareness of the process of keeping records following government budgeting, accounting and financial practices	Understand the process of keeping records following government budgeting, accounting and financial practices	Describe the process of keeping records following government budgeting, accounting and financial practices	Keep records following government budgeting, accounting and financial practices	Implement the process of keeping records following government budgeting, accounting and financial practices
	Maintain an overview of multiple budgets from multiple sources	Awareness of the need to maintain an overview of multiple budgets from multiple sources	Understand the process of maintaining an overview of multiple budgets from multiple sources	Describe the process of maintaining an overview of multiple budgets from multiple sources	Maintain an overview of multiple budgets from multiple sources	Establish the process of maintaining an overview of multiple budgets from multiple sources
	Seek and use information on financial funding requirements	Awareness of the need to seek and use information on financial funding requirements	Understand the process of seeking and using information on financial funding requirements	Providing input during seeking and using information on financial funding requirements	Seek and use information on financial funding requirements	Develop the methodology of seeking and using information on financial funding requirements
	Ensure timely decision making with regard to financial shortfalls	Awareness of the need for timely decision making with regard to financial shortfalls	Understand the need for timely decision making with regard to financial shortfalls	Ensure timely decision making with regard to financial shortfalls	Analyse the process of ensuring timely decision making with regard to financial shortfalls	Develop the methodology to ensure timely decision making with regard to financial shortfalls
	Provide budget tracking for logistics activities according to established procedures	Awareness of the need for budget tracking for logistics activities according to established procedures	Understand the need for budget tracking for logistics activities according to established procedures	Provide budget tracking for logistics activities according to established procedures	Analyse budget tracking for logistics activities according to established procedures	Implement budget tracking for logistics activities according to established procedures
	Analyse financial statements and explain components of a balance sheet and income statement	Awareness of financial statements and the components of a balance sheet and income statement	Understand the need for analysing of financial statements and explaining components of a balance sheet and income statement	Provide input during the analysis of financial statements and explain components of a balance sheet and income statement	Analyse financial statements and explain components of a balance sheet and income statement	Strategically analyse financial statements and explain components of a balance sheet and income statement
	Create interactive decision support models that allow the development of multiple scenarios and demonstrate the sensitivity of multiple independent variables	Awareness of interactive decision support models that allow the development of multiple scenarios and demonstrating the sensitivity of multiple independent variables	Understand interactive decision support models that allow the development of multiple scenarios and demonstrating the sensitivity of multiple independent variables	Provide input on creating interactive decision support models that allow the development of multiple scenarios and demonstrating the sensitivity of multiple independent variables	Create interactive decision support models that allow the development of multiple scenarios and demonstrate the sensitivity of multiple independent variables	Develop interactive decision support models that allow the development of multiple scenarios and demonstrate the sensitivity of multiple independent variables

COMPETENCY	BEHAVIOURAL COMPETENCIES (including knowledge requirements)	Associate	Practitioner	Specialist	Professional	Leader
5.6 Manage financial activities (cont.)	Determine the success or failure of a business using financial accounting	Awareness that the success or failure of a business is determined using financial accounting	Examine the success or failure of a business using financial accounting	Describe the success or failure of a business using financial accounting	Determine the success or failure of a business using financial accounting	Illustrate the success or failure of a business using financial accounting
	Calculate the total system cost of delivering a product or service to the customer	Awareness of the total system cost of delivering a product or service to the customer	Understand the calculations of the total system cost of delivering a product or service to the customer	Calculate the total system cost of delivering a product or service to the customer	Evaluate the calculations of the total system cost of delivering a product or service to the customer	Strategically analyse the calculations of the total system cost of delivering a product or service to the customer
	Describe the key considerations for developing a business plan	Awareness of the key considerations for developing a business plan	Compare the key considerations for developing a business plan	Describe the key considerations for developing a business plan	Analyse the key considerations for developing a business plan	Establish the key considerations for developing a business plan
	Describe the structure against which business performance will be monitored (e.g. cost centres, chart of accounts)	Awareness of the structure against which business performance will be monitored (e.g. cost centres, chart of accounts)	Examine the structure against which business performance will be monitored (e.g. cost centres, chart of accounts)	Describe the structure against which business performance will be monitored (e.g. cost centres, chart of accounts)	Evaluate the structure against which business performance will be monitored (e.g. cost centres, chart of accounts)	Strategically analyse the structure against which business performance will be monitored (e.g. cost centres, chart of accounts)
	List key performance indicators used to monitor business performance (e.g. turnover, profitability)	Awareness of key performance indicators used to monitor business performance (e.g. turnover, profitability)	Interpret key performance indicators used to monitor business performance (e.g. turnover, profitability)	List key performance indicators used to monitor business performance (e.g. turnover, profitability)	Analyse key performance indicators used to monitor business performance (e.g. turnover, profitability)	Implement key performance indicators used to monitor business performance (e.g. turnover, profitability)
	List strategies for minimising the risk of fraudulent activity (e.g. reconciliation of purchase orders, receipts and payment approvals; dual signatory arrangements for funds transfers)	Awareness of strategies for minimising the risk of fraudulent activity (e.g. reconciliation of purchase orders, receipts and payment approvals; dual signatory arrangements for funds transfers)	Compare strategies for minimising the risk of fraudulent activity (e.g. reconciliation of purchase orders, receipts and payment approvals; dual signatory arrangements for funds transfers)	List strategies for minimising the risk of fraudulent activity (e.g. reconciliation of purchase orders, receipts and payment approvals; dual signatory arrangements for funds transfers)	Determine strategies for minimising the risk of fraudulent activity (e.g. reconciliation of purchase orders, receipts and payment approvals; dual signatory arrangements for funds transfers)	Implement strategies for minimising the risk of fraudulent activity (e.g. reconciliation of purchase orders, receipts and payment approvals; dual signatory arrangements for funds transfers)
	Describe investigative processes and options for confirming the existence of fraudulent activity (e.g. sample audits, forensic accounting services)	Awareness of investigative processes and options for confirming the existence of fraudulent activity (e.g. sample audits, forensic accounting services)	Understand investigative processes and options for confirming the existence of fraudulent activity (e.g. sample audits, forensic accounting services)	Describe investigative processes and options for confirming the existence of fraudulent activity (e.g. sample audits, forensic accounting services)	Evaluate investigative processes and options for confirming the existence of fraudulent activity (e.g. sample audits, forensic accounting services)	Implement investigative processes and options for confirming the existence of fraudulent activity (e.g. sample audits, forensic accounting services)
	Prepare funding applications for trusts and grants	Awareness of the process of preparing funding applications for trusts and grants	Understand the process of preparing funding applications for trusts and grants	Provide input on preparing funding applications for trusts and grants	Prepare funding applications for trusts and grants	Develop a process to prepare funding applications for trusts and grants
	Ensure financial transparency	Awareness of the need to ensure financial transparency	Understand the need to ensure financial transparency	Apply the process of ensuring financial transparency	Demonstrate the process of ensuring financial transparency	Ensure financial transparency
	Describe the general monetary value of medicines and equipment	Awareness of the general monetary value of medicines and equipment	Compare the general monetary value of medicines and equipment	Describe the general monetary value of medicines and equipment	Evaluate the general monetary value of medicines and equipment	Strategically analyse the general monetary value of medicines and equipment
	Secure financing and manage budgets to support distribution operations	Awareness of the need to secure financing and managing budgets to support distribution operations	Understand the need to secure financing and managing budgets to support distribution operations	Provide input to securing financing and managing budgets to support distribution operations	Secure financing and manage budgets to support distribution operations	Develop a process to secure financing and manage budgets to support distribution operations

COMPETENCY	BEHAVIOURAL COMPETENCIES (including knowledge requirements)	Associate	Practitioner	Specialist	Professional	Leader
**5.7 Oversee/ support human resources (e.g., recruitment, training, team management/ supervision)**						
**5.7.1 Manage staff recruitment process**	Demonstrate use of a system for staff recruitment, appraisals and monitoring	Awareness of a system for staff recruitment, appraisals and monitoring	Enable use of a system for staff recruitment, appraisals and monitoring	Apply use of a system for staff recruitment, appraisals and monitoring	Demonstrate use of a system for staff recruitment, appraisals and monitoring	Implement use of a system for staff recruitment, appraisals and monitoring
	Develop training and recruitment plans to fill any identified gaps and shortages	Awareness of training and recruitment plans to fill any identified gaps and shortages	Follow training and recruitment plans to fill any identified gaps and shortages	Apply training and recruitment plans to fill any identified gaps and shortages	Evaluate training and recruitment plans to fill any identified gaps and shortages	Develop training and recruitment plans to fill any identified gaps and shortages
	Create relevant and appropriate selection criteria for a defined role	Awareness of relevant and appropriate selection criteria for a defined role	Compare relevant and appropriate selection criteria for a defined role	Identify relevant and appropriate selection criteria for a defined role	Determine relevant and appropriate selection criteria for a defined role	Create relevant and appropriate selection criteria for a defined role
	Describe and use the standard selection documentation for recruitment	Awareness of the standard selection documentation for recruitment	Understand and follow the standard selection documentation for recruitment	Describe and use the standard selection documentation for recruitment	Analyse and demonstrate the standard selection documentation for recruitment	Develop and implement the standard selection documentation for recruitment
	List key issues impacting the size and membership of an interview panel (e.g. logistics, gender balance, absence of conflict of interest)	Awareness of key issues impacting the size and membership of an interview panel (e.g. logistics, gender balance, absence of conflict of interest)	Understand key issues impacting the size and membership of an interview panel (e.g. logistics, gender balance, absence of conflict of interest)	List key issues impacting the size and membership of an interview panel (e.g. logistics, gender balance, absence of conflict of interest)	Determine key issues impacting the size and membership of an interview panel (e.g. logistics, gender balance, absence of conflict of interest)	Strategically analyse key issues impacting the size and membership of an interview panel (e.g. logistics, gender balance, absence of conflict of interest)
	Formalise requirements for a fair, defensible and balanced interview process	Awareness of requirements for a fair, defensible and balanced interview process	Compare requirements for a fair, defensible and balanced interview process	Describe requirements for a fair, defensible and balanced interview process	Analyse requirements for a fair, defensible and balanced interview process	Formalise requirements for a fair, defensible and balanced interview process
	Describe and use the documentation maintained for each interviewed position	Awareness of the documentation maintained for each interviewed position	Understand and follow the documentation maintained for each interviewed position	Describe and use the documentation maintained for each interviewed position	Analyse and demonstrate the documentation maintained for each interviewed position	Develop and implement the documentation maintained for each interviewed position
	List due process for formalising an appointment that minimises the opportunity for untoward effects (e.g. appeal, conflict, resignation)	Awareness of due process for formalising an appointment that minimises the opportunity for untoward effects (e.g. appeal, conflict, resignation)	Understand due process for formalising an appointment that minimises the opportunity for untoward effects (e.g. appeal, conflict, resignation)	List due process for formalising an appointment that minimises the opportunity for untoward effects (e.g. appeal, conflict, resignation)	Determine due process for formalising an appointment that minimises the opportunity for untoward effects (e.g. appeal, conflict, resignation)	Develop and implement due process for formalising an appointment that minimises the opportunity for untoward effects (e.g. appeal, conflict, resignation)
	Develop job descriptions to obtain and retain skilled staff	Awareness of job descriptions to obtain and retain skilled staff	Understand job descriptions to obtain and retain skilled staff	Use job descriptions to obtain and retain skilled staff	Analyse job descriptions to obtain and retain skilled staff	Develop job descriptions to obtain and retain skilled staff

COMPETENCY	BEHAVIOURAL COMPETENCIES (including knowledge requirements)	Associate	Practitioner	Specialist	Professional	Leader
**5.7.1 Manage staff recruitment process (cont.)**	Consult with and involve relevant personnel in development and updating of role descriptions/duty statements that clarify the duties and responsibilities of positions	Awareness of the need to consult with and involve relevant personnel in development and updating of role descriptions/duty statements that clarify the duties and responsibilities of positions	Understand the process of consulting with and involving relevant personnel in development and updating of role descriptions/duty statements that clarify the duties and responsibilities of positions	Describe the process of consulting with and involving relevant personnel in development and updating of role descriptions/duty statements that clarify the duties and responsibilities of positions	Consult with and involve relevant personnel in development and updating of role descriptions/duty statements that clarify the duties and responsibilities of positions	Implement the process of consulting with and involving relevant personnel in development and updating of role descriptions/duty statements that clarify the duties and responsibilities of positions
	Orient new staff to the workplace, explaining standard operating systems and procedures	Awareness of the need to orient new staff to the workplace, explaining standard operating systems and procedures	Understand the process of orienting new staff to the workplace, explaining standard operating systems and procedures	Orient new staff to the workplace, explaining standard operating systems and procedures	Determine the process of orienting new staff to the workplace, explaining standard operating systems and procedures	Implement the process of orienting new staff to the workplace, explaining standard operating systems and procedures
	Prepare human resource plans to meet the future staffing needs of the organisation	Awareness of human resource plans to meet the future staffing needs of the organisation	Examine human resource plans to meet the future staffing needs of the organisation	Describe human resource plans to meet the future staffing needs of the organisation	Evaluate human resource plans to meet the future staffing needs of the organisation	Prepare human resource plans to meet the future staffing needs of the organisation
	Explain key features and measures used for monitoring a personnel retention strategy (e.g. recruitment costs, staff turnover, staff surveys)	Awareness of key features and measures used for monitoring a personnel retention strategy (e.g. recruitment costs, staff turnover, staff surveys)	Examine key features and measures used for monitoring a personnel retention strategy (e.g. recruitment costs, staff turnover, staff surveys)	Explain key features and measures used for monitoring a personnel retention strategy (e.g. recruitment costs, staff turnover, staff surveys)	Evaluate key features and measures used for monitoring a personnel retention strategy (e.g. recruitment costs, staff turnover, staff surveys)	Implement key features and measures used for monitoring a personnel retention strategy (e.g. recruitment costs, staff turnover, staff surveys)
	Describe the legislative issues impacting human resource policies and procedures	Awareness of the legislative issues impacting human resource policies and procedures	Understand the legislative issues impacting human resource policies and procedures	Describe the legislative issues impacting human resource policies and procedures	Determine the legislative issues impacting human resource policies and procedures	Strategically analyse the legislative issues impacting human resource policies and procedures
**5.7.2 Train staff**	Maintain a list of staff that require logistics system training (based on supportive supervision reports)	Awareness of the need to maintain a list of staff that require logistics system training (based on supportive supervision reports)	Follow the process of maintaining a list of staff that require logistics system training (based on supportive supervision reports)	Maintain a list of staff that require logistics system training (based on supportive supervision reports)	Determine the process of maintaining a list of staff that require logistics system training (based on supportive supervision reports)	Implement the process of maintaining a list of staff that require logistics system training (based on supportive supervision reports)
	Coordinate all training in managing the logistics system for all health facility staff	Awareness of the need to coordinate all training in managing the logistics system for all health facility staff	Understand the process of coordinating all training in managing the logistics system for all health facility staff	Describe the process of coordinating all training in managing the logistics system for all health facility staff	Coordinate all training in managing the logistics system for all health facility staff	Implement the process of coordinating all training in managing the logistics system for all health facility staff
	Promote participation in relevant learning and development opportunities	Awareness of the need for participation in relevant learning and development opportunities	Enable a culture of promoting participation in relevant learning and development opportunities	Apply a culture of promoting participation in relevant learning and development opportunities	Promote participation in relevant learning and development opportunities	Implement a culture of promoting participation in relevant learning and development opportunities
	Assist in training nurses and other health professionals in medicines ordering and storage procedures	Awareness of the need to assist in training nurses and other health professionals in medicines ordering and storage procedures	Understand the process of assisting in training nurses and other health professionals in medicines ordering and storage procedures	Assist in training nurses and other health professionals in medicines ordering and storage procedures	Demonstrate the process of assisting in training nurses and other health professionals in medicines ordering and storage procedures	Implement the process of assisting in training nurses and other health professionals in medicines ordering and storage procedures

COMPETENCY	BEHAVIOURAL COMPETENCIES (including knowledge requirements)	Associate	Practitioner	Specialist	Professional	Leader
5.7.2 Train staff (cont.)	Orient new doctors to the formulary and the systems and procedures of the medicines supply system	Awareness of the need to orient new doctors to the formulary and the systems and procedures of the medicines supply system	Understand the process of orienting new doctors to the formulary and the systems and procedures of the medicines supply system	Orient new doctors to the formulary and the systems and procedures of the medicines supply system	Demonstrate the process of orienting new doctors to the formulary and the systems and procedures of the medicines supply system	Implement the process of orienting new doctors to the formulary and the systems and procedures of the medicines supply system
	Provide on-the-job training to district health officials and hospital pharmacists as needed	Awareness of the need to provide on-the-job training to district health officials and hospital pharmacists as needed	Understand the process of providing on-the-job training to district health officials and hospital pharmacists as needed	Provide on-the-job training to district health officials and hospital pharmacists as needed	Determine the process of providing on-the-job training to district health officials and hospital pharmacists as needed	Implement the process of providing on-the-job training to district health officials and hospital pharmacists as needed
	Ensure staff have the necessary skills and understanding for safe practice in the event they need to fill a management role due to absence or illness	Awareness of the need to ensure staff have the necessary skills and understanding for safe practice in the event they need to fill a management role due to absence or illness	Understand the need to ensure staff have the necessary skills and understanding for safe practice in the event they need to fill a management role due to absence or illness	Ensure staff have the necessary skills and understanding for safe practice in the event they need to fill a management role due to absence or illness	Demonstrate the process of ensuring staff have the necessary skills and understanding for safe practice in the event they need to fill a management role due to absence or illness	Develop the process of ensuring staff have the necessary skills and understanding for safe practice in the event they need to fill a management role due to absence or illness
	Ensure all staff handling hazardous materials receive training on safety & compliance regulations	Awareness of the need to ensure all staff handling hazardous materials receive training on safety & compliance regulations	Understand the need to ensure all staff handling hazardous materials receive training on safety & compliance regulations	Ensure all staff handling hazardous materials receive training on safety & compliance regulations	Evaluate the process of staff handling hazardous materials receive training on safety & compliance regulations	Implement the process of all staff handling hazardous materials receive training on safety & compliance regulations
	Identify any staff needs at all levels of the supply chain	Awareness of any staff needs at all levels of the supply chain	Examine any staff needs at all levels of the supply chain	Identify any staff needs at all levels of the supply chain	Evaluate any staff needs at all levels of the supply chain	Strategically analyse any staff needs at all levels of the supply chain
	Discuss key factors important for initiating and/or sustaining the motivation of adult learners	Awareness of key factors important for initiating and/or sustaining the motivation of adult learners	Examine key factors important for initiating and/or sustaining the motivation of adult learners	Discuss key factors important for initiating and/or sustaining the motivation of adult learners	Analyse key factors important for initiating and/or sustaining the motivation of adult learners	Implement key factors important for initiating and/or sustaining the motivation of adult learners
	Discuss the variable learning needs and styles or modalities in adult learners (e.g. visual versus auditory)	Awareness of the variable learning needs and styles or modalities in adult learners (e.g. visual versus auditory)	Compare the variable learning needs and styles or modalities in adult learners (e.g. visual versus auditory)	Discuss the variable learning needs and styles or modalities in adult learners (e.g. visual versus auditory)	Evaluate the variable learning needs and styles or modalities in adult learners (e.g. visual versus auditory)	Strategically analyse the variable learning needs and styles or modalities in adult learners (e.g. visual versus auditory)
	Describe a range of strategies that could be used to reinforce and clarify educational content (e.g. tutorials or workshops, written materials, intranet- or internet-based resources)	Observe a range of strategies that could be used to reinforce and clarify educational content (e.g. tutorials or workshops, written materials, intranet- or internet-based resources)	Compare a range of strategies that could be used to reinforce and clarify educational content (e.g. tutorials or workshops, written materials, intranet- or internet-based resources)	Describe a range of strategies that could be used to reinforce and clarify educational content (e.g. tutorials or workshops, written materials, intranet- or internet-based resources)	Evaluate a range of strategies that could be used to reinforce and clarify educational content (e.g. tutorials or workshops, written materials, intranet- or internet-based resources)	Implement a range of strategies that could be used to reinforce and clarify educational content (e.g. tutorials or workshops, written materials, intranet- or internet-based resources)
5.7.3 Supervise staff	Use a performance management process with all personnel to ensure continuous improvement	Awareness of a performance management process with all personnel to ensure continuous improvement	Enable the use of a performance management process with all personnel to ensure continuous improvement	Use a performance management process with all personnel to ensure continuous improvement	Determine the use of a performance management process with all personnel to ensure continuous improvement	Implement the use of a performance management process with all personnel to ensure continuous improvement

COMPETENCY	BEHAVIOURAL COMPETENCIES (including knowledge requirements)	Associate	Practitioner	Specialist	Professional	Leader
5.7.3 Supervise staff (cont.)	Create an environment that is supportive of learning and professional development	Awareness of the need for an environment that is supportive of learning and professional development	Understand the need for an environment that is supportive of learning and professional development	Influence an environment that is supportive of learning and professional development	Determine an environment that is supportive of learning and professional development	Create an environment that is supportive of learning and professional development
	Describe the nature of the supervisory role, what is meant by direct supervision, and where responsibility for outputs and outcomes rests	Awareness of the nature of the supervisory role, what is meant by direct supervision, and where responsibility for outputs and outcomes rests	Interpret the nature of the supervisory role, what is meant by direct supervision, and where responsibility for outputs and outcomes rests	Describe the nature of the supervisory role, what is meant by direct supervision, and where responsibility for outputs and outcomes rests	Demonstrate the nature of the supervisory role, what is meant by direct supervision, and where responsibility for outputs and outcomes rests	Illustrate the nature of the supervisory role, what is meant by direct supervision, and where responsibility for outputs and outcomes rests
	Plan and conduct supervisory tours of dependent facilities	Awareness of the need to plan and conduct supervisory tours of dependent facilities	Understand the need to plan and conduct supervisory tours of dependent facilities	Describe the process of planning and conducting supervisory tours of dependent facilities	Plan and conduct supervisory tours of dependent facilities	Implement the process of planning and conducting supervisory tours of dependent facilities
	Identify situations where supervised personnel are experiencing difficulties in completing work activities and/or where a mandatory notification obligation exists	Awareness of the need to identify situations where supervised personnel are experiencing difficulties in completing work activities and/or where a mandatory notification obligation exists	Understand the need to identify situations where supervised personnel are experiencing difficulties in completing work activities and/or where a mandatory notification obligation exists	Identify situations where supervised personnel are experiencing difficulties in completing work activities and/or where a mandatory notification obligation exists	Analyse situations where supervised personnel are experiencing difficulties in completing work activities and/or where a mandatory notification obligation exists	Direct situations where supervised personnel are experiencing difficulties in completing work activities and/or where a mandatory notification obligation exists
	Describe signs/cues from supervised personnel that indicate additional guidance or support is needed (e.g. hesitancy, distress, seeks clarification from less authoritative sources)	Awareness of signs/cues from supervised personnel that indicate additional guidance or support is needed (e.g. hesitancy, distress, seeks clarification from less authoritative sources)	Interpret signs/cues from supervised personnel that indicate additional guidance or support is needed (e.g. hesitancy, distress, seeks clarification from less authoritative sources)	Describe signs/cues from supervised personnel that indicate additional guidance or support is needed (e.g. hesitancy, distress, seeks clarification from less authoritative sources)	Analyse signs/cues from supervised personnel that indicate additional guidance or support is needed (e.g. hesitancy, distress, seeks clarification from less authoritative sources)	Illustrate signs/cues from supervised personnel that indicate additional guidance or support is needed (e.g. hesitancy, distress, seeks clarification from less authoritative sources)
	Provide feedback, encouragement and support to team members	Awareness of the need to provide feedback, encouragement and support to team members	Understand the need to provide feedback, encouragement and support to team members	Provide feedback, encouragement and support to team members	Demonstrate a practise of providing feedback, encouragement and support to team members	Implement a practise of providing feedback, encouragement and support to team members
	Discuss in a constructive manner areas where performance should/could be improved and provide resources to support this	Awareness of the need to discuss in a constructive manner areas where performance should/could be improved and provide resources to support this	Understand the need to discuss in a constructive manner areas where performance should/could be improved and provide resources to support this	Discuss in a constructive manner areas where performance should/could be improved and provide resources to support this	Analyse areas where performance should/could be improved and provide resources to support this	Strategically analyse areas where performance should/could be improved and provide resources to support this
	Discuss expectations, achievements and contributions with personnel in a fair and equitable manner	Awareness of the need to discuss expectations, achievements and contributions with personnel in a fair and equitable manner	Understand the need to discuss expectations, achievements and contributions with personnel in a fair and equitable manner	Discuss expectations, achievements and contributions with personnel in a fair and equitable manner	Demonstrate the process of discussing expectations, achievements and contributions with personnel in a fair and equitable manner	Develop the process of discussing expectations, achievements and contributions with personnel in a fair and equitable manner

COMPETENCY	BEHAVIOURAL COMPETENCIES (including knowledge requirements)	Associate	Practitioner	Specialist	Professional	Leader
5.7.3 Supervise staff (cont.)	Support colleagues in creating a professional development plan and suggest ways the plan may be progressed through relevant training and/or experiential learning opportunities	Awareness of the need to support colleagues in creating a professional development plan and suggesting ways the plan may be progressed through relevant training and/or experiential learning opportunities	Understand the need to support colleagues in creating a professional development plan and suggesting ways the plan may be progressed through relevant training and/or experiential learning opportunities	Apply the process of supporting colleagues in creating a professional development plan and suggesting ways the plan may be progressed through relevant training and/or experiential learning opportunities	Support colleagues in creating a professional development plan and suggest ways the plan may be progressed through relevant training and/or experiential learning opportunities	Implement the process of supporting colleagues in creating a professional development plan and suggesting ways the plan may be progressed through relevant training and/or experiential learning opportunities
	Encourage and support high-potential employees to accept growth challenges outside their own areas	Awareness of the need to encourage and support high-potential employees to accept growth challenges outside their own areas	Understand the need to encourage and support high-potential employees to accept growth challenges outside their own areas	Encourage and support high-potential employees to accept growth challenges outside their own areas	Demonstrate a culture of encouraging and supporting high-potential employees to accept growth challenges outside their own areas	Implement a culture of encouraging and supporting high-potential employees to accept growth challenges outside their own areas
	Provide progression opportunities for employees who have prepared themselves through development	Awareness of the progression opportunities for employees who have prepared themselves through development	Understand the progression opportunities for employees who have prepared themselves through development	Provide progression opportunities for employees who have prepared themselves through development	Demonstrate the process of providing progression opportunities for employees who have prepared themselves through development	Develop the process of providing progression opportunities for employees who have prepared themselves through development
	Identify and deal with unproductive staff using an approved process in an ethical manner	Awareness of the need to identify and deal with unproductive staff using an approved process in an ethical manner	Understand the need to identify and deal with unproductive staff using an approved process in an ethical manner	Identify and deal with unproductive staff using an approved process in an ethical manner	Evaluate the process of identifying and dealing with unproductive staff using an approved process in an ethical manner	Develop and implement the process of identifying and dealing with unproductive staff using an approved process in an ethical manner
	Identify and address work issues contributing to impairment of personnel (e.g. excessive workload, conflict)	Awareness of the need to identify and address work issues contributing to impairment of personnel (e.g. excessive workload, conflict)	Understand the need to identify and address work issues contributing to impairment of personnel (e.g. excessive workload, conflict)	Identify and address work issues contributing to impairment of personnel (e.g. excessive workload, conflict)	Evaluate and address work issues contributing to impairment of personnel (e.g. excessive workload, conflict)	Strategically analyse and address work issues contributing to impairment of personnel (e.g. excessive workload, conflict)
	Explain the complaints management process	Basic operational knowledge of the complaints management process	Understand the complaints management process	Explain the complaints management process	Determine the complaints management process	Develop the complaints management process
	Describe situations where additional expertise should be sought (e.g. counselling in the event of workplace violence or in situations of diminished performance due to drug or alcohol abuse)	Awareness of the fact that there may be situations where additional expertise should be sought (e.g. counselling in the event of workplace violence or in situations of diminished performance due to drug or alcohol abuse)	Examine situations where additional expertise should be sought (e.g. counselling in the event of workplace violence or in situations of diminished performance due to drug or alcohol abuse)	Describe situations where additional expertise should be sought (e.g. counselling in the event of workplace violence or in situations of diminished performance due to drug or alcohol abuse)	Evaluate situations where additional expertise should be sought (e.g. counselling in the event of workplace violence or in situations of diminished performance due to drug or alcohol abuse)	Direct situations where additional expertise should be sought (e.g. counselling in the event of workplace violence or in situations of diminished performance due to drug or alcohol abuse)

COMPETENCY	BEHAVIOURAL COMPETENCIES (including knowledge requirements)	Associate	Practitioner	Specialist	Professional	Leader
5.7.4 Assessing HR systems	Acknowledge and respect different working styles	Awareness of different working styles	Understand different working styles	Acknowledge and respect different working styles	Demonstrate a culture of acknowledging and respecting different working styles	Implement a culture of acknowledging and respecting different working styles
	Ability to assess HR systems	Awareness of the need to assess HR systems	Understand the need to assess HR systems	Provide input on assessing HR systems	Ability to assess HR systems	Assess HR systems
5.8 Prepare for product supply during disasters and emergencies	Explain the process for dealing with disaster events	Awareness of the process for dealing with disaster events	Understand the process for dealing with disaster events	Explain the process for dealing with disaster events	Demonstrate the process for dealing with disaster events	Develop the process for dealing with disaster events
	Communicate with the national disaster planning team	Awareness of the need to communicate with the national disaster planning team	Understand the need to communicate with the national disaster planning team	Describe the process of communicating with the national disaster planning team	Communicate with the national disaster planning team	Establish the process of communicating with the national disaster planning team
	Describe the logistics requirements for emergency and disaster supply	Awareness of the logistics requirements for emergency and disaster supply	Understand the logistics requirements for emergency and disaster supply	Describe the logistics requirements for emergency and disaster supply	Analyse the logistics requirements for emergency and disaster supply	Develop the logistics requirements for emergency and disaster supply
	Describe the logistics planning process for emergency and disaster supply	Execute the logistics planning process for emergency and disaster supply	Understand the logistics planning process for emergency and disaster supply	Describe the logistics planning process for emergency and disaster supply	Determine the logistics planning process for emergency and disaster supply	Develop the logistics planning process for emergency and disaster supply
	Explain the importance of a needs assessment for emergency and disaster supply	Awareness of the importance of a needs assessment for emergency and disaster supply	Understand the importance of a needs assessment for emergency and disaster supply	Explain the importance of a needs assessment for emergency and disaster supply	Demonstrate the importance of a needs assessment for emergency and disaster supply	Illustrate the importance of a needs assessment for emergency and disaster supply
	Explain the importance of assessing local capacity before the emergency or disaster supply	Awareness of the importance of assessing local capacity before the emergency or disaster supply	Understand the importance of assessing local capacity before the emergency or disaster supply	Explain the importance of assessing local capacity before the emergency or disaster supply	Demonstrate the importance of assessing local capacity before the emergency or disaster supply	Illustrate the importance of assessing local capacity before the emergency or disaster supply
	List the requirements for hazardous and emergency or disaster supply commodities	Awareness of the requirements for hazardous and emergency or disaster supply commodities	Understand the requirements for hazardous and emergency or disaster supply commodities	List the requirements for hazardous and emergency or disaster supply commodities	Analyse the requirements for hazardous and emergency or disaster supply commodities	Assemble the requirements for hazardous and emergency or disaster supply commodities
	Explain the sources and procurement process for emergency or disaster supply	Basic operational knowledge of the sources and procurement process for emergency or disaster supply	Compare the sources and procurement process for emergency or disaster supply	Explain the sources and procurement process for emergency or disaster supply	Determine the sources and procurement process for emergency or disaster supply	Strategically analyse the sources and procurement process for emergency or disaster supply
	List the process of managing medical supplies for emergency or disaster supply	Awareness of the process of managing medical supplies for emergency or disaster supply	Understand the process of managing medical supplies for emergency or disaster supply	List the process of managing medical supplies for emergency or disaster supply	Analyse the process of managing medical supplies for emergency or disaster supply	Direct the process of managing medical supplies for emergency or disaster supply
	Explain why information management and transparency is important during emergency or disaster supply	Awareness of why information management and transparency is important during emergency or disaster supply	Show why information management and transparency is important during emergency or disaster supply	Describe why information management and transparency is important during emergency or disaster supply	Demonstrate why information management and transparency is important during emergency or disaster supply	Illustrate why information management and transparency is important during emergency or disaster supply

6. Professional and Personal

COMPETENCY	BEHAVIOURAL COMPETENCES (including knowledge requirements)	Associate	Practitioner	Specialist	Professional	Leader
6.1 Demonstrate generic skills (e.g. literacy, numeracy, technology)						
6.1.1 Exhibit high understanding of literacy and numeracy	Speak clearly and confidently, organise information in a logical manner, and consider voice tone and pace	Speak clearly and confidently, organise information in a logical manner, and consider voice tone and pace	Speak clearly and confidently, organise information in a logical manner, and consider voice tone and pace	Speak clearly and confidently, organise information in a logical manner, and consider voice tone and pace	Encourage speaking clearly and confidently, organising information in a logical manner, and considering voice tone and pace	Establish a culture of speaking clearly and confidently, organising information in a logical manner, and considering voice tone and pace
	Respond to verbal messages and other cues (active listening)	Respond to verbal messages and other cues (active listening)	Respond to verbal messages and other cues (active listening)	Respond to verbal messages and other cues (active listening)	Demonstrate responding to verbal messages and other cues (active listening)	Demonstrate responding to verbal messages and other cues (active listening)
	Express ideas and opinions clearly in written and verbal form	Express ideas and opinions clearly in written and verbal form	Express ideas and opinions clearly in written and verbal form	Express ideas and opinions clearly in written and verbal form	Demonstrate expressing ideas and opinions clearly in written and verbal form	Establish a culture of expressing ideas and opinions clearly in written and verbal form
	Communicate information accurately, concisely and confidently in writing and verbally	Communicate information accurately, concisely and confidently in writing and verbally	Communicate information accurately, concisely and confidently in writing and verbally	Communicate information accurately, concisely and confidently in writing and verbally	Demonstrate communicating information accurately, concisely and confidently in writing and verbally	Demonstrate communicating information accurately, concisely and confidently in writing and verbally
	Practice a level of mathematics suitable to the job held	Practice a level of mathematics suitable to the job held	Practice a level of mathematics suitable to the job held	Practice a level of mathematics suitable to the job held	Demonstrate a level of mathematics suitable to the job held	Demonstrate an in high level of mathematics suitable to the job held
	Demonstrate knowledge of visual presentation techniques, including charting, histograms and flow sheets	Awareness of visual presentation techniques, including charting, histograms and flow sheets	Compare visual presentation techniques, including charting, histograms and flow sheets	Describe visual presentation techniques, including charting, histograms and flow sheets	Demonstrate knowledge of visual presentation techniques, including charting, histograms and flow sheets	In depth knowledge of visual presentation techniques, including charting, histograms and flow sheets
	Read and comprehend at a level necessary to properly complete duties of the position	Read and comprehend at a level necessary to properly complete duties of the position	Read and comprehend at a level necessary to properly complete duties of the position	Read and comprehend at a level necessary to properly complete duties of the position	Demonstrate reading and comprehending at a level necessary to properly complete duties of the position	Demonstrate effective reading and comprehension at a level necessary to properly complete duties of the position
	Interpret written information in documents, such as reports, SOPs, LMIS forms, graphs, calendars, schedules, notices and directions	Awareness of the need to interpret written information in documents, such as reports, SOPs, LMIS forms, graphs, calendars, schedules, notices and directions	Understand the need to interpret written information in documents, such as reports, SOPs, LMIS forms, graphs, calendars, schedules, notices and directions	Interpret written information in documents, such as reports, SOPs, LMIS forms, graphs, calendars, schedules, notices and directions	Demonstrate the process of interpreting written information in documents, such as reports, SOPs, LMIS forms, graphs, calendars, schedules, notices and directions	Develop the methodology to interpret written information in documents, such as reports, SOPs, LMIS forms, graphs, calendars, schedules, notices and directions

COMPETENCY	BEHAVIOURAL COMPETENCES (including knowledge requirements)	Associate	Practitioner	Specialist	Professional	Leader
6.1.1 Exhibit high understanding of literacy and numeracy (cont.)	Pay attention to detail and identify the main ideas, detect inconsistencies and identify missing information in documents	Awareness of the need to paying attention to detail and identifying the main ideas, detecting inconsistencies and identifying missing information in documents	Understand the need to pay attention to detail and identify the main ideas, detect inconsistencies and identify missing information in documents	Pay attention to detail and identify the main ideas, detect inconsistencies and identify missing information in documents	Demonstrate paying attention to detail and identifying the main ideas, detecting inconsistencies and identifying missing information in documents	Demonstrate paying attention to detail and identifying the main ideas, detecting inconsistencies and identifying missing information in documents
	Apply computer skills — such as using Word, Excel, PowerPoint and the internet — suitable to the job level	Execute computer skills — such as using Word, Excel, PowerPoint and the internet — suitable to the job level	Enable computer skills — such as using Word, Excel, PowerPoint and the internet — suitable to the job level	Apply computer skills — such as using Word, Excel, PowerPoint and the internet — suitable to the job level	Demonstrate computer skills — such as using Word, Excel, PowerPoint and the internet — suitable to the job level	Implement computer skills — such as using Word, Excel, PowerPoint and the internet — suitable to the job level
6.2 Demonstrate strong communication skills	Be truthful and trustworthy, and supply accurate information at all times	Be truthful and trustworthy, and supply accurate information at all times	Be truthful and trustworthy, and supply accurate information at all times	Be truthful and trustworthy, and supply accurate information at all times	Demonstrate being truthful and trustworthy, and supplying accurate information at all times	Develop a culture of being truthful and trustworthy, and supplying accurate information at all times
	Work as part of a workplace team	Realize the need to work as part of a workplace team	Enable working as part of a workplace team	Work as part of a workplace team	Demonstrate working as part of a workplace team	Develop a culture of working as part of a workplace team
	Describe vocabulary and communication style and form for both written and verbal communication that is appropriate for the situation, audience and material being communicated (e.g. avoid unnecessary jargon, clearly explain medical and SCM terminology)	Awareness of vocabulary and communication style and form for both written and verbal communication that is appropriate for the situation, audience and material being communicated (e.g. avoid unnecessary jargon, clearly explain medical and SCM terminology)	Understand vocabulary and communication style and form for both written and verbal communication that is appropriate for the situation, audience and material being communicated (e.g. avoid unnecessary jargon, clearly explain medical and SCM terminology)	Describe vocabulary and communication style and form for both written and verbal communication that is appropriate for the situation, audience and material being communicated (e.g. avoid unnecessary jargon, clearly explain medical and SCM terminology)	Determine vocabulary and communication style and form for both written and verbal communication that is appropriate for the situation, audience and material being communicated (e.g. avoid unnecessary jargon, clearly explain medical and SCM terminology)	Demonstrate vocabulary and communication style and form for both written and verbal communication that is appropriate for the situation, audience and material being communicated (e.g. avoid unnecessary jargon, clearly explain medical and SCM terminology)
	Encourage open communication within constraints of confidentiality	Awareness of open communication within constraints of confidentiality	Enable open communication within constraints of confidentiality	Encourage open communication within constraints of confidentiality	Demonstrate open communication within constraints of confidentiality	Develop a culture of open communication within constraints of confidentiality
	Organise communications about logistics	Awareness of communications about logistics	Participate in the organisation of communications about logistics	Organise communications about logistics	Analyse communications about logistics	Define the process of communicating about logistics
	Participate in meetings, expressing one's opinions, being aware of others' needs, and being appropriately assertive when required	Participate in meetings, expressing one's opinions, being aware of others' needs, and being appropriately assertive when required	Enable participation in meetings, expressing one's opinions, being aware of others' needs, and being appropriately assertive when required	Encourage participation in meetings, expressing one's opinions, being aware of others' needs, and being appropriately assertive when required	Demonstrate participation in meetings, expressing one's opinions, being aware of others' needs, and being appropriately assertive when required	Develop a culture of participating in meetings, expressing one's opinions, being aware of others' needs, and being appropriately assertive when required
	Demonstrate perspective into the point of view of others, understanding their needs and goals	Awareness of perspective into the point of view of others, understanding their needs and goals	Show perspective into the point of view of others, understanding their needs and goals	Apply perspective into the point of view of others, understanding their needs and goals	Demonstrate perspective into the point of view of others, understanding their needs and goals	Demonstrate perspective into the point of view of others, understanding their needs and goals
	Build trust, rapport and credibility with others	Build trust, rapport and credibility with others	Enable the practise of building trust, rapport and credibility with others	Encourage building trust, rapport and credibility with others	Demonstrate the practise of building trust, rapport and credibility with others	Develop the culture of building trust, rapport and credibility with others

COMPETENCY	BEHAVIOURAL COMPETENCIES (including knowledge requirements)	Associate	Practitioner	Specialist	Professional	Leader
6.2 Demonstrate strong communication skills (cont.)	Anticipate the communication needs and concerns of others and respond to them	Awareness of the communication needs and concerns of others and respond to them	Understand the communication needs and concerns of others and respond to them	Anticipate the communication needs and concerns of others and respond to them	Determine the communication needs and concerns of others and respond to them	Establish the communication needs and concerns of others and respond to them
	Avoid conflict between work and personal interests	Awareness of the need to avoid conflict between work and personal interests	Understand the need to avoid conflict between work and personal interests	Avoid conflict between work and personal interests	Demonstrate avoiding conflict between work and personal interests	Develop a culture of avoiding conflict between work and personal interests
	Practice meaningful two-way communication	Practice meaningful two-way communication	Enable meaningful two-way communication	Encourage meaningful two-way communication	Demonstrate the practice of meaningful two-way communication	Develop a culture of meaningful two-way communication
	Influence others by persuasively presenting thoughts and ideas	Realize that others are influenced by persuasively presenting thoughts and ideas	Show influence on others by persuasively presenting thoughts and ideas	Influence others by persuasively presenting thoughts and ideas	Demonstrate influence on others by persuasively presenting thoughts and ideas	Establish influence on others by persuasively presenting thoughts and ideas
	Apply assertiveness skills to deal with unreasonable requests and/ or refusals that would compromise practice or consumer care	Awareness of the need for assertiveness skills to deal with unreasonable requests and/ or refusals that would compromise practice or consumer care	Show assertiveness skills to deal with unreasonable requests and/ or refusals that would compromise practice or consumer care	Apply assertiveness skills to deal with unreasonable requests and/ or refusals that would compromise practice or consumer care	Demonstrate assertiveness skills to deal with unreasonable requests and/ or refusals that would compromise practice or consumer care	Demonstrate effective assertiveness skills to deal with unreasonable requests and/ or refusals that would compromise practice or consumer care
	Maintain a positive, supportive and appreciative attitude	Maintain a positive, supportive and appreciative attitude	Maintain a positive, supportive and appreciative attitude	Maintain a positive, supportive and appreciative attitude	Demonstrate a positive, supportive and appreciative attitude	Demonstrate a positive, supportive and appreciative attitude
	Demonstrate the ability to balance the organisation's needs and the customer's needs	Awareness of the need to balance the organisation's needs and the customer's needs	Show the ability to balance the organisation's needs and the customer's needs	Apply the ability to balance the organisation's needs and the customer's needs	Demonstrate the ability to balance the organisation's needs and the customer's needs	Demonstrate the ability to balance the organisation's needs and the customer's needs
	Demonstrate self-control by maintaining composure and keeping emotions in check, even in difficult situations; deal calmly and effectively with stressful situations	Execute self-control by maintaining composure and keeping emotions in check, even in difficult situations; deal calmly and effectively with stressful situations	Show self-control by maintaining composure and keeping emotions in check, even in difficult situations; deal calmly and effectively with stressful situations	Use self-control by maintaining composure and keeping emotions in check, even in difficult situations; deal calmly and effectively with stressful situations	Demonstrate self-control by maintaining composure and keeping emotions in check, even in difficult situations; deal calmly and effectively with stressful situations	Establish self-control by maintaining composure and keeping emotions in check, even in difficult situations; deal calmly and effectively with stressful situations
	Express opinions and provide information in written and/or verbal form in a manner that does not elicit concern, anger or other adverse response	Express opinions and provide information in written and/or verbal form in a manner that does not elicit concern, anger or other adverse response	Enable expressing of opinions and providing information in written and/or verbal form in a manner that does not elicit concern, anger or other adverse response	Encourage the expressing of opinions and providing information in written and/or verbal form in a manner that does not elicit concern, anger or other adverse response	Demonstrate the expressing of opinions and providing information in written and/or verbal form in a manner that does not elicit concern, anger or other adverse response	Demonstrate the expressing of opinions and providing information in written and/or verbal form in a manner that does not elicit concern, anger or other adverse response
	Demonstrate the use of a systematic process for following up that demonstrates written reports have been received and understood	Basic operational knowledge of a systematic process for following up that demonstrates written reports have been received and understood	Enable the use of a systematic process for following up that demonstrates written reports have been received and understood	Apply the use of a systematic process for following up that demonstrates written reports have been received and understood	Demonstrate the use of a systematic process for following up that demonstrates written reports have been received and understood	Develop a systematic process for following up that demonstrates written reports have been received and understood

COMPETENCY	BEHAVIOURAL COMPETENCIES (including knowledge requirements)	Associate	Practitioner	Specialist	Professional	Leader
6.2 Demonstrate strong communication skills (cont.)	Describe the means by which responses to input to the work environment are monitored	Basic operational knowledge of the means by which responses to input to the work environment are monitored	Understand the means by which responses to input to the work environment are monitored	Describe the means by which responses to input to the work environment are monitored	Evaluate the means by which responses to input to the work environment are monitored	Implement the means by which responses to input to the work environment are monitored
	Seek practical ways to overcome barriers to communication	Seek practical ways to overcome barriers to communication	Seek practical ways to overcome barriers to communication	Seek practical ways to overcome barriers to communication	Seek practical ways to overcome barriers to communication	Seek practical ways to overcome barriers to communication
	Ensure language representation (e.g. interpreters) at meetings when appropriate	Awareness of the need for language representation (e.g. interpreters) at meetings when appropriate	Understand the need to ensuring language representation (e.g. interpreters) at meetings when appropriate	Identify language representation (e.g. interpreters) at meetings when appropriate	Ensure language representation (e.g. interpreters) at meetings when appropriate	Demonstrate language representation (e.g. interpreters) at meetings when appropriate
	Tackle difficult situations and resolve disputes between staff	Realize that difficult situations and disputes between staff need to be tackled and resolved	Interpret difficult situations and disputes between staff that need to be tackled and resolved	Tackle difficult situations and resolve disputes between staff	Demonstrate tackling difficult situations and resolving disputes between staff	Develop methodology for tackling difficult situations and resolving disputes between staff
	Give both positive and negative feedback sensitively	Realize the need to give both positive and negative feedback sensitively	Enable giving both positive and negative feedback sensitively	Give both positive and negative feedback sensitively	Demonstrate giving both positive and negative feedback sensitively	Establish a culture of giving both positive and negative feedback sensitively
	Maintain and improve communication	Awareness of the need to maintain and improve communication	Understand the need to maintain and improve communication	Maintain and improve communication	Demonstrate maintaining and improving communication	Establish a culture of maintaining and improving communication
6.2.1 Practice cultural awareness	Demonstrate cultural awareness and sensitivity, treating all people with fairness, respect and dignity	Awareness of the need for cultural awareness and sensitivity, treating all people with fairness, respect and dignity	Understand the need for cultural awareness and sensitivity, treating all people with fairness, respect and dignity	Describe cultural awareness and sensitivity, treating all people with fairness, respect and dignity	Demonstrate cultural awareness and sensitivity, treating all people with fairness, respect and dignity	Implement cultural awareness and sensitivity, treating all people with fairness, respect and dignity
	Implement anti-discriminatory practices in the organisation, including HR and disciplinary procedures	Awareness of the anti-discriminatory practices in the organisation, including HR and disciplinary procedures	Enable anti-discriminatory practices in the organisation, including HR and disciplinary procedures	Describe anti-discriminatory practices in the organisation, including HR and disciplinary procedures	Demonstrate anti-discriminatory practices in the organisation, including HR and disciplinary procedures	Implement anti-discriminatory practices in the organisation, including HR and disciplinary procedures
	Promote cultural sensitivity, equality and fairness at all levels of the organisation	Awareness of the need for cultural sensitivity, equality and fairness at all levels of the organisation	Enable cultural sensitivity, equality and fairness at all levels of the organisation	Establish cultural sensitivity, equality and fairness at all levels of the organisation	Demonstrate cultural sensitivity, equality and fairness at all levels of the organisation	Promote cultural sensitivity, equality and fairness at all levels of the organisation
	Challenge discriminatory behaviour directly and sensitively	Challenge discriminatory behaviour directly and sensitively	Challenge discriminatory behaviour directly and sensitively	Challenge discriminatory behaviour directly and sensitively	Demonstrate challenging discriminatory behaviour directly and sensitively	Establish a culture of challenging discriminatory behaviour directly and sensitively
	Act in a non-discriminatory way toward individuals and groups	Act in a non-discriminatory way toward individuals and groups	Act in a non-discriminatory way toward individuals and groups	Act in a non-discriminatory way toward individuals and groups	Demonstrate acting in a non-discriminatory way toward individuals and groups	Establish a culture of acting in a non-discriminatory way toward individuals and groups
	Integrate cultural awareness in learning and development approaches	Awareness of the need for integrating cultural awareness in learning and development approaches	Enable integrating cultural awareness in learning and development approaches	Integrate cultural awareness in learning and development approaches	Demonstrate the process of integrating cultural awareness in learning and development approaches	Implement the process of integrating cultural awareness in learning and development approaches

COMPETENCY	BEHAVIOURAL COMPETENCIES (including knowledge requirements)	Associate	Practitioner	Specialist	Professional	Leader
6.2.1 Practice cultural awareness (cont.)	Avoid stereotypical responses by examining one's own behaviour and bias	Avoid stereotypical responses by examining one's own behaviour and bias	Avoid stereotypical responses by examining one's own behaviour and bias	Avoid stereotypical responses by examining one's own behaviour and bias	Demonstrate avoiding stereotypical responses by examining one's own behaviour and bias	Establish a culture of avoiding stereotypical responses by examining one's own behaviour and bias
	Manage cultural diversity in teams and make the most of differences	Awareness of the need to manage cultural diversity in teams and make the most of differences	Enable managing cultural diversity in teams and make the most of differences	Manage cultural diversity in teams and make the most of differences	Demonstrate managing cultural diversity in teams and make the most of differences	Establish a culture of managing cultural diversity in teams and make the most of differences
	Describe strategies and/or resources for communicating effectively with people from different cultural backgrounds	Awareness of strategies and/ or resources for communicating effectively with people from different cultural backgrounds	Compare strategies and/or resources for communicating effectively with people from different cultural backgrounds	Describe strategies and/or resources for communicating effectively with people from different cultural backgrounds	Demonstrate the use of strategies and/or resources for communicating effectively with people from different cultural backgrounds	Develop strategies and/or resources for communicating effectively with people from different cultural backgrounds
	Elicit information relating to values, beliefs and cultural backgrounds of consumers that may influence the way professional services are provided	Awareness of the need to elicit information relating to values, beliefs and cultural backgrounds of consumers that may influence the way professional services are provided	Enable the process to elicit information relating to values, beliefs and cultural backgrounds of consumers that may influence the way professional services are provided	Elicit information relating to values, beliefs and cultural backgrounds of consumers that may influence the way professional services are provided	Supply information relating to values, beliefs and cultural backgrounds of consumers that may influence the way professional services are provided	Assemble information relating to values, beliefs and cultural backgrounds of consumers that may influence the way professional services are provided
6.3 Utilise problem-solving skills	Examine information to solve problems in a sensitive and ethical manner	Awareness of information to solve problems in a sensitive and ethical manner	Examine information to solve problems in a sensitive and ethical manner	Identify information to solve problems in a sensitive and ethical manner	Evaluate information to solve problems in a sensitive and ethical manner	Strategically analyse information to solve problems in a sensitive and ethical manner
	Manage day to day and complex problems in a timely manner	Awareness of the need to manage day to day and complex problems in a timely manner	Understand the need to manage day to day and complex problems in a timely manner	Manage day to day and complex problems in a timely manner	Demonstrate managing day to day and complex problems in a timely manner	Establish a culture of managing day to day and complex problems in a timely manner
	Explain problem situations and their step-by-step transformation based on planning and reasoning, without apportioning blame	Awareness of need to explain problem situations and their step-by-step transformation based on planning and reasoning, without apportioning blame	Understand the need to explain problem situations and their step-by-step transformation based on planning and reasoning, without apportioning blame	Explain problem situations and their step-by-step transformation based on planning and reasoning, without apportioning blame	Evaluate problem situations and their step-by-step transformation based on planning and reasoning, without apportioning blame	Strategically analyse problem situations and their step-by-step transformation based on planning and reasoning, without apportioning blame
	Critically review, analyse, synthesise, compare and interpret information; draw conclusions from relevant and/or missing information; and understand the relationship among facts and apply this understanding when solving problems	Awareness of the need to critically review, analyse, synthesise, compare and interpret information; draw conclusions from relevant and/or missing information; and understand the relationship among facts and apply this understanding when solving problems	Understand the need to critically review, analyse, synthesise, compare and interpret information; draw conclusions from relevant and/or missing information; and understand the relationship among facts and apply this understanding when solving problems	Critically review, analyse, synthesise, compare and interpret information; draw conclusions from relevant and/or missing information; and understand the relationship among facts and apply this understanding when solving problems	Demonstrate the process of critically reviewing, analysing, synthesising, comparing and interpreting information; drawing conclusions from relevant and/or missing information; and understanding the relationship among facts and apply this understanding when solving problems	Develop the process of critically reviewing, analysing, synthesising, comparing and interpreting information; drawing conclusions from relevant and/or missing information; and understanding the relationship among facts and apply this understanding when solving problems

COMPETENCY	BEHAVIOURAL COMPETENCIES (including knowledge requirements)	Associate	Practitioner	Specialist	Professional	Leader
6.3 Utilise problem-solving skills (cont.)	Describe a range of possible approaches/ strategies that are effective for resolving conflict in the workplace (e.g. negotiation, collaborative problem-solving, mediation, arbitration)	Awareness of a range of possible approaches/ strategies that are effective for resolving conflict in the workplace (e.g. negotiation, collaborative problem-solving, mediation, arbitration)	Compare a range of possible approaches/ strategies that are effective for resolving conflict in the workplace (e.g. negotiation, collaborative problem-solving, mediation, arbitration)	Describe a range of possible approaches/ strategies that are effective for resolving conflict in the workplace (e.g. negotiation, collaborative problem-solving, mediation, arbitration)	Evaluate a range of possible approaches/ strategies that are effective for resolving conflict in the workplace (e.g. negotiation, collaborative problem-solving, mediation, arbitration)	Develop a range of possible approaches/ strategies that are effective for resolving conflict in the workplace (e.g. negotiation, collaborative problem-solving, mediation, arbitration)
	Create an open environment that encourages people to work together	Realise an open environment that encourages people to work together	Enable an open environment that encourages people to work together	Use an open environment that encourages people to work together	Evaluate an open environment that encourages people to work together	Create an open environment that encourages people to work together
	Resolve conflicts as they arise	Awareness of the need to resolve conflicts as they arise	Understand the need to resolve conflicts as they arise	Resolve conflicts as they arise	Demonstrate resolving conflicts as they arise	Establish a culture of resolving conflicts as they arise
	Demonstrate creativity through questioning, attempting to improve on ideas, applying other experiences and working toward action in problem solving	Execute creativity through questioning, attempting to improve on ideas, applying other experiences and working toward action in problem solving	Enable creativity through questioning, attempting to improve on ideas, applying other experiences and working toward action in problem solving	Use creativity through questioning, attempting to improve on ideas, applying other experiences and working toward action in problem solving	Demonstrate creativity through questioning, attempting to improve on ideas, applying other experiences and working toward action in problem solving	Implement creativity through questioning, attempting to improve on ideas, applying other experiences and working toward action in problem solving
	Break down facts and thoughts into strengths and weaknesses	Awareness that one can break down facts and thoughts into strengths and weaknesses	Understand that one can break down facts and thoughts into strengths and weaknesses	Break down facts and thoughts into strengths and weaknesses	Analyse facts and thoughts into strengths and weaknesses	Strategically analyse facts and thoughts into strengths and weaknesses
	Think in a careful way to solve problems, analyse data, and recall and apply information, involving others	Awareness of the need to think in a careful way to solve problems, analyse data, and recall and apply information, involving others	Enable thinking in a careful way to solve problems, analyse data, and recall and apply information, involving others	Think in a careful way to solve problems, analyse data, and recall and apply information, involving others	Demonstrate thinking in a careful way to solve problems, analyse data, and recall and apply information, involving others	Establish a culture of thinking in a careful way to solve problems, analyse data, and recall and apply information, involving others
	Practice goal-directed thinking and action in situations in which no routine solutions exist	Awareness of goal-directed thinking and action in situations in which no routine solutions exist	Enable goal-directed thinking and action in situations in which no routine solutions exist	Practice goal-directed thinking and action in situations in which no routine solutions exist	Demonstrate goal-directed thinking and action in situations in which no routine solutions exist	Demonstrate goal-directed thinking and action in situations in which no routine solutions exist
	Maintain appropriate ethical and moral standards in resolving problems	Awareness of the need to maintain appropriate ethical and moral standards in resolving problems	Understand the need to maintain appropriate ethical and moral standards in resolving problems	Maintain appropriate ethical and moral standards in resolving problems	Demonstrate maintaining appropriate ethical and moral standards in resolving problems	Establish a culture of maintaining appropriate ethical and moral standards in resolving problems
	Choose between alternative courses of action, using cognitive processes such as memory, thinking and evaluation	Awareness of alternative courses of action, using cognitive processes such as memory, thinking and evaluation	Compare alternative courses of action, using cognitive processes such as memory, thinking and evaluation	Choose between alternative courses of action, using cognitive processes such as memory, thinking and evaluation	Analyse alternative courses of action, using cognitive processes such as memory, thinking and evaluation	Strategically analyse alternative courses of action, using cognitive processes such as memory, thinking and evaluation
	Map likely consequences of decisions to choose the best course of action	Awareness of the process to map likely consequences of decisions to choose the best course of action	Understand how to map likely consequences of decisions to choose the best course of action	Map likely consequences of decisions to choose the best course of action	Demonstrate mapping likely consequences of decisions to choose the best course of action	Develop a methodology for mapping likely consequences of decisions to choose the best course of action
	Ask other people to help with solving problems	Awareness of the need to ask other people to help with solving problems	Understand how to ask other people to help with solving problems	Ask other people to help with solving problems	Demonstrate asking other people to help with solving problems	Establish the culture of asking other people to help with solving problems

COMPETENCY	BEHAVIOURAL COMPETENCIES (including knowledge requirements)	Associate	Practitioner	Specialist	Professional	Leader
**6.3 Utilise problem-solving skills (cont.)**	Use difficult or unusual situations to develop unique approaches and useful solutions	Realise that difficult or unusual situations can be used to develop unique approaches and useful solutions	Examine difficult or unusual situations to develop unique approaches and useful solutions	Use difficult or unusual situations to develop unique approaches and useful solutions	Evaluate difficult or unusual situations to develop unique approaches and useful solutions	Strategically analyse difficult or unusual situations to develop unique approaches and useful solutions
	Commit to a solution in a timely manner, and develop a realistic approach for applying the chosen solution; evaluate the outcome of the solution to see if further action is needed, and identify lessons learned	Awareness of the need to commit to a solution in a timely manner, and develop a realistic approach for applying the chosen solution; evaluate the outcome of the solution to see if further action is needed, and identify lessons learned	Understand the need to commit to a solution in a timely manner, and develop a realistic approach for applying the chosen solution; evaluate the outcome of the solution to see if further action is needed, and identify lessons learned	Commit to a solution in a timely manner, and develop a realistic approach for applying the chosen solution; evaluate the outcome of the solution to see if further action is needed, and identify lessons learned	Demonstrate committing to a solution in a timely manner, and developing a realistic approach for applying the chosen solution; evaluating the outcome of the solution to see if further action is needed, and identify lessons learned	Develop the methodology for committing to a solution in a timely manner, and developing a realistic approach for applying the chosen solution; evaluating the outcome of the solution to see if further action is needed, and identify lessons learned
	Describe the impact of conflict in the workplace (e.g. tension, low morale, absenteeism, system or service failure, aggressive or uncooperative behaviours)	Awareness of the impact of conflict in the workplace (e.g. tension, low morale, absenteeism, system or service failure, aggressive or uncooperative behaviours)	Examine the impact of conflict in the workplace (e.g. tension, low morale, absenteeism, system or service failure, aggressive or uncooperative behaviours)	Describe the impact of conflict in the workplace (e.g. tension, low morale, absenteeism, system or service failure, aggressive or uncooperative behaviours)	Evaluate the impact of conflict in the workplace (e.g. tension, low morale, absenteeism, system or service failure, aggressive or uncooperative behaviours)	Strategically analyse the impact of conflict in the workplace (e.g. tension, low morale, absenteeism, system or service failure, aggressive or uncooperative behaviours)
	Follow up on problems to ensure they are fixed	Execute the practise of following up on problems to ensure they are fixed	Enable the practise of following up on problems to ensure they are fixed	Follow up on problems to ensure they are fixed	Demonstrate the practise of following up on problems to ensure they are fixed	Implement the practise of following up on problems to ensure they are fixed
	Describe situations where referral is warranted (e.g. severe emotional distress, intractable dispute)	Awareness of situations where referral is warranted (e.g. severe emotional distress, intractable dispute)	Understand situations where referral is warranted (e.g. severe emotional distress, intractable dispute)	Describe situations where referral is warranted (e.g. severe emotional distress, intractable dispute)	Determine situations where referral is warranted (e.g. severe emotional distress, intractable dispute)	Direct situations where referral is warranted (e.g. severe emotional distress, intractable dispute)
**6.3.1. Negotiate**	Apply the principles of negotiation and aim for a win-win outcome	Execute the principles of negotiation and aim for a win-win outcome	Understand the principles of negotiation and aim for a win-win outcome	Apply the principles of negotiation and aim for a win-win outcome	Demonstrate the principles of negotiation and aim for a win-win outcome	Implement the principles of negotiation and aim for a win-win outcome
	Manage and resolve relationship issues within and outside the team	Awareness of the need to manage and resolve relationship issues within and outside the team	Understand the need to manage and resolve relationship issues within and outside the team	Manage and resolve relationship issues within and outside the team	Demonstrating managing and resolving relationship issues within and outside the team	Establish a culture of managing and resolving relationship issues within and outside the team
	Model solution-focussed approaches to further the organisation's mission	Awareness of solution-focussed approaches to further the organisation's mission	Understand solution-focussed approaches to further the organisation's mission	Model solution-focussed approaches to further the organisation's mission	Determine solution-focussed approaches to further the organisation's mission	Develop solution-focussed approaches to further the organisation's mission
	Build consensus among parties	Awareness of the need to build consensus among parties	Understand how to build consensus among parties	Build consensus among parties	Demonstrate building consensus among parties	Establish a culture of building consensus among parties
	Present or propose alternative ways of doing things	Awareness of alternative ways of doing things	Compare alternative ways of doing things	Present or propose alternative ways of doing things	Evaluate alternative ways of doing things	Strategically analyse alternative ways of doing things
	Identify where fair approaches are being utilised to resolve issues	Awareness of where fair approaches are being utilised to resolve issues	Examine where fair approaches are being utilised to resolve issues	Identify where fair approaches are being utilised to resolve issues	Determine where fair approaches are being utilised to resolve issues	Strategically analyse where fair approaches are being utilised to resolve issues

COMPETENCY	BEHAVIOURAL COMPETENCIES (including knowledge requirements)	Associate	Practitioner	Specialist	Professional	Leader
**6.3.1. Negotiate (cont.)**	Seek to reach constructive solutions while maintaining positive working relationships	Awareness of the need to seek to reach constructive solutions while maintaining positive working relationships	Understand how to seek to reach constructive solutions while maintaining positive working relationships	Seek to reach constructive solutions while maintaining positive working relationships	Demonstrate seeking to reach constructive solutions while maintaining positive working relationships	Establish a culture of seeking to reach constructive solutions while maintaining positive working relationships
	Adapt style to take into account cultural differences regarding negotiation	Awareness of the need to adapt style to take into account cultural differences regarding negotiation	Understand how to adapt style to take into account cultural differences regarding negotiation	Adapt style to take into account cultural differences regarding negotiation	Demonstrate adapting style to take into account cultural differences regarding negotiation	Demonstrate adaptability of styles to take into account cultural differences regarding negotiation
	Build consensus at a high level for the benefit of all parties	Awareness of the need to build consensus at a high level for the benefit of all parties	Understand how to build consensus at a high level for the benefit of all parties	Build consensus at a high level for the benefit of all parties	Demonstrate building consensus at a high level for the benefit of all parties	Establish a culture of building consensus at a high level for the benefit of all parties
**6.3.2 Practice effective time management**	Set good timekeeping practice for the team	Awareness of the need for setting good timekeeping practice for the team	Understand how to set good timekeeping practice for the team	Set good timekeeping practice for the team	Demonstrate setting good timekeeping practice for the team	Implement setting good timekeeping practice for the team
	Set priorities, goals and workplans to achieve maximum effectiveness	Execute the practise of setting priorities, goals and workplans to achieve maximum effectiveness	Follow and enable the practise of setting priorities, goals and workplans to achieve maximum effectiveness	Set priorities, goals and workplans to achieve maximum effectiveness	Demonstrate setting priorities, goals and workplans to achieve maximum effectiveness	Implement and direct the process of setting priorities, goals and workplans to achieve maximum effectiveness
	Identify factors and/or criteria (e.g. urgency, importance, possibility of using alternative products or personnel) that impact the priority assigned to tasks	Awareness of factors and/or criteria (e.g. urgency, importance, possibility of using alternative products or personnel) that impact the priority assigned to tasks	Examine factors and/or criteria (e.g. urgency, importance, possibility of using alternative products or personnel) that impact the priority assigned to tasks	Identify factors and/or criteria (e.g. urgency, importance, possibility of using alternative products or personnel) that impact the priority assigned to tasks	Evaluate factors and/or criteria (e.g. urgency, importance, possibility of using alternative products or personnel) that impact the priority assigned to tasks	Strategically analyse factors and/or criteria (e.g. urgency, importance, possibility of using alternative products or personnel) that impact the priority assigned to tasks
	Monitor one's own progress against timekeeping objectives and targets	Awareness of the need to monitor one's own progress against timekeeping objectives and targets	Understand the process of monitoring one's own progress against timekeeping objectives and targets	Monitor one's own progress against timekeeping objectives and targets	Demonstrate monitoring one's own progress against timekeeping objectives and targets	Develop the methodology for monitoring one's own progress against timekeeping objectives and targets
	React quickly to change and reallocate time and resources accordingly	Awareness of the need to react quickly to change and reallocate time and resources accordingly	Understand the need to react quickly to change and reallocate time and resources accordingly	React quickly to change and reallocate time and resources accordingly	Demonstrate reacting quickly to change and reallocate time and resources accordingly	Establish a culture of reacting quickly to change and reallocate time and resources accordingly
	Strive to meet targets and deadlines	Awareness of the need to meet targets and deadlines	Understand the need to meet targets and deadlines	Strive to meet targets and deadlines	Demonstrate striving to meet targets and deadlines	Establish a culture of striving to meet targets and deadlines
	Make strategic decisions with regard to time and resources	Awareness of strategic decisions with regard to time and resources	Understand strategic decisions with regard to time and resources	Influence strategic decisions with regard to time and resources	Analyse strategic decisions with regard to time and resources	Implement strategic decisions with regard to time and resources
	Ensure ratio between staff time allocation and resources is appropriate	Awareness of the ratio between staff time allocation and resources that is appropriate	Understand the ratio between staff time allocation and resources that is appropriate	Ensure ratio between staff time allocation and resources is appropriate	Determine the ratio between staff time allocation and resources that is appropriate	Illustrate the ratio between staff time allocation and resources that is appropriate
	Use systems to organise and plan workload	Execute the use of systems to organise and plan workload	Enable the use of systems to organise and plan workload	Use systems to organise and plan workload	Demonstrate the use of systems to organise and plan workload	Implement the use of systems to organise and plan workload

COMPETENCY	BEHAVIOURAL COMPETENCIES (including knowledge requirements)	Associate	Practitioner	Specialist	Professional	Leader
6.3.2 Practice effective time management (cont.)	Establish priorities according to team and project goals	Execute priorities according to team and project goals	Examine priorities according to team and project goals	Establish priorities according to team and project goals	Determine priorities according to team and project goals	Direct priorities according to team and project goals
	Keep clear, detailed records of activities	Awareness of the need for keeping clear, detailed records of activities	Follow and enable the process of keeping clear, detailed records of activities	Keep clear, detailed records of activities	Demonstrate keeping clear, detailed records of activities	Implement and direct the process of keeping clear, detailed records of activities
	Manage interferences (e.g. telephones, interruptions) that consume time	Awareness of the need to manage interferences (e.g. telephones, interruptions) that consume time	Understand the need to manage interferences (e.g. telephones, interruptions) that consume time	Manage interferences (e.g. telephones, interruptions) that consume time	Determine how to manage interferences (e.g. telephones, interruptions) that consume time	Establish a culture of managing interferences (e.g, telephones, interruptions) that consume time
	Assist others in organising and managing their workloads	Awareness of the need to assist others in organising and managing their workloads	Understand the need to assist others in organising and managing their workloads	Assist others in organising and managing their workloads	Demonstrate assisting others in organising and managing their workloads	Establish a culture of assisting others in organising and managing their workloads
	Identify tasks or elements of tasks that may be delegated to other available personnel	Awareness of tasks or elements of tasks that may be delegated to other available personnel	Examine tasks or elements of tasks that may be delegated to other available personnel	Identify tasks or elements of tasks that may be delegated to other available personnel	Evaluate tasks or elements of tasks that may be delegated to other available personnel	Strategically analyse tasks or elements of tasks that may be delegated to other available personnel
	Describe the limitations applicable to delegation of specific tasks	Awareness of the limitations applicable to delegation of specific tasks	Understand the limitations applicable to delegation of specific tasks	Describe the limitations applicable to delegation of specific tasks	Determine the limitations applicable to delegation of specific tasks	Strategically analyse the limitations applicable to delegation of specific tasks
6.3.3 Take risk into account and implement security measures	Follow security guidelines, plans and standard operating procedures	Execute security guidelines, plans and standard operating procedures	Enable security guidelines, plans and standard operating procedures	Follow security guidelines, plans and standard operating procedures	Demonstrate security guidelines, plans and standard operating procedures	Implement security guidelines, plans and standard operating procedures
	Input into security planning and reviews	Awareness of the process of providing input into security planning and reviews	Understand the process of providing input into security planning and reviews	Provide input into security planning and reviews	Determine the process of providing input into security planning and reviews	Direct the process of providing input into security planning and reviews
	Ensure compliance with legal, regulatory, ethical and social requirements in humanitarian settings	Awareness of compliance with legal, regulatory, ethical and social requirements in humanitarian settings	Understand compliance with legal, regulatory, ethical and social requirements in humanitarian settings	Ensure compliance with legal, regulatory, ethical and social requirements in humanitarian settings	Ensure adherence with legal, regulatory, ethical and social compliance requirements in humanitarian settings	Take responsibility for compliance with legal, regulatory, ethical and social requirements in humanitarian settings
	Carry out responsibilities and follow instructions (e.g. completing a personal risk assessment and filling in travel plans)	Execute carrying out responsibilities and follow instructions (e.g. completing a personal risk assessment and filling in travel plans)	Enable carrying out responsibilities and follow instructions (e.g. completing a personal risk assessment and filling in travel plans)	Carry out responsibilities and follow instructions (e.g. completing a personal risk assessment and filling in travel plans)	Demonstrate carrying out responsibilities and following instructions (e.g. completing a personal risk assessment and filling in travel plans)	Establish a culture of carrying out responsibilities and following instructions (e.g. completing a personal risk assessment and filling in travel plans)
	Ensure personal behaviour does not impact personal or organisational security	Realise the need to ensure personal behaviour does not impact personal or organisational security	Understand the need to ensure personal behaviour does not impact personal or organisational security	Ensure personal behaviour does not impact personal or organisational security	Demonstrate ensuring personal behaviour does not impact personal or organisational security	Establish a culture of ensuring personal behaviour does not impact personal or organisational security

COMPETENCY	BEHAVIOURAL COMPETENCIES (including knowledge requirements)	Associate	Practitioner	Specialist	Professional	Leader
**6.4. Exhibit professional and ethical values**						
6.4.1 Demonstrate integrity	Accept responsibility for one's own work tasks and performance	Accept responsibility for one's own work tasks and performance	Accept responsibility for one's own work tasks and performance	Accept responsibility for one's own work tasks and performance	Demonstrate accepting responsibility for work tasks and performance	Establish a culture of accepting responsibility for work tasks and performance
	Work within a framework of clearly understood humanitarian values and ethics	Awareness of the need to work within a framework of clearly understood humanitarian values and ethics	Enable working within a framework of clearly understood humanitarian values and ethics	Work within a framework of clearly understood humanitarian values and ethics	Demonstrate working within a framework of clearly understood humanitarian values and ethics	Implement working within a framework of clearly understood humanitarian values and ethics
	Stand by decisions and hold others accountable when necessary	Stand by decisions and hold others accountable when necessary	Stand by decisions and hold others accountable when necessary	Stand by decisions and hold others accountable when necessary	Demonstrate standing by decisions and holding others accountable when necessary	Establish a culture of standing by decisions and holding others accountable when necessary
	Does not abuse one's own power or position	Does not abuse one's own power or position	Does not abuse one's own power or position	Does not abuse one's own power or position	Demonstrate not abusing one's own power or position	Establish a culture of not abusing one's own power or position
	Manage team members to ensure they do not abuse their power or position	Awareness of the process to manage team members to ensure they do not abuse their power or position	Enable the process of managing team members to ensure they do not abuse their power or position	Manage team members to ensure they do not abuse their power or position	Demonstrate the process of managing team members to ensure they do not abuse their power or position	Develop the process of managing team members to ensure they do not abuse their power or position
	Identify when individuals or the organisation is straying from organisation goals, and challenge them to uphold ethics	Realise when individuals or the organisation are straying from organisation goals, and challenge them to uphold ethics	Examine when individuals or the organisation are straying from organisation goals, and challenge them to uphold ethics	Identify when individuals or the organisation are straying from organisation goals, and challenge them to uphold ethics	Determine when individuals or the organisation are straying from organisation goals, and challenge them to uphold ethics	Establish a culture of identifying when individuals or the organisation are straying from organisation goals, and challenge them to uphold ethics
	Resist undue political pressure in decision making	Awareness of the need to resist undue political pressure in decision making	Understand the need to resist undue political pressure in decision making	Resist undue political pressure in decision making	Demonstrate resisting undue political pressure in decision making	Establish a culture of resisting undue political pressure in decision making
	Support staff in maintaining ethical stances	Awareness of the need to support staff in maintaining ethical stances	Understand the need to support staff in maintaining ethical stances	Support staff in maintaining ethical stances	Demonstrate supporting staff in maintaining ethical stances	Establish a culture of supporting staff in maintaining ethical stances
	Make time in team for ethical inquiry and reflection	Realise the need to make time in team for ethical inquiry and reflection	Enable the process of making time in team for ethical inquiry and reflection	Make time in team for ethical inquiry and reflection	Demonstrate making time in team for ethical inquiry and reflection	Implement making time in team for ethical inquiry and reflection
	Show consistency between expressed principles and behaviour	Awareness of the need to show consistency between expressed principles and behaviour	Understand the need to show consistency between expressed principles and behaviour	Show consistency between expressed principles and behaviour	Demonstrate showing consistency between expressed principles and behaviour	Establish a culture of showing consistency between expressed principles and behaviour
	Ensure that principles, values and ethics are embedded in policy	Awareness of principles, values and ethics that are embedded in policy	Understand that principles, values and ethics are embedded in policy	Provide input to ensure that principles, values and ethics are embedded in policy	Ensure that principles, values and ethics are embedded in policy	Develop policies where principles, values and ethics are embedded
	Act without consideration of personal gain	Realise the need to act without consideration of personal gain	Understand the need to act without consideration of personal gain	Act without consideration of personal gain	Demonstrate acting without consideration of personal gain	Establish a culture of acting without consideration of personal gain

COMPETENCY	BEHAVIOURAL COMPETENCIES (including knowledge requirements)	Associate	Practitioner	Specialist	Professional	Leader
6.4.1 Demonstrate integrity (cont.)	Promote transparency in decision making structures and processes	Awareness of the need for transparency in decision making structures and processes	Enable promoting transparency in decision making structures and processes	Promote transparency in decision making structures and processes	Demonstrate promoting transparency in decision making structures and processes	Establish a culture of promoting transparency in decision making structures and processes
	Ensure programmes are acting with integrity, and recognise the impact of not doing so	Awareness of the need to ensure programmes are acting with integrity, and recognising the impact of not doing so	Understand the need to ensure programmes are acting with integrity, and recognising the impact of not doing so	Provide input on ensuring programmes are acting with integrity, and recognising the impact of not doing so	Ensure programmes are acting with integrity, and recognise the impact of not doing so	Establish a culture of ensuring programmes are acting with integrity, and recognise the impact of not doing so
	Recognise one's own limitations and act upon them	Recognise one's own limitations and act upon them	Recognise one's own limitations and act upon them	Recognise one's own limitations and act upon them	Demonstrate recognising one's own limitations and act upon them	Establish a culture of recognising one's own limitations and act upon them
	Follow all standard operating procedures	Execute all standard operating procedures	Follow all standard operating procedures	Apply all standard operating procedures	Demonstrate all standard operating procedures	Develop all standard operating procedures
	Work in a safe and legal way	Execute the practise of working in a safe and legal way	Follow the practise of working in a safe and legal way	Work in a safe and legal way	Demonstrate working in a safe and legal way	Implement working in a safe and legal way
	Demonstrate respect, dignity and consideration for consumers	Demonstrate respect, dignity and consideration for consumers	Demonstrate respect, dignity and consideration for consumers	Demonstrate respect, dignity and consideration for consumers	Demonstrate respect, dignity and consideration for consumers	Demonstrate respect, dignity and consideration for consumers
	Discuss the impact of a no-blame culture on reporting and preventing recurrence of incidents	Awareness of the impact of a no-blame culture on reporting and preventing recurrence of incidents	Compare the impact of a no-blame culture on reporting and preventing recurrence of incidents	Discuss the impact of a no-blame culture on reporting and preventing recurrence of incidents	Determine the impact of a no-blame culture on reporting and preventing recurrence of incidents	Strategically analyse the impact of a no-blame culture on reporting and preventing recurrence of incidents
	Describe requests of colleagues that might be regarded as unreasonable	Awareness of requests of colleagues that might be regarded as unreasonable	Interpret requests of colleagues that might be regarded as unreasonable	Describe requests of colleagues that might be regarded as unreasonable	Evaluate requests of colleagues that might be regarded as unreasonable	Strategically analyse requests of colleagues that might be regarded as unreasonable
	Communicate (verbally and by example) expectations of the desired standards and approaches to be adopted	Awareness of the need to communicate (verbally and by example) expectations of the desired standards and approaches to be adopted	Understand the need to communicate (verbally and by example) expectations of the desired standards and approaches to be adopted	Communicate (verbally and by example) expectations of the desired standards and approaches to be adopted	Demonstrate communicating (verbally and by example) expectations of the desired standards and approaches to be adopted	Establish a culture of communicating (verbally and by example) expectations of the desired standards and approaches to be adopted
	Describe roles and responsibilities in terms of the position statement/duty statement of the position held	Awareness of roles and responsibilities in terms of the position statement/ duty statement of the position held	Compare roles and responsibilities in terms of the position statement/duty statement of the position held	Describe roles and responsibilities in terms of the position statement/duty statement of the position held	Determine roles and responsibilities in terms of the position statement/duty statement of the position held	Create roles and responsibilities in terms of the position statement/duty statement of the position held
	Demonstrate punctuality	Demonstrate punctuality	Demonstrate punctuality	Demonstrate punctuality	Demonstrate punctuality	Demonstrate punctuality
	Explain the obligation to apply professional care and expertise to deliver high quality of services	Awareness of the obligation to apply professional care and expertise to deliver high quality of services	Understand the obligation to apply professional care and expertise to deliver high quality of services	Explain the obligation to apply professional care and expertise to deliver high quality of services	Demonstrate applying professional care and expertise to deliver high quality of services	Establish the obligation to apply professional care and expertise to deliver high quality of services
	Demonstrate care and attention to detail in undertaking work activities	Demonstrate care and attention to detail in undertaking work activities	Demonstrate care and attention to detail in undertaking work activities	Demonstrate care and attention to detail in undertaking work activities	Demonstrate care and attention to detail in undertaking work activities	Establish a culture of demonstrating care and attention to detail in undertaking work activities
	Demonstrate appropriate attire and presentation for the role and situation	Demonstrate appropriate attire and presentation for the role and situation	Demonstrate appropriate attire and presentation for the role and situation	Describe appropriate attire and presentation for the role and situation	Determine appropriate attire and presentation for the role and situation	Direct appropriate attire and presentation for the role and situation

COMPETENCY	BEHAVIOURAL COMPETENCIES (including knowledge requirements)	Associate	Practitioner	Specialist	Professional	Leader
6.4.1 Demonstrate integrity (cont.)	Recognise and take responsibility for emotions	Recognise and take responsibility for emotions	Recognise and take responsibility for emotions	Recognise and take responsibility for emotions	Demonstrate recognising and taking responsibility for emotions	Establish a culture of recognising and taking responsibility for emotions
	Create and maintain an environment in which others can talk and act without fear of repercussion	Awareness of the need to an environment in which others can talk and act without fear of repercussion	Enable an environment in which others can talk and act without fear of repercussion	Describe an environment in which others can talk and act without fear of repercussion	Demonstrate an environment in which others can talk and act without fear of repercussion	Create and maintain an environment in which others can talk and act without fear of repercussion
	Seek to keep commitments and not let people down	Seek to keep commitments and not let people down	Seek to keep commitments and not let people down	Seek to keep commitments and not let people down	Demonstrate keeping commitments and not letting people down	Establish a culture of keeping commitments and not letting people down
	Be trusting and cooperative when working alongside others	Be trusting and cooperative when working alongside others	Be trusting and cooperative when working alongside others	Be trusting and cooperative when working alongside others	Demonstrate being trusting and cooperative when working alongside others	Establish a culture of being trusting and cooperative when working alongside others
	Take on various responsibilities within the department as the need arises	Take on various responsibilities within the department as the need arises	Take on various responsibilities within the department as the need arises	Take on various responsibilities within the department as the need arises	Demonstrate taking on various responsibilities within the department as the need arises	Demonstrate taking on various responsibilities within the department as the need arises
6.4.2 Engage in continuous professional development	Seek out opportunities to learn, and integrate new knowledge and skills into work	Seek out opportunities to learn, and integrate new knowledge and skills into work	Seek out opportunities to learn, and integrate new knowledge and skills into work	Seek out opportunities to learn, and integrate new knowledge and skills into work	Demonstrate seeking out opportunities to learn, and integrating new knowledge and skills into work	Establish a culture of seeking out opportunities to learn, and integrating new knowledge and skills into work
	Identify gaps in knowledge and skills for completing specific tasks, and develop a plan to acquire them	Awareness of gaps in knowledge and skills for completing specific tasks, and develop a plan to acquire them	Examine gaps in knowledge and skills for completing specific tasks, and develop a plan to acquire them	Identify gaps in knowledge and skills for completing specific tasks, and develop a plan to acquire them	Determine gaps in knowledge and skills for completing specific tasks, and develop a plan to acquire them	Strategically analyse gaps in knowledge and skills for completing specific tasks, and develop a plan to acquire them
	Listen to and invite feedback from others on one's own performance	Listen to and invite feedback from others on one's own performance	Listen to and invite feedback from others on one's own performance	Listen to and invite feedback from others on one's own performance	Demonstrate listening to and inviting feedback from others on one's own performance	Establish a culture of listening to and inviting feedback from others on one's own performance
	Use lifelong learning (continuous striving to gain knowledge and maintain competence) in the context of career development and the professional's role in delivering healthcare services	Use lifelong learning (continuous striving to gain knowledge and maintain competence) in the context of career development and the professional's role in delivering healthcare services	Use lifelong learning (continuous striving to gain knowledge and maintain competence) in the context of career development and the professional's role in delivering healthcare services	Use lifelong learning (continuous striving to gain knowledge and maintain competence) in the context of career development and the professional's role in delivering healthcare services	Demonstrate using lifelong learning (continuous striving to gain knowledge and maintain competence) in the context of career development and the professional's role in delivering healthcare services	Develop a methodology and instil a culture of using lifelong learning (continuous striving to gain knowledge and maintain competence) in the context of career development and the professional's role in delivering healthcare services
	Create organisational systems for capturing learning and ensuring lessons learned	Awareness of organisational systems for capturing learning and ensuring lessons learned	Enable organisational systems for capturing learning and ensuring lessons learned	Use organisational systems for capturing learning and ensuring lessons learned	Analyse organisational systems for capturing learning and ensuring lessons learned	Create organisational systems for capturing learning and ensuring lessons learned
	Employ reflective learning within the team, in which lessons are captured and integrated into future projects	Awareness of the need for reflective learning within the team, in which lessons are captured and integrated into future projects	Enable reflective learning within the team, in which lessons are captured and integrated into future projects	Employ reflective learning within the team, in which lessons are captured and integrated into future projects	Employ reflective learning within the team, in which lessons are captured and integrated into future projects	Employ reflective learning within the team, in which lessons are captured and integrated into future projects

COMPETENCY	BEHAVIOURAL COMPETENCIES (including knowledge requirements)	Associate	Practitioner	Specialist	Professional	Leader
6.4.2 Engage in continuous professional development (cont.)	Promote continuous learning as an integral part of organisational performance	Awareness that continuous learning is an integral part of organisational performance	Enable continuous learning as an integral part of organisational performance	Use continuous learning as an integral part of organisational performance	Promote continuous learning as an integral part of organisational performance	Promote continuous learning as an integral part of organisational performance
	Be open to new ideas and different perspectives	Be open to new ideas and different perspectives	Be open to new ideas and different perspectives	Be open to new ideas and different perspectives	Demonstrate being open to new ideas and different perspectives	Establish a culture of being open to new ideas and different perspectives
	Keep up to date in the place of work with input from supervisors	Keep up to date in the place of work with input from supervisors	Keep up to date in the place of work with input from supervisors	Keep up to date in the place of work with input from supervisors	Demonstrate keeping up to date in the place of work with input from supervisors	Demonstrate keeping up to date in the place of work with input from supervisors
	Document continual professional development activities	Awareness of the need to document continual professional development activities	Follow and enable the practise of documenting continual professional development activities	Document continual professional development activities	Demonstrate documenting continual professional development activities	Implement and direct the practise of documenting continual professional development activities
	Use multiple techniques to acquire new knowledge and skills; process and retain information; and identify when it is necessary to acquire new knowledge and skills.	Awareness of multiple techniques to acquire new knowledge and skills; process and retain information; and identify when it is necessary to acquire new knowledge and skills.	Enable the use of multiple techniques to acquire new knowledge and skills; process and retain information; and identify when it is necessary to acquire new knowledge and skills.	Use multiple techniques to acquire new knowledge and skills; process and retain information; and identify when it is necessary to acquire new knowledge and skills.	Demonstrate the use of multiple techniques to acquire new knowledge and skills; process and retain information; and identify when it is necessary to acquire new knowledge and skills.	Implement the use of multiple techniques to acquire new knowledge and skills; process and retain information; and identify when it is necessary to acquire new knowledge and skills.
	Participate in professional organisations and/or committees	Awareness of professional organisations and/or committees	Attend professional organisations and/or committees	Participate in professional organisations and/or committees	Convene professional organisations and/or committees	Direct professional organisations and/or committees
	Describe and/or demonstrate quality improvement and/or quality assurance activities participated in	Observe and/or execute quality improvement and/or quality assurance activities participated in	Understand and/or enable quality improvement and/or quality assurance activities participated in	Describe and/or apply quality improvement and/or quality assurance activities participated in	Determine and/or demonstrate quality improvement and/or quality assurance activities participated in	Develop and/or illustrate quality improvement and/or quality assurance activities participated in
	Reflect on performance and seek what needs improvement	Reflect on performance and seek what needs improvement	Reflect on performance and seek what needs improvement	Reflect on performance and seek what needs improvement	Demonstrate reflecting on performance and seeking what needs improvement	Establish a culture of reflecting on performance and seeking what needs improvement
	Engage in personal career development, identifying occupational interests, strengths, options and opportunities	Awareness of personal career development, identifying occupational interests, strengths, options and opportunities	Enable the process of engaging in personal career development, identifying occupational interests, strengths, options and opportunities	Engage in personal career development, identifying occupational interests, strengths, options and opportunities	Demonstrate the process of engaging in personal career development, identifying occupational interests, strengths, options and opportunities	Develop the methodology for personal career development, identifying occupational interests, strengths, options and opportunities
	Contribute to others' professional development	Awareness of the need to contribute to others' professional development	Understand the need to contribute to others' professional development	Contribute to others' professional development	Demonstrate contributing to others' professional development	Establish a culture of contributing to others' professional development
	Share experiences and learning internally and externally	Share experiences and learning internally and externally	Enable sharing experiences and learning internally and externally	Share experiences and learning internally and externally	Demonstrate sharing experiences and learning internally and externally	Demonstrate sharing experiences and learning internally and externally
	Seek out challenging projects outside of core experience and achieve solid results	Awareness of the need to seek out challenging projects outside of core experience and achieve solid results	Understand the need to seek out challenging projects outside of core experience and achieve solid results	Seek out challenging projects outside of core experience and achieve solid results	Demonstrate seeking out challenging projects outside of core experience and achieve solid results	Establish a culture of seeking out challenging projects outside of core experience and achieve solid results

COMPETENCY	BEHAVIOURAL COMPETENCIES (including knowledge requirements)	Associate	Practitioner	Specialist	Professional	Leader
6.5 Prove leadership abilities	Input to meetings and programme development	Awareness of the need to provide input to meetings and programme development	Understand how to provide input to meetings and programme development	Provide input to meetings and programme development	Analyse input to meetings and programme development	Strategically analyse input to meetings and programme development
	Look for future trends and issues, and assist the organisation in meeting the challenges	Awareness of future trends and issues, and assist the organisation in meeting the challenges	Examine future trends and issues, and assist the organisation in meeting the challenges	Identify future trends and issues, and assist the organisation in meeting the challenges	Look for future trends and issues, and assist the organisation in meeting the challenges	Analyse future trends and issues, and assist the organisation in meeting the challenges
	Contribute to a collaborative working environment	Contribute to a collaborative working environment	Enable contributing to a collaborative working environment	Contribute to a collaborative working environment	Contribute to a collaborative working environment	Develop a process to contribute to a collaborative working environment
	Drive for change and improvement develops opportunities for the organisation and sector	Awareness that change and improvement develops opportunities for the organisation and sector	Understand that change and improvement develops opportunities for the organisation and sector	Describe how change and improvement develops opportunities for the organisation and sector	Demonstrate a drive for change and improvement develops opportunities for the organisation and sector	Demonstrate a drive for change and improvement develops opportunities for the organisation and sector
	Channel energy and ideas toward resolving issues	Channel energy and ideas toward resolving issues	Channel energy and ideas toward resolving issues	Channel energy and ideas toward resolving issues	Demonstrate channelling energy and ideas toward resolving issues	Demonstrate channelling energy and ideas toward resolving issues
	Communicate with influence	Awareness of the need to communicate with influence	Enable communicating with influence	Communicate with influence	Demonstrate the practise of communicating with influence	Demonstrate the practise of communicating with influence
	Show courage to take an unpopular stance when needed	Awareness of the need to show courage to take an unpopular stance when needed	Understand the need to show courage to take an unpopular stance when needed	Value the practise of showing courage to take an unpopular stance when needed	Demonstrate showing courage to take an unpopular stance when needed	Demonstrate showing courage to take an unpopular stance when needed
	Steer and implement change organisationally	Awareness of the need to steer and implement change organisationally	Understand the need to steer and implement change organisationally	Steer and implement change organisationally	Demonstrate steering and implementing change organisationally	Demonstrate steering and implementing change organisationally
	Anticipate and resolve conflict	Awareness of the need to anticipate and resolve conflict	Understand the need to anticipate and resolve conflict	Anticipate and resolve conflict	Demonstrate anticipating and resolving conflict	Establish the culture of anticipating and resolving conflict
	Show initiative in working methods	Show initiative in working methods	Show initiative in working methods	Show initiative in working methods	Demonstrate showing initiative in working methods	Establish a culture of showing initiative in working methods
	Exemplify personal drive and integrity	Exemplify personal drive and integrity	Exemplify personal drive and integrity	Exemplify personal drive and integrity	Exemplify personal drive and integrity	Exemplify personal drive and integrity
	Serve as a role model for others to follow	Serve as a role model for others to follow	Serve as a role model for others to follow	Serve as a role model for others to follow	Demonstrate serving as a role model for others to follow	Demonstrate serving as a role model for others to follow
	Demonstrate leadership and practice management skills, initiative and efficiency	Awareness of the need to demonstrate leadership and practice management skills, initiative and efficiency	Understand the need to demonstrate leadership and practice management skills, initiative and efficiency	Demonstrate leadership and practice management skills, initiative and efficiency	Demonstrate leadership and practice management skills, initiative and efficiency	Demonstrate leadership and practice management skills, initiative and efficiency
	Inspire others through professional excellence, innovation and communication	Awareness of the need to inspire others through professional excellence, innovation and communication	Understand the need to inspire others through professional excellence, innovation and communication	Inspire others through professional excellence, innovation and communication	Demonstrate inspiring others through professional excellence, innovation and communication	Demonstrate inspiring others through professional excellence, innovation and communication

COMPETENCY	BEHAVIOURAL COMPETENCIES (including knowledge requirements)	Associate	Practitioner	Specialist	Professional	Leader
**6.5 Prove leadership abilities (cont.)**	Discuss situations in which a change in leadership style would be warranted (e.g. directive versus consultative style in the event of a crisis)	Awareness of situations in which a change in leadership style would be warranted (e.g. directive versus consultative style in the event of a crisis)	Understand situations in which a change in leadership style would be warranted (e.g. directive versus consultative style in the event of a crisis)	Discuss situations in which a change in leadership style would be warranted (e.g. directive versus consultative style in the event of a crisis)	Determine situations in which a change in leadership style would be warranted (e.g. directive versus consultative style in the event of a crisis)	Illustrate situations in which a change in leadership style would be warranted (e.g. directive versus consultative style in the event of a crisis)
**6.5.1 Demonstrate resilience and ability to manage stress**	Identify symptoms of stress and take steps to reduce stress	Awareness of symptoms of stress and take steps to reduce stress	Examine symptoms of stress and take steps to reduce stress	Identify symptoms of stress and take steps to reduce stress	Demonstrate identifying symptoms of stress and take steps to reduce stress	Demonstrate identifying symptoms of stress and take steps to reduce stress
	See the bigger picture and help others to	See the bigger picture and help others to	See the bigger picture and help others to	See the bigger picture and help others to	Demonstrate seeing the bigger picture and help others to	Demonstrate seeing the bigger picture and help others to
	Recover quickly from setbacks	Recover quickly from setbacks	Recover quickly from setbacks	Recover quickly from setbacks	Demonstrate recovering quickly from setbacks	Demonstrate recovering quickly from setbacks
	Draw on previous experience and support mechanisms to reduce the impact of stress on self and others	Draw on previous experience and support mechanisms to reduce the impact of stress on self and others	Draw on previous experience and support mechanisms to reduce the impact of stress on self and others	Draw on previous experience and support mechanisms to reduce the impact of stress on self and others	Demonstrate drawing on previous experience and support mechanisms to reduce the impact of stress on self and others	Demonstrate drawing on previous experience and support mechanisms to reduce the impact of stress on self and others
	Create a working environment that aims to minimise pressure and stress	Awareness of the need for a working environment that aims to minimise pressure and stress	Enable a working environment that aims to minimise pressure and stress	Describe a working environment that aims to minimise pressure and stress	Determine a working environment that aims to minimise pressure and stress	Create a working environment that aims to minimise pressure and stress
	Cope well under pressure, particularly in difficult environments	Cope well under pressure, particularly in difficult environments	Cope well under pressure, particularly in difficult environments	Cope well under pressure, particularly in difficult environments	Demonstrate coping well under pressure, particularly in difficult environments	Demonstrate coping well under pressure, particularly in difficult environments
	Act as a role model for others and display courage under difficult circumstances	Act as a role model for others and display courage under difficult circumstances	Act as a role model for others and display courage under difficult circumstances	Act as a role model for others and display courage under difficult circumstances	Demonstrate acting as a role model for others and display courage under difficult circumstances	Demonstrate acting as a role model for others and display courage under difficult circumstances
	Recognise the limitations of staff and take action to limit their exposure to harm when needed	Awareness of the need to recognise the limitations of staff and taking action to limit their exposure to harm when needed	Understand the need to recognise the limitations of staff and taking action to limit their exposure to harm when needed	Recognise the limitations of staff and take action to limit their exposure to harm when needed	Demonstrate recognising the limitations of staff and taking action to limit their exposure to harm when needed	Develop a methodology for recognising the limitations of staff and taking action to limit their exposure to harm when needed
	Identify and make use of personal support mechanisms	Awareness of personal support mechanisms	Enable the use of personal support mechanisms	Identify and make use of personal support mechanisms	Demonstrate making use of personal support mechanisms	Implement the use of personal support mechanisms
	Help others identify personal support mechanisms	Help others identify personal support mechanisms	Help others identify personal support mechanisms	Help others identify personal support mechanisms	Demonstrate helping others identify personal support mechanisms	Demonstrate helping others identify personal support mechanisms
	Influence organisational policy to support self-care in agencies	Observe organisational policy to support self-care in agencies	Follow organisational policy to support self-care in agencies	Influence organisational policy to support self-care in agencies	Determine organisational policy to support self-care in agencies	Direct organisational policy to support self-care in agencies

COMPETENCY	BEHAVIOURAL COMPETENCIES (including knowledge requirements)	Associate	Practitioner	Specialist	Professional	Leader
**6.6 Abide by rules / laws / legislation**	List and comply with the legislation that covers the practice of public health supply chains and healthcare, and describe its purpose	Awareness of the legislation that covers the practice of public health supply chains and healthcare, and describe its purpose	Understand the legislation that covers the practice of public health supply chains and healthcare, and describe its purpose	List and comply with the legislation that covers the practice of public health supply chains and healthcare, and describe its purpose	Demonstrate compliance with the legislation that covers the practice of public health supply chains and healthcare, and describe its purpose	Ensure compliance with the legislation that covers the practice of public health supply chains and healthcare, and describe its purpose
	Describe requirements of professional codes, guidelines and standards adopted as part of the legislative framework	Awareness of requirements of professional codes, guidelines and standards adopted as part of the legislative framework	Understand requirements of professional codes, guidelines and standards adopted as part of the legislative framework	Describe requirements of professional codes, guidelines and standards adopted as part of the legislative framework	Analyse requirements of professional codes, guidelines and standards adopted as part of the legislative framework	Implement requirements of professional codes, guidelines and standards adopted as part of the legislative framework
	Keep up to date with changes in legal instruments, as informed by national-level managers	Awareness of the need to keep up to date with changes in legal instruments, as informed by national-level managers	Understand the need to keep up to date with changes in legal instruments, as informed by national-level managers	Keep up to date with changes in legal instruments, as informed by national-level managers	Demonstrate keeping up to date with changes in legal instruments, as informed by national-level managers	Develop methodology to keep up to date with changes in legal instruments, as informed by national-level managers
	Comply with international regulations in decision making for the distribution system, including customs regulations on import and export requirements (e.g. trade tariffs and duties on imported goods) and security regulations (e.g. 2007 SAFE Ports Act, NAFTA or European Union trade agreements)	Execute the practise of compliance with international regulations in decision making for the distribution system, including customs regulations on import and export requirements (e.g. trade tariffs and duties on imported goods) and security regulations (e.g. 2007 SAFE Ports Act, NAFTA or European Union trade agreements)	Enable the practise of compliance with international regulations in decision making for the distribution system, including customs regulations on import and export requirements (e.g. trade tariffs and duties on imported goods) and security regulations (e.g. 2007 SAFE Ports Act, NAFTA or European Union trade agreements)	Comply with international regulations in decision making for the distribution system, including customs regulations on import and export requirements (e.g. trade tariffs and duties on imported goods) and security regulations (e.g. 2007 SAFE Ports Act, NAFTA or European Union trade agreements)	Demonstrate compliance with international regulations in decision making for the distribution system, including customs regulations on import and export requirements (e.g. trade tariffs and duties on imported goods) and security regulations (e.g. 2007 SAFE Ports Act, NAFTA or European Union trade agreements)	Ensure compliance with international regulations in decision making for the distribution system, including customs regulations on import and export requirements (e.g. trade tariffs and duties on imported goods) and security regulations (e.g. 2007 SAFE Ports Act, NAFTA or European Union trade agreements)
	Apply and understand regulatory affairs and the key aspects of pharmaceutical registration and legislation	Apply and understand regulatory affairs and the key aspects of pharmaceutical registration and legislation	Apply and understand regulatory affairs and the key aspects of pharmaceutical registration and legislation	Apply and understand regulatory affairs and the key aspects of pharmaceutical registration and legislation	Demonstrate applying and understanding regulatory affairs and the key aspects of pharmaceutical registration and legislation	Demonstrate in-depth knowledge of regulatory affairs and the key aspects of pharmaceutical registration and legislation
	Apply knowledge of the principles of business economics and intellectual property rights, including the basics of patent interpretation	Awareness of the principles of business economics and intellectual property rights, including the basics of patent interpretation	Understand the principles of business economics and intellectual property rights, including the basics of patent interpretation	Apply knowledge of the principles of business economics and intellectual property rights, including the basics of patent interpretation	Demonstrate knowledge of the principles of business economics and intellectual property rights, including the basics of patent interpretation	Demonstrate in-depth knowledge of the principles of business economics and intellectual property rights, including patent interpretation
	Knowledge of applicable procurement law and the practical effects of a contract's terms and conditions	Basic operational knowledge of applicable procurement law and the practical effects of a contract's terms and conditions	Show knowledge of applicable procurement law and the practical effects of a contract's terms and conditions	Apply knowledge of applicable procurement law and the practical effects of a contract's terms and conditions	Demonstrate knowledge of applicable procurement law and the practical effects of a contract's terms and conditions	Demonstrate in-depth knowledge of applicable procurement law and the practical effects of a contract's terms and conditions
	Understand current industry and government regulations governing sustainability	Awareness of current industry and government regulations governing sustainability	Understand current industry and government regulations governing sustainability	Describe current industry and government regulations governing sustainability	Evaluate current industry and government regulations governing sustainability	Strategically analyse current industry and government regulations governing sustainability

COMPETENCY	BEHAVIOURAL COMPETENCIES (including knowledge requirements)	Associate	Practitioner	Specialist	Professional	Leader
6.6 Abide by rules / laws / legislation (cont.)	Working knowledge of key hazardous materials handling laws	Execute a working knowledge of key hazardous materials handling laws	Show a working knowledge of key hazardous materials handling laws	Apply a working knowledge of key hazardous materials handling laws	Demonstrate a working knowledge of key hazardous materials handling laws	Demonstrate in-depth knowledge of key hazardous materials handling laws
	Implement policies and procedures relating to workplace safety that are consistent with agreed or recognised standards	Execute policies and procedures relating to workplace safety that are consistent with agreed or recognised standards	Enable policies and procedures relating to workplace safety that are consistent with agreed or recognised standards	Apply policies and procedures relating to workplace safety that are consistent with agreed or recognised standards	Determine policies and procedures relating to workplace safety that are consistent with agreed or recognised standards	Implement policies and procedures relating to workplace safety that are consistent with agreed or recognised standards
	Describe professional standards and conventions, as well as workplace policies and procedures, for preparing pharmaceutical products	Execute professional standards and conventions, as well as workplace policies and procedures, for preparing pharmaceutical products	Enable professional standards and conventions, as well as workplace policies and procedures, for preparing pharmaceutical products	Describe professional standards and conventions, as well as workplace policies and procedures, for preparing pharmaceutical products	Analyse professional standards and conventions, as well as workplace policies and procedures, for preparing pharmaceutical products	Implement professional standards and conventions, as well as workplace policies and procedures, for preparing pharmaceutical products
	Follow all standard operating procedures	Execute all standard operating procedures	Follow all standard operating procedures	Apply all standard operating procedures	Demonstrate all standard operating procedures	Develop all standard operating procedures

7. Technology

COMPETENCY	BEHAVIOURAL COMPETENCIES	Associate	Practitioner	Specialist	Professional	Leader
7.1 Data Science	Describe the end to end MIS technology components that are inherent in advanced MIS systems and explore how these may be relevant in different supply chain contexts	Awareness of end to end MIS technology components that are inherent in advanced MIS systems and explore how these may be relevant in different supply chain contexts	Understand end to end MIS technology components that are inherent in advanced MIS systems and explore how these may be relevant in different supply chain contexts	Describe the end to end MIS technology components that are inherent in advanced MIS systems and explore how these may be relevant in different supply chain contexts	Analyse end to end MIS technology components that are inherent in advanced MIS systems and explore how these may be relevant in different supply chain contexts	Strategically analyse end to end MIS technology components that are inherent in advanced MIS systems and explore how these may be relevant in different supply chain contexts
	Identify emerging technological approaches to data visualization and analysis	Awareness of emerging technological approaches to data visualisation and analysis	Compare emerging technological approaches to data visualization and analysis	Identify emerging technological approaches to data visualization and analysis	Determine emerging technological approaches to data visualization and analysis	Implement emerging technological approaches to data visualisation and analysis
	Awareness of analytics (data interpretation and decision making) and understand how data science can be applied within supply chain	Awareness of analytics (data interpretation and decision making)	Awareness of analytics (data interpretation and decision making)	Identify how data science can be applied within supply chain	Analyse how data science can be applied within supply chain	Strategically analyse how data science can be applied within supply chain
	Ability to interact, provide guidance for data (dashboard) consolidation in various levels and use the tech tools	Ability to interact, provide guidance for data (dashboard) consolidation in various levels and use the tech tools	Enable the ability to interact, provide guidance for data (dashboard) consolidation in various levels and use the tech tools	Ability to interact, provide guidance for data (dashboard) consolidation in various levels and use the tech tools	Demonstrate the ability to interact, provide guidance for data (dashboard) consolidation in various levels and use the tech tools	Demonstrate the ability to interact, provide guidance for data (dashboard) consolidation in various levels and use the tech tools
	Support movement of data into electronic form at lowest/remote SC level	Support movement of data into electronic form at lowest/remote SC level	Support movement of data into electronic form at lowest/remote SC level	Support movement of data into electronic form at lowest/remote SC level	Support movement of data into electronic form at lowest/remote SC level	Support movement of data into electronic form at lowest/remote SC level
7.2 Blockchain	Evaluate blockchain as a value-add capability for relevant global health use cases	Awareness of blockchain as a value-add capability for relevant global health use cases	Examine blockchain as a value-add capability for relevant global health use cases	Describe blockchain as a value-add capability for relevant global health use cases	Evaluate blockchain as a value-add capability for relevant global health use cases	Strategically analyse blockchain as a value-add capability for relevant global health use cases
7.3 UAVs	Describe the current UAV landscape, their application and the enabling environment required to support country implementation	Awareness of the current UAV landscape, their application and the enabling environment required to support country implementation	Understand the current UAV landscape, their application and the enabling environment required to support country implementation	Describe the current UAV landscape, their application and the enabling environment required to support country implementation	Analyse the current UAV landscape, their application and the enabling environment required to support country implementation	Demonstrate in-depth knowledge of the current UAV landscape, their application and the enabling environment required to support country implementation
	Evaluate UAVs as a value-add capability for relevant global health use cases	Awareness of UAVs as a value-add capability for relevant global health use cases	Understand UAVs as a value-add capability for relevant global health use cases	Describe UAVs as a value-add capability for relevant global health use cases	Evaluate UAVs as a value-add capability for relevant global health use cases	Strategically analyse UAVs as a value-add capability for relevant global health use cases

COMPETENCY	BEHAVIOURAL COMPETENCIES	Associate	Practitioner	Specialist	Professional	Leader
**7.4 Temperature and monitoring**	Describe the current technology approaches for Temperature Monitoring & Sensors	Awareness of the current technology approaches for Temperature Monitoring & Sensors	Compare the current technology approaches for Temperature Monitoring & Sensors	Describe the current technology approaches for Temperature Monitoring & Sensors	Analyse the current technology approaches for Temperature Monitoring & Sensors	Strategically analyse the current technology approaches for Temperature Monitoring & Sensors
	Consider what is required to implement this technology in a country context	Awareness of what is required to implement this technology in a country context	Consider what is required to implement this technology in a country context	Describe what is required to implement this technology in a country context	Evaluate what is required to implement this technology in a country context	Strategically analyse what is required to implement this technology in a country context
**7.5 Planning systems**	Describe blockchain and understand the building blocks for planning systems	Awareness of blockchain and understand the building blocks for planning systems	Examine blockchain and understand the building blocks for planning systems	Describe blockchain and understand the building blocks for planning systems	Analyse blockchain and understand the building blocks for planning systems	Implement blockchain and understand the building blocks for planning systems
	Evaluate planning systems as a value-add capability for relevant global health use cases	Awareness of planning systems as a value-add capability for relevant global health use cases	Examine planning systems as a value-add capability for relevant global health use cases	Describe planning systems as a value-add capability for relevant global health use cases	Evaluate planning systems as a value-add capability for relevant global health use cases	Analyse planning systems as a value-add capability for relevant global health use cases
**7.6 ERP includes function of LMIS**	Support a Master Data Management System	Awareness of a Master Data Management System	Enable a Master Data Management System	Support a Master Data Management System	Evaluate a Master Data Management System	Implement a Master Data Management System
	Understand the critical components of an ERP system	Awareness of the critical components of an ERP system	Understand the critical components of an ERP system	Describe the critical components of an ERP system	Demonstrate an understanding of the critical components of an ERP system	Demonstrate an in-depth understanding of the critical components of an ERP system
	Understand the meaning of common document types found within an ERP/ LMIS, transport management systems, warehouse management systems, inventory management systems	Awareness of the meaning of common document types found within an ERP/LMIS	Understand the meaning of common document types found within an ERP/LMIS	Describe the meaning of common document types found within an ERP/LMIS	Demonstrate an understanding of the meaning of common document types found within an ERP/LMIS	Demonstrate an in-depth understanding of the meaning of common document types found within an ERP/LMIS
	Understand the logic of data visibility within an ERP	Awareness of the logic of data visibility within an ERP	Understand the logic of data visibility within an ERP	Describe the logic of data visibility within an ERP	Demonstrate an understanding of the logic of data visibility within an ERP	Demonstrate an in-depth understanding of the logic of data visibility within an ERP
**7.7 Automation**	Evaluate automation as a value-add capability for relevant global health use cases	Awareness of automation as a value-add capability for relevant global health use cases	Understand automation as a value-add capability for relevant global health use cases	Describe automation as a value-add capability for relevant global health use cases	Evaluate automation as a value-add capability for relevant global health use cases	Strategically analyse automation as a value-add capability for relevant global health use cases
**7.8 Artificial Intelligence**	Evaluate artificial intelligence as a value-add capability for relevant global health use cases	Awareness of artificial intelligence as a value-add capability for relevant global health use cases	Understand artificial intelligence as a value-add capability for relevant global health use cases	Describe artificial intelligence as a value-add capability for relevant global health use cases	Evaluate artificial intelligence as a value-add capability for relevant global health use cases	Strategically analyse artificial intelligence as a value-add capability for relevant global health use cases

COMPETENCY	BEHAVIOURAL COMPETENCIES	Associate	Practitioner	Specialist	Professional	Leader
**7.9 Additive Manufacturing**	Evaluate additive manufacturing as a value-add capability for relevant global health use cases	Awareness of additive manufacturing as a value-add capability for relevant global health use cases	Understand additive manufacturing as a value-add capability for relevant global health use cases	Describe additive manufacturing as a value-add capability for relevant global health use cases	Evaluate additive manufacturing as a value-add capability for relevant global health use cases	Strategically analyse additive manufacturing as a value-add capability for relevant global health use cases
**7.10 Internet of things**	Understand the opportunity of IoT used in conjunction with AI	Understand the opportunity of IoT used in conjunction with AI	Understand the opportunity of IoT used in conjunction with AI	Understand the opportunity of IoT used in conjunction with AI	Evaluate the opportunity of IoT used in conjunction with AI	Strategically analyse the opportunity of IoT used in conjunction with AI
	Evaluate Internet of things as a value-add capability for relevant global health use cases	Awareness of the internet of things as a value-add capability for relevant global health use cases	Understand the internet of things as a value-add capability for relevant global health use cases	Describe the internet of things as a value-add capability for relevant global health use cases	Evaluate the internet of things as a value-add capability for relevant global health use cases	Strategically analyse the internet of things as a value-add capability for relevant global health use cases
**7.11 Cloud Computing**	Evaluate cloud computing as a value-add capability for relevant global health use cases	Awareness of cloud computing as a value-add capability for relevant global health use cases	Understand cloud computing as a value-add capability for relevant global health use cases	Describe cloud computing as a value-add capability for relevant global health use cases	Evaluate cloud computing as a value-add capability for relevant global health use cases	Strategically analyse cloud computing as a value-add capability for relevant global health use cases
**7.12 Basic Office Skills**	Have a good understanding of common presentation authoring packages	Have a good understanding of common presentation authoring packages	Have a good understanding of common presentation authoring packages	Have a good understanding of common presentation authoring packages	Demonstrate a good understanding of common presentation authoring packages	Demonstrate a good understanding of common presentation authoring packages
	Have a good understanding of common spreadsheet authoring packages	Have a good understanding of common spreadsheet authoring packages	Have a good understanding of common spreadsheet authoring packages	Have a good understanding of common spreadsheet authoring packages	Demonstrate a good understanding of common spreadsheet authoring packages	Demonstrate a good understanding of common spreadsheet authoring packages
	Have a good understanding of common document authoring packages	Have a good understanding of common document authoring packages	Have a good understanding of common document authoring packages	Have a good understanding of common document authoring packages	Demonstrate a good understanding of common document authoring packages	Demonstrate a good understanding of common document authoring packages
	Have a good understanding of common email authoring packages	Have a good understanding of common email authoring packages	Have a good understanding of common email authoring packages	Have a good understanding of common email authoring packages	Demonstrate a good understanding of common email authoring packages	Demonstrate a good understanding of common email authoring packages
	Have a good understanding of common instant messaging packages	Have a good understanding of common instant messaging packages	Have a good understanding of common instant messaging packages	Have a good understanding of common instant messaging packages	Demonstrate a good understanding of common instant messaging packages	Demonstrate a good understanding of common instant messaging packages
**7.13 Have a command of technology**	Demonstrate the use of technology suitable to the job held	Execute the use of technology suitable to the job held	Enable the use of technology suitable to the job held	Apply the use of technology suitable to the job held	Demonstrate the use of technology suitable to the job held	Implement the use of technology suitable to the job held
	Monitor new developments and technologies in the sector	Awareness of the need to monitor new developments and technologies in the sector	Understand the need of monitoring new developments and technologies in the sector	Provide input on monitoring new developments and technologies in the sector	Monitor new developments and technologies in the sector	Review new developments and technologies in the sector
	Experiment with new technologies and recognise potential benefits for the sector	Awareness of new technologies and recognising potential benefits for the sector	Compare new technologies and recognising potential benefits for the sector	Identify new technologies and recognising potential benefits for the sector	Experiment with new technologies and recognising potential benefits for the sector	Analyse new technologies and recognise potential benefits for the sector

COMPETENCY	BEHAVIOURAL COMPETENCIES	Associate	Practitioner	Specialist	Professional	Leader
7.13 Have a command of technology	Ensure that resources and support are provided across the organisation to enable colleagues to make the best use of available technology	Awareness that resources and support are provided across the organisation to enable colleagues to make the best use of available technology	Understand that resources and support are provided across the organisation to enable colleagues to make the best use of available technology	Identify resources and support that are provided across the organisation to enable colleagues to make the best use of available technology	Ensure that resources and support are provided across the organisation to enable colleagues to make the best use of available technology	Analyse the resources and support that are provided across the organisation to enable colleagues to make the best use of available technology
	Ensure that the organisation has a strategy for technology use	Awareness of the fact that the organisation has a strategy for technology use	Understand that the organisation has a strategy for technology use	Ensure that the organisation has a strategy for technology use	Determine what the organisations' strategy is for technology use	Develop the organisations' strategy for technology use
	Describe how to minimise environmental damage through technology use	Awareness of how to minimise environmental damage through technology use	Compare how to minimise environmental damage through technology use	Describe how to minimise environmental damage through technology use	Determine how to minimise environmental damage through technology use	Strategically analyse how to minimise environmental damage through technology use
	Use technology to maximise effectiveness and efficiency	Basic operational knowledge of technology to maximise effectiveness and efficiency	Enable the use of technology to maximise effectiveness and efficiency	Use technology to maximise effectiveness and efficiency	Analyse the use of technology to maximise effectiveness and efficiency	Implement the use of technology to maximise effectiveness and efficiency
	Use field-based technology (e.g. radio, general packet radio service (GPRS), satellite phone)	Basic operational knowledge of field-based technology (e.g. radio, general packet radio service (GPRS), satellite phone)	Enable the use of field-based technology (e.g. radio, general packet radio service (GPRS), satellite phone)	Use field-based technology (e.g. radio, general packet radio service (GPRS), satellite phone)	Analyse the use of field-based technology (e.g. radio, general packet radio service (GPRS), satellite phone)	Implement the use of field-based technology (e.g. radio, general packet radio service (GPRS), satellite phone)
7.14 eProcurement	Understand how to transact with an eProcurement system where necessary	Awareness of how to transact with an eProcurement system where necessary	Understand how to transact with an eProcurement system where necessary	Describe how to transact with an eProcurement system where necessary	Determine how to transact with an eProcurement system where necessary	Develop a methodology on how to transact with an eProcurement system where necessary
	Evaluate eProcurement as a value-add capability for relevant global health use cases	Awareness of eProcurement as a value-add capability for relevant global health use cases	Understand eProcurement as a value-add capability for relevant global health use cases	Describe eProcurement as a value-add capability for relevant global health use cases	Evaluate eProcurement as a value-add capability for relevant global health use cases	Strategically analyse eProcurement as a value-add capability for relevant global health use cases

Appendix C

SAQA Structure

All registered learning programmes in South Africa are registered with the South African Qualifications Authority (SAQA). They provide a rich source of vocational and formal qualifications that are aligned to a nationally prescribed standard of progression levels, which also use a common language to describe exit level criteria and learning outcomes.

The authors used this baseline information to ensure there was consistency across all competency domains and job functions such that any other national standards could be overlaid onto the professionalisation framework to determine alignment, while ensuring completeness.

SAQA provides a country specific example of how other countries and organisations could use this professionalisation framework alongside an existing national educational framework or provide a starting point for holistic and thorough pathways to be developed where non exist.

What is the South African Qualifications Authority?

SAQA is the oversight body of the NQF (National Qualifications Framework) and the custodian of its values and quality character.

NQF objectives

The objectives of the NQF as outlined in the South African NQF Act No 67 of 2008 are to:

- Create a single integrated national framework for learning achievements
- Facilitate access to, and mobility and progression within, education, training and career paths
- Enhance the quality of education and training
- Accelerate the redress of past unfair discrimination in education, training and employment opportunities

What is the NQF?

Imagine a building plan of a ten-storey building. The NQF is like a plan of such a building with levels one to ten for learning. It stipulates standards for qualifications and part-qualifications. Apart from qualifications and part-qualifications, other information is also registered and recorded on the NQF. This includes professional designations and learner achievements, respectively.

What does the NQF look like?

If we were to visualise the NQF, it would look like the diagram below:

National Qualifications Framework				
Level	Sub-framework and qualification types			
10	Higher Education Qualifications Sub-framework	Doctoral Degree<br>Doctoral Degree (Professional)	*	Occupational Qualifications Sub-Framework
9		Master's Degree<br>Master's Degree (professional)	*	
8		Bachelor Honours Degree<br>Postgraduate Diploma<br>Bachelor's Degree	Occupational Certificate (Level 8)	
7		Bachelor's Degree<br>Advanced Diploma	Occupational Certificate (Level 7)	
6		Diploma<br>Advanced Certificate	Occupational Certificate (Level 6)	
5		Higher Certificate	Occupational Certificate (Level 5)	
4	General and Further Education and Training Qualifications Sub-Framework	National Certificate	Occupational Certificate (Level 4)	
3		Intermediate Certificate	Occupational Certificate (Level 3)	
2		Elementary Certificate	Occupational Certificate (Level 2)	
1		General Certificate	Occupational Certificate (Level 1)	

What does the NQF do?

As has been mentioned above, the NQF is like a map or guide that enables learners to chart their education and training path. For example, schooling in South Africa begins under the umbrella of General and Further Education and Training Qualifications Sub-Framework, better known as Basic Education.

At the end of Grade 9 a learner can either take the vocational route and go to a Technical and Vocational Education and Training (TVET) College or remain within the General and Further Education and Training Qualifications Sub Framework and work towards a National Senior Certificate at NQF Level 4.

Similarly, a learner who takes the Occupational Qualifications Sub-Framework route will obtain a National Certificate (Vocational) also pegged at NQF Level 4. These learners can continue to higher education or obtain other higher occupational qualifications. They can also move across the sub-frameworks.

The NQF was created to ensure that all this is possible without learners reaching 'dead-ends' in their education and training.

PEOPLE THAT DELIVER

People that Deliver
@Pplthatdeliver
