# Supplementary material for: Applying a Theory of Change for Human Resources Development in Public Health Supply Chains in Rwanda
Source: Glob Health Sci Pract. 2025 May 9;13(Suppl 1):e2300062. doi: 10.9745/GHSP-D-23-00062 (PMC12063746; doi:10.9745/GHSP-D-23-00062)

Supplement to: Meier E, Brown AN, McHenry B, Kabatende J, Gege Buki IK, Icyimpaye J. Applying a theory of change for human resources development in public health supply chains in Rwanda. Glob Health Sci Pract. 2024;12(Suppl 3):e2300062. https://doi.org/10.9745/GHSP-D-23-00062

PEOPLE THAT DELIVER

Building Human Resources for Supply Chain Management: A Theory of Change

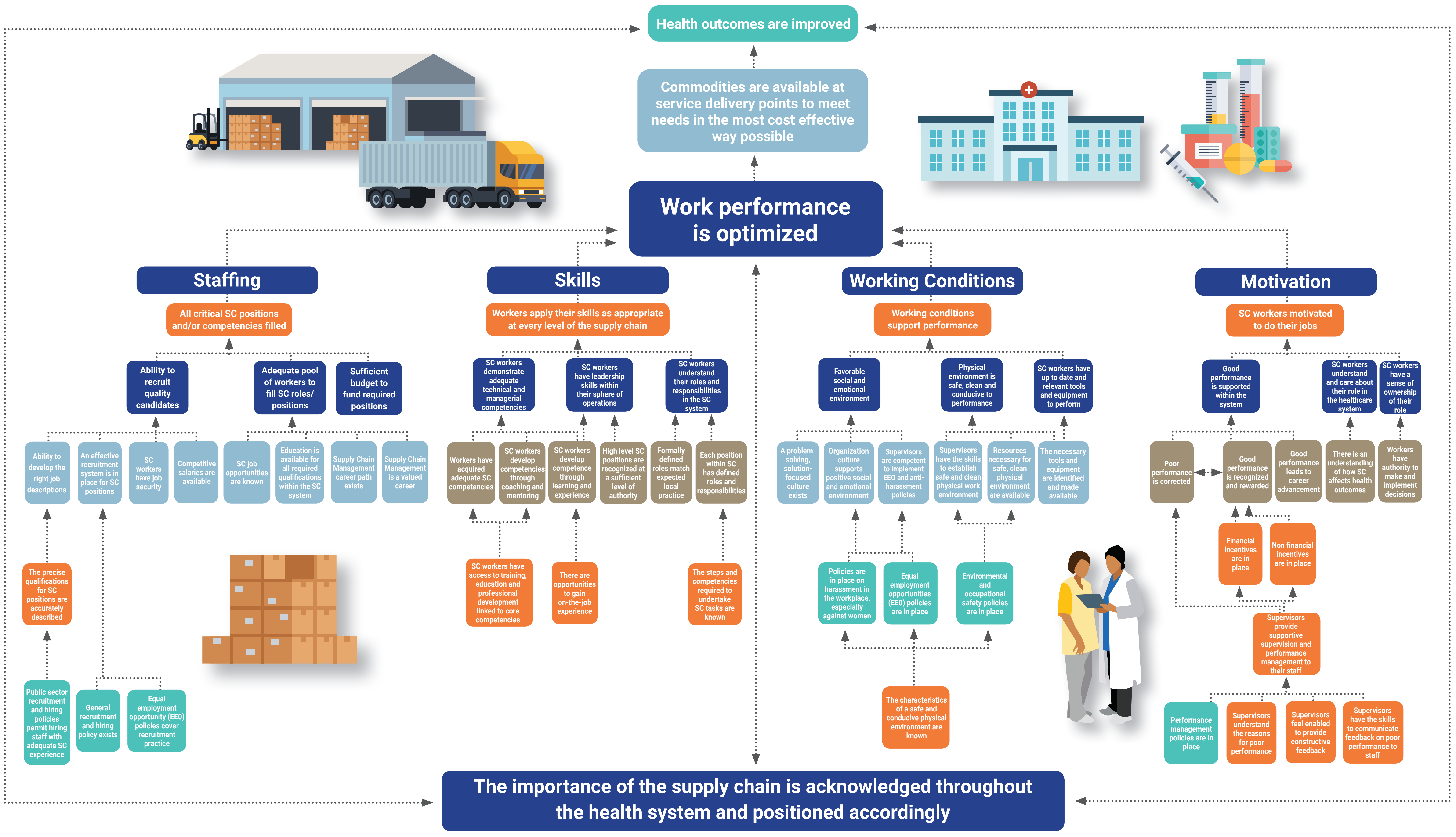

Supplement: GHSP-D-23-00062-Meier-Supplement1.pdf [file GHSP-D-23-00062-Meier-Supplement1.pdf]
